# Imputing Amino Acid Polymorphisms in Human Leukocyte Antigens

**DOI:** 10.1371/journal.pone.0064683

**Published:** 2013-06-06

**Authors:** Xiaoming Jia, Buhm Han, Suna Onengut-Gumuscu, Wei-Min Chen, Patrick J. Concannon, Stephen S. Rich, Soumya Raychaudhuri, Paul I.W. de Bakker

**Affiliations:** 1 Harvard-MIT (Massachusetts Institute of Technology) Division of Health Sciences and Technology, Boston, Massachusetts, United States of America; 2 Division of Genetics, Brigham and Women's Hospital, Harvard Medical School, Boston, Massachusetts, United States of America; 3 Program in Medical and Population Genetics, Broad Institute of Harvard and MIT, Cambridge, Massachusetts, United States of America; 4 Division of Rheumatology, Brigham and Women's Hospital, Harvard Medical School, Boston, Massachusetts, United States of America; 5 School of Medicine, University of Virginia, Charlottesville, Virginia, United States of America; 6 Partners HealthCare Center for Personalized Genetic Medicine, Boston, Massachusetts, United States of America; 7 Faculty of Medical and Human Sciences, University of Manchester, Manchester, United Kingdom; 8 Department of Epidemiology, University Medical Center Utrecht, Utrecht, The Netherlands; 9 Department of Medical Genetics, University Medical Center Utrecht, Utrecht, The Netherlands; University of Alabama at Birmingham, United States of America

## Abstract

DNA sequence variation within human leukocyte antigen (HLA) genes mediate susceptibility to a wide range of human diseases. The complex genetic structure of the major histocompatibility complex (MHC) makes it difficult, however, to collect genotyping data in large cohorts. Long-range linkage disequilibrium between HLA loci and SNP markers across the major histocompatibility complex (MHC) region offers an alternative approach through imputation to interrogate HLA variation in existing GWAS data sets. Here we describe a computational strategy, SNP2HLA, to impute classical alleles and amino acid polymorphisms at class I (*HLA-A*, -*B*, -*C*) and class II (-*DPA1*, -*DPB1*, -*DQA1*, -*DQB1*, and -*DRB1*) loci. To characterize performance of SNP2HLA, we constructed two European ancestry reference panels, one based on data collected in HapMap-CEPH pedigrees (90 individuals) and another based on data collected by the Type 1 Diabetes Genetics Consortium (T1DGC, 5,225 individuals). We imputed HLA alleles in an independent data set from the British 1958 Birth Cohort (*N* = 918) with gold standard four-digit HLA types and SNPs genotyped using the Affymetrix GeneChip 500 K and Illumina Immunochip microarrays. We demonstrate that the sample size of the reference panel, rather than SNP density of the genotyping platform, is critical to achieve high imputation accuracy. Using the larger T1DGC reference panel, the average accuracy at four-digit resolution is 94.7% using the low-density Affymetrix GeneChip 500 K, and 96.7% using the high-density Illumina Immunochip. For amino acid polymorphisms within HLA genes, we achieve 98.6% and 99.3% accuracy using the Affymetrix GeneChip 500 K and Illumina Immunochip, respectively. Finally, we demonstrate how imputation and association testing at amino acid resolution can facilitate fine-mapping of primary MHC association signals, giving a specific example from type 1 diabetes.

## Introduction

The major histocompatibility complex (MHC) region on the short arm of chromosome 6 harbors the human leukocyte antigen (HLA) genes. The HLA genes encode cell-surface proteins that present antigen peptides to the host immune system, and are among the most polymorphic genes in the human genome [1]. These genes have been prominently studied because of their large effect sizes in autoimmune diseases, infectious diseases, severe drug reactions, and transplant medicine [2]–[5]. In many instances, the observed HLA effects dwarf those of other associated variants throughout the rest of the genome [6].

The MHC is characterized by a unique evolutionary history. Its genetic structure is shaped not only by recombination, gene conversion and demography but also by natural selection [7]. One of the characteristic features of the MHC is the strong linkage disequilibrium (LD) among variants, often at considerable distances [8]. As a result, fine-mapping genotype-phenotype associations within the MHC to causal variants remains challenging.

While advances in high-throughput probe-based genotyping technologies have enabled systematic interrogation of DNA sequence variation through genome-wide association studies (GWAS), they have not been effective at querying variation within HLA genes. Probe-based methods for HLA genotyping have been limited in resolution due to the highly polymorphic nature of these genes. Strategies for direct typing of HLA alleles include sequence specific oligonucleotide (SSO) hybridization, capillary (Sanger) sequencing, and next-generation sequencing [9]. Unfortunately these approaches do not easily scale for large cohorts since they are labor-intensive, time-consuming and expensive.

As a potential way forward, investigators have developed methods to infer classical HLA alleles indirectly using intragenic SNP genotypes within the MHC. Initially, our group devised a simple approach using selected tag SNPs that are in strong LD with classical HLA alleles [8], [10]. Subsequently, more sophisticated approaches that model LD patterns of surrounding SNPs have been developed to impute classical HLA alleles [11]–[14]. Even if such predictions are not error-free, they are highly suitable for the re-interpretation of existing GWAS data, because imputation inaccuracy will generally result only in a power reduction to detect a statistical association but not in an increased type 1 error rate. In light of the enormous investment into GWAS in large numbers of samples, HLA imputation is likely to add significant value to SNP data that has already been generated [15].

An important limitation of existing HLA imputation methods, and of many previous studies, is that they are limited to classical HLA alleles and do not query functional coding variants *within* the HLA genes. For certain traits, specific amino acid positions within HLA molecules may play an important functional role. For example, the role of amino acid position 57 in HLA-DQβ1 for type 1 diabetes susceptibility has been long established [16]. In addition, our group recently identified a key role for amino acid position 97 in *HLA-B*, which can account for almost all known classical allele associations with HIV control [17].

**Figure 1:**
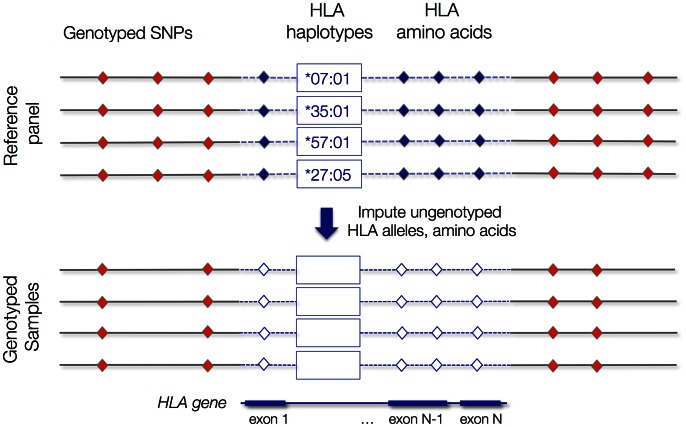
Overview of the SNP2HLA imputation procedure. The reference panel (top) contains SNPs in the MHC, classical HLA alleles at the class I and class II loci, and amino acid sequences corresponding to the 4-digit HLA types at each locus. For a data set with genotyped SNPs across the MHC (bottom), we use the reference panel to impute classical alleles and their corresponding amino acid polymorphisms.

To identify potentially causal variation within HLA genes, we present here a method, SNP2HLA, for imputing classical HLA alleles as well as amino acid polymorphisms in the HLA proteins from SNP genotype data with the Beagle software package [18] (**Figure 1**). In order to characterize the tradeoffs involved in HLA imputation, we consider four scenarios covering two reference panels (with different sample sizes) and two SNP data sets (with different SNP densities). This study design allows us to evaluate the impact of sample size of the reference panel and the impact of SNP genotyping density on the imputation quality. We also assess the accuracy of imputations at individual amino acid polymorphisms. Finally, we demonstrate that we can reproduce known HLA allelic effects from genotyped SNP data in type 1 diabetes from the publicly available Wellcome Trust Case Control Consortium data set [19].

## Results

### Reference Panels for Imputation

We constructed two reference panels based on genotyping data collected in individuals of European ancestry (**Table 1**). The HapMap-CEPH panel contains 3,924 SNPs (genotyped with Illumina GoldenGate) and 4-digit classical HLA types for *HLA-A*, -*B*, -*C*, -*DQA1*, -*DQB1* and -*DRB1* for 90 unrelated individuals (180 haplotypes) [8]. The Type 1 Diabetes Genetics Consortium (T1DGC) panel contains 5,868 SNPs (genotyped with Illumina Immunochip) and 4-digit classical HLA types for *HLA-A*, -*B*, -*C*, -*DPA1*, -*DPB1*, -*DQA1*, -*DQB1* and -*DRB1* for 5,225 unrelated individuals (10,450 haplotypes). The T1DGC panel contains more unique HLA alleles and amino acid polymorphisms because of its significantly larger sample size.

**Table 1:** Overview of the HapMap-CEPH and T1DGC reference panels and the B58BC validation panel.

	Reference panel		Validation panel	
Sample set	HapMap-CEPH	Type 1 Diabetes Genetics Consortium (T1DGC)	British 1958 Birth Cohort (B58BC)	
Sample size	90	5,225	918	
Genotyping platform	Illumina GoldenGate	Illumina Immunochip	Affymetrix 500 K	Illumina Immunochip
Number of SNPs in MHC	4,791	7,135	916	7,563
Number of SNPs passing QC	3,924	5,868	890	5,893
Number of 4-digit classical HLA alleles				
* HLA-A*	17	50	25	
* HLA-B*	29	97	40	
* HLA-C*	19	33	20	
* HLA-DPA1*	–	7	–	
* HLA-DPB1*	–	34	–	
* HLA-DQA1*	7	8	–	
* HLA-DQB1*	14	18	17	
* HLA-DRB1*	23	51	34	
Number of polymorphic positions				
* *Intragenic SNPs	915	1,101	858	
* *Amino acids	321	399	289	
* *Indels	42	176	37	
Total number of biallelic markers	5,986	8,961	5,112	

The MHC region is defined here as 29–34 Mb on chr6 (hg17). Sample size is based on unrelated (founder) individuals. The number of unique 4-digit classical HLA alleles at each locus is shown for each data set. Intragenic SNPs, amino acids, and indels represent unique polymorphic positions as defined by the classical HLA types in each data set.

### Validation Panel for Benchmarking

To benchmark the HLA imputations by SNP2HLA, we used 918 individuals from the British 1958 Birth Cohort (B58BC) with gold-standard 4-digit HLA types at *HLA-A, -B*, *-C*, -*DQB1* and -*DRB1*, and SNP genotype data collected on both the Affymetrix 500 K and Illumina Immunochip. The SNP genotyping density varied widely between the data sets across the MHC region (**Figure S1 in File S1**), affecting the effective number of SNPs that could be used for imputation. For example, there were only 487 SNPs present on the Affymetrix 500 K that overlapped with the T1DGC reference panel, in contrast with the 4,794 SNPs in common between the Immunochip data from the B58BC validation panel and the T1DGC reference panel (**Table 2**).

**Table 2:** Imputation accuracy measured as the genotype concordance for two- and four-digit classical HLA alleles measured in the British 1958 Birth Cohort (B58BC, 918 individuals) as a function of reference panel (HapMap or T1DGC) and genotyping platform (in B58BC).

	HapMap reference panel		T1DGC reference panel	
Genotyping platform	Affymetrix 500 K	Illumina Immunochip	Affymetrix 500 K	Illumina Immunochip
Genotyped SNPs	916	7563	916	7563
Overlapping SNPs	332	2466	487	4794
*4-digit resolution accuracy*				
*HLA-A*	89.9%	95.4%	97.2%	98.1%
*HLA-B*	83.0%	88.2%	94.7%	96.8%
*HLA-C*	87.2%	90.7%	96.1%	96.9%
*HLA-DQB1*	72.3%	71.8%	95.5%	98.3%
*HLA-DRB1*	72.6%	84.3%	89.3%	93.3%
All loci	81.3%	86.5%	94.7%	96.7%
*2-digit resolution accuracy*				
*HLA-A*	89.9%	95.1%	98.4%	98.7%
*HLA-B*	83.0%	90.1%	96.1%	98.2%
*HLA-C*	87.6%	90.9%	96.8%	97.2%
*HLA-DQB1*	80.2%	78.0%	97.7%	99.2%
*HLA-DRB1*	82.4%	91.0%	95.6%	98.5%
All loci	84.6%	89.0%	96.9%	98.4%

Comparisons were made only if both alleles were typed at the same resolution (two- or four-digit). Accuracy was based on the same set of variants, allowing a direct and fair comparison between reference panels and genotyping platforms.

### HLA Imputation

Using the HapMap-CEPH reference panel, we imputed in each of the 918 B58BC individuals dosages for 70 classical 2-digit alleles and 109 classical 4-digit alleles at *HLA-A*, *-B*, *-C*, -*DQA1*, -*DQB1* and -*DRB1*, and 321 polymorphic amino acid positions, 915 intragenic SNPs and 42 indels (**Table 1**). Using the T1DGC reference panel, we imputed 126 classical 2-digit alleles and 298 classical 4-digit alleles at *HLA-A*, *-B*, *-C*, -*DPA1,* -*DPB1, -DQA1*, -*DQB1* and -*DRB1*, and 399 polymorphic amino acid positions, 1,101 intragenic SNPs and 176 indels. (The HapMap-CEPH panel did not contain HLA types for the *HLA-DPA1* and *HLA-DPB1* loci.).

### Imputation Accuracy of Classical HLA Alleles

**Figure 2:**
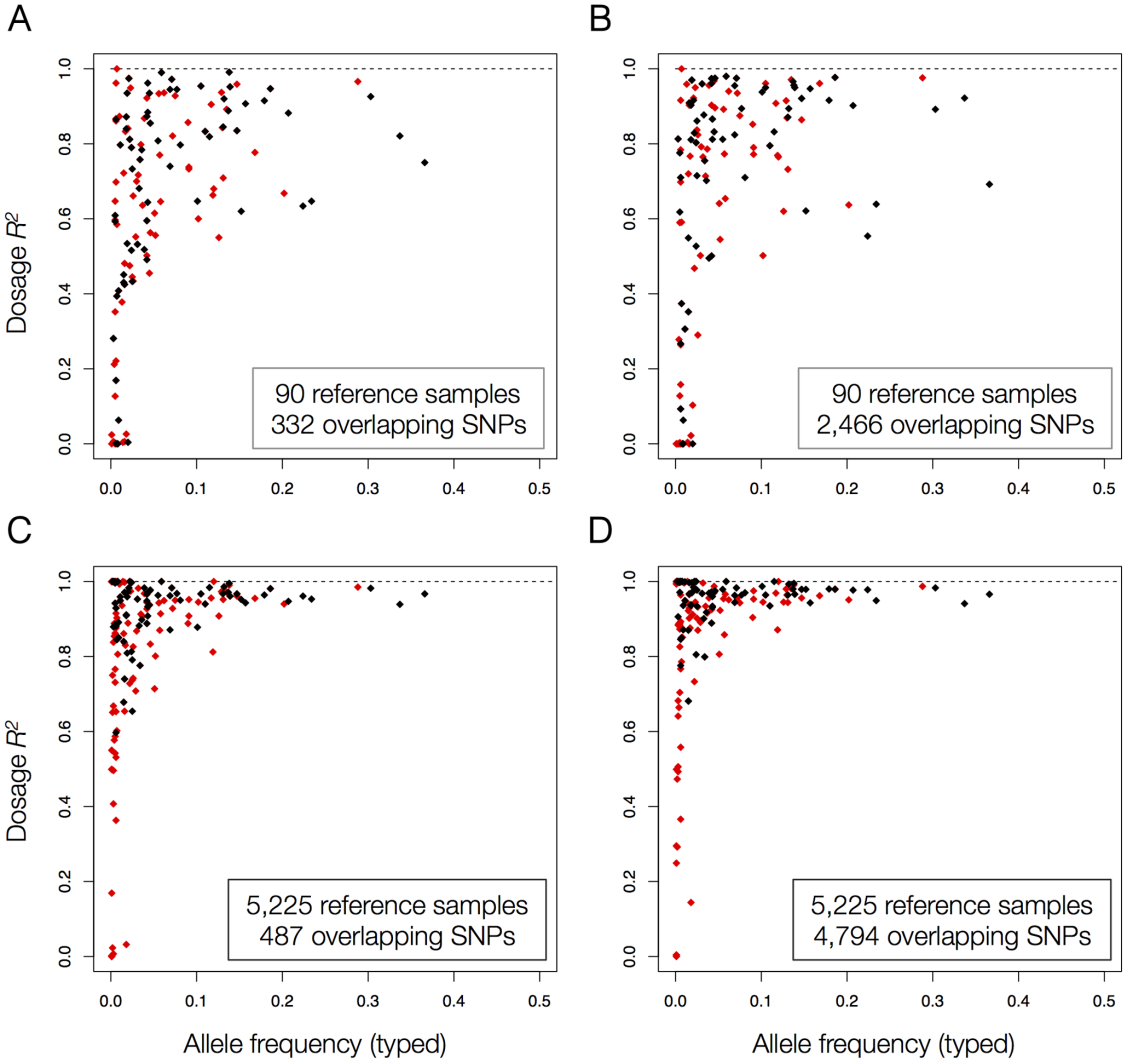
Correlation between imputed and typed dosages (*r* ^2^_dosage_) of classical HLA alleles in the B58BC as a function of typed allele frequency for imputation from the (a) Affymetrix 500 K or (b) Illumina Immunochip platform using the HapMap-CEPH reference panel, and imputation from the (c) Affymetrix 500 K or (d) Illumina Immunochip platform using the T1DGC reference panel. Black points indicate 2-digit HLA alleles. Red points indicate 4-digit HLA alleles.

For both reference panels, we observed that there was generally high correlation between the imputed and typed HLA allele frequencies (*r* ^2^_freq_>0.99 for 2-digit and *r* ^2^_freq_>0.96 for 4-digit alleles, **Figure S2 in File S1**). Using the larger T1DGC panel, SNP2HLA achieved high correlation between the imputed and typed dosages for common HLA alleles (**Figure 2**). At 4-digit resolution, we imputed 44 of 46 class I alleles and 24 of 26 class II alleles with >1% frequency with high accuracy (*r* ^2^_dosage_>0.8, **Table S1 in File S1**). In terms of genotype concordance, SNP2HLA achieved 81.3% and 86.5% accuracy for 4-digit HLA alleles using Affymetrix 500 K and Illumina Immunochip, respectively, when imputing from the HapMap-CEPH reference panel (**Table 2**). This improved significantly for the larger T1DGC panel, where SNP2HLA obtained an accuracy of 94.7% and 96.7% using Affymetrix 500 K and Illumina Immunochip, respectively (**Table 2**). Overall, these results indicate much better performance for the T1DGC reference panel compared to the HapMap-CEPH reference panel.

We compared these results to published benchmarking results for HLA*IMP, a widely used software tool for imputation of classical HLA alleles [14]. Although we did not perform a direct head-to-head comparison with identical training and testing data sets, both methods appear to deliver comparable imputation accuracy at 4-digit resolution (**Table S2 in File S1**).

### Imputation Accuracy of Amino Acid Polymorphisms

**Figure 3:**
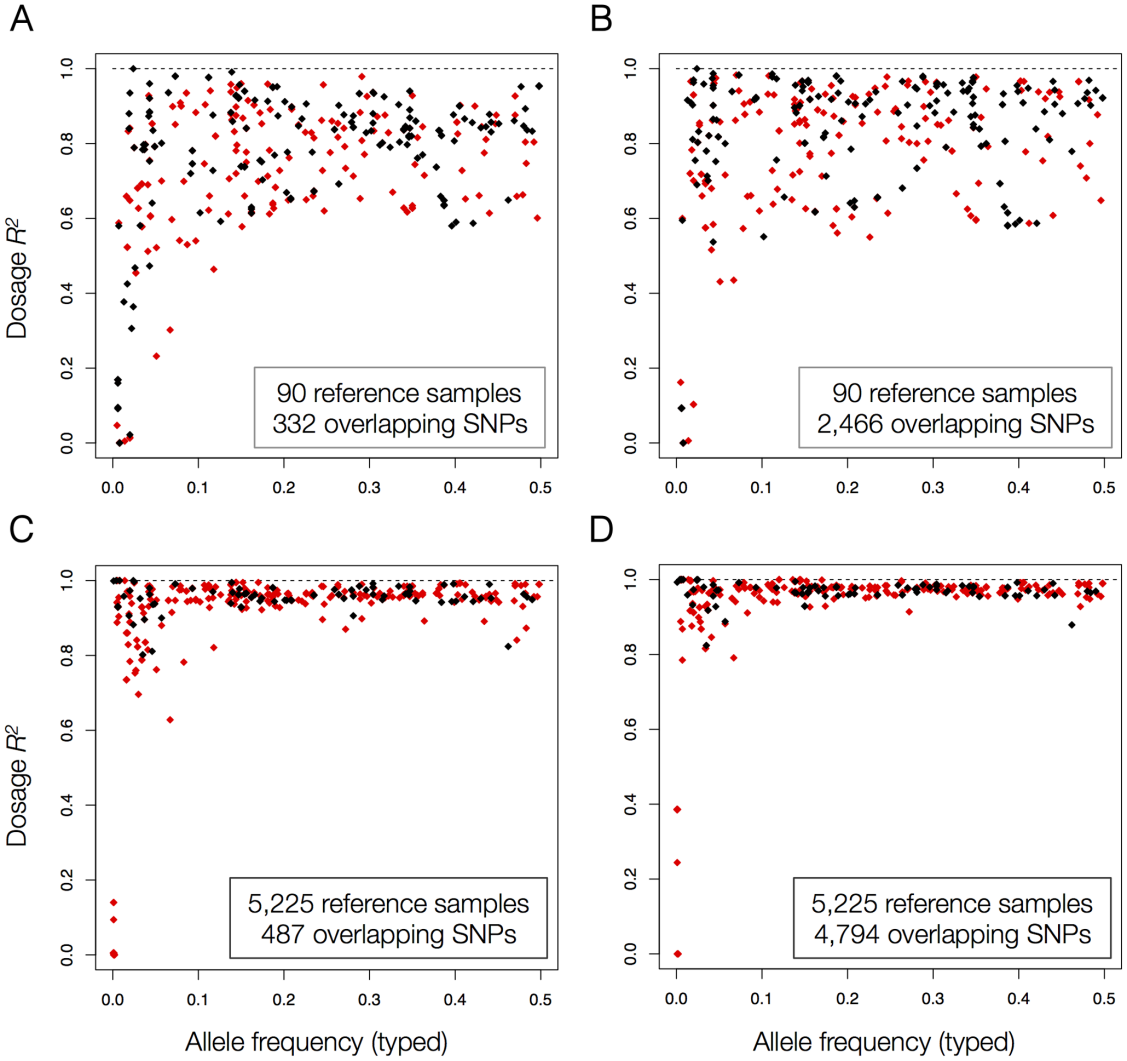
Correlation between imputed and typed dosages (*r* ^2^_dosage_) of polymorphic amino acids in the B58BC as a function of typed allele frequency for imputation from the (a) Affymetrix 500 K or (b) Illumina Immunochip platform using the HapMap-CEPH reference panel, and imputation from the (c) Affymetrix 500 K or (d) Illumina Immunochip platform using the T1DGC reference panel. Black points indicate bi-allelic positions. Red points indicate poly-allelic positions.

Next, we assessed the imputation quality of the polymorphic amino acid positions by comparing imputed dosages for individual amino acid alleles to the corresponding dosages from the gold-standard 4-digit HLA types in the same 918 B58BC individuals. We observed a near-perfect correlation between the imputed and genotyped amino acid frequencies for both reference panels (**Figure S3 in File S1**). In terms of the correlation between imputed and typed allelic dosages, we found that 48.0% and 65.7% of amino acid positions achieved *r* ^2^_dosage_>0.8 using Affymetrix 500 K and Illumina Immunochip, respectively, when imputing from the HapMap-CEPH reference panel. Performance improved again significantly with the larger T1DGC reference panel, where 99.2% and 99.3% of polymorphic amino acid positions reached *r* ^2^_dosage_>0.8 using Affymetrix 500 K and Illumina Immunochip, respectively (**Figure 3**). In terms of genotype concordance, SNP2HLA achieved 93.9% and 94.2% accuracy with the HapMap-CEPH reference panel starting from Affymetrix 500 K and Illumina Immunochip, respectively (**Tables S3 and S4 in File S1**). With the larger T1DGC panel, this improved to 98.6% and 99.3% accuracy for Affymetrix 500 K and Illumina Immunochip, respectively (**Tables S3 and S4 in File S1**). These results demonstrate again better performance for the larger T1DGC reference panel, and highlight that the individual amino acid positions can be imputed with great accuracy.

To evaluate differences in imputation performance between HLA loci, we calculated the average dosage *r^2^* per polymorphic position. Starting from Illumina Immunochip data using the T1DGC reference panel, the imputation performance was consistently high across the class I and class II loci: *HLA-A* (*r^2^ = *0.98), *HLA-B* (*r^2^ = *0.97), *HLA-C* (*r^2^ = *0.96), *HLA-DQB1* (*r^2^* = 0.97), and *HLA-DRB1* (*r^2^* = 0.96), even though a limited number of amino acid positions were more difficult to impute (**Tables S3 and S4 in File S1**).

### Imputation in Non-European Samples

We next assessed the imputation performance in non-European populations. To test this, we imputed HLA alleles in three HapMap panels (CEU/CEPH, YRI, CHB+JPT) using the T1DGC reference panel. Using the gold-standard HLA type data in the HapMap samples [8], 4-digit HLA imputation accuracy was highest (98.3% over all HLA loci) in the CEU/CEPH samples, but was considerably lower in the YRI panel (72.9%), and in the CHB+JPT panel (86.4%) (**Table 3**). Strikingly, imputation performance was quite variable across HLA loci in non-European populations. In the CHB+JPT panel, imputation accuracy was highest at *HLA-A* (98.1%) and *HLA-DQB1* (96.5%), but low in *HLA-B* (65.5%) and *HLA-C* (68.8%). In the YRI panel, imputation performance was high in *HLA-C* (98.4%) and *HLA-DQB1* (96.1%), but very low at *HLA-DRB1* (20.3%) and *HLA-A* (69.9%). These results reinforce the need for large population-specific reference panels in order to achieve high quality HLA imputations.

**Table 3:** Imputation accuracy of classical alleles at 4-digit resolution across worldwide populations.

	CEU/CEPH	YRI	CHB+JPT
*HLA-A*	99.1%	69.9%	98.1%
*HLA-B*	96.8%	90.5%	65.6%
*HLA-C*	99.1%	98.4%	68.8%
*HLA-DQA1*	98.5%	64.9%	96.3%
*HLA-DQB1*	99.1%	96.1%	96.5%
*HLA-DRB1*	96.9%	20.3%	92.3%
All loci	98.3%	72.9%	86.4%

Imputations were performed using the T1DGC reference panel, and accuracy (as measured by genotype concordance) in the three HapMap panels (CEU/CEPH, YRI and CHB+JPT) with the publicly available gold-standard HLA genotype data [8]. Accuracy is consistently high across all loci in Europeans (CEU/CEPH), but much worse in the African (YRI) and East-Asian (CHB+JPT) populations.

### Calibration of Posterior Probabilities

Next, we evaluated how well the posterior probabilities for imputed variants tracked with imputation accuracy. We observed a high correlation between imputation dosage (probabilistic representation of the number of predicted alleles) and the true genotype dosage (0, 1, or 2), especially for imputations with high confidence (**Figure S4 in File S1**). Moreover, there are more highly confident calls (reflected by fewer imputation dosages between 0 and 1 and between 1 and 2) for imputations derived from the T1DGC reference panel compared to the HapMap-CEPH panel. These results suggest that probabilistic dosages correlate better with true genotypes than best-guess genotypes, and should be taken into account for subsequent statistical analyses and association testing.

### HLA Imputation in WTCCC Type 1 Diabetes

Lastly, we wanted to evaluate the potential for SNP2HLA to reproduce HLA associations from a GWAS dataset. To this end, we used the WTCCC type 1 diabetes cases and controls [19]. The cases consist of 1,963 individuals and the controls consist of 2,939 individuals, all genotyped with the Affymetrix 500 K array. The controls include the same 918 individuals from the B58BC panel that were used for the imputation benchmark above. After quality control, 511 SNPs remained that overlapped with the T1DGC reference panel.

**Figure 4:**
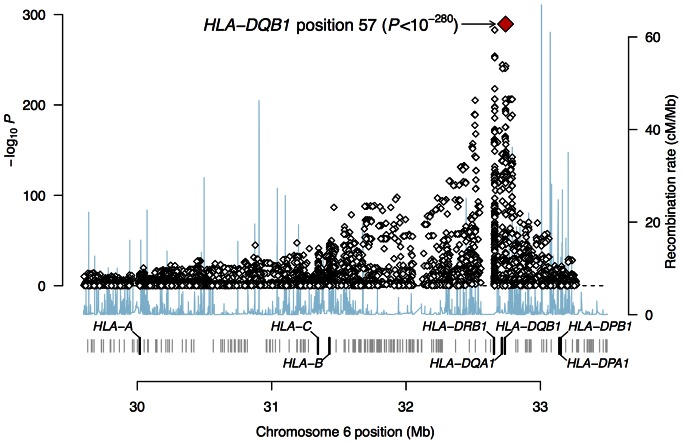
Association analysis of WTCCC type 1 diabetes data. We imputed classical HLA alleles and polymorphic amino acids in 1,963 cases and 2,939 controls using the T1DGC reference panel, and tested all variants for association with logistic regression. Of all variants tested, the top hit maps to amino acid position 57 in HLA-DQβ1, consistent with a previous study [16].

We applied SNP2HLA to impute all markers, and then tested them for association. Among all of these markers, the top signal was HLA-DQβ1 amino acid position 57 (*P*<10^−280^) (**Figure 4**). This highly significant amino acid position is historically well known as a potential causal risk factor for type 1 diabetes [16]. This demonstrates that the value of SNP2HLA to leverage large GWAS data sets to impute individual amino acids and to pinpoint the location of potentially causal amino acid sites.

**Figure 5:**
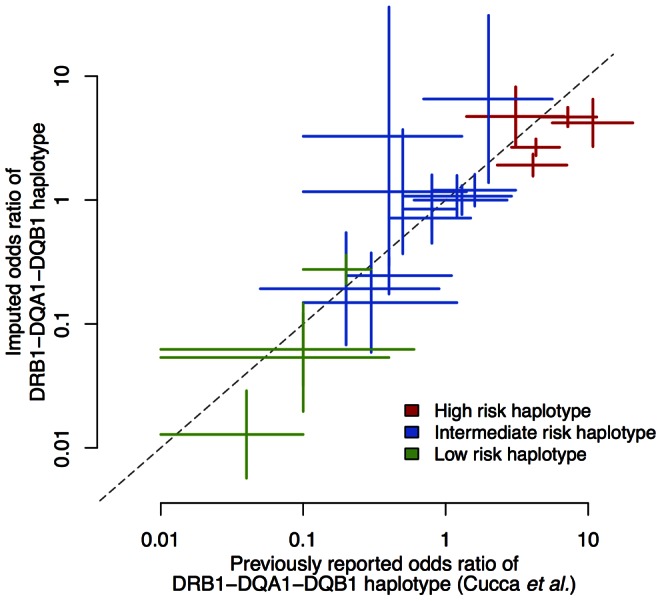
Haplotype risk analysis of WTCCC type 1 diabetes data. We assessed the risk of haplotypes spanning *HLA-DRB1*, *HLA-DQA1* and *HLA-DQB1*, and compared these to the published risk estimates from an independent study [20]. The published odds ratios were based on transmission/non-transmission of alleles from familial data, while our odds ratios were estimated from case/control data. We used the same classification scheme by dividing haplotypes into three risk groups. The odds ratios are computed with respect to the DRB1*01-DQA1*0101-DQB1*0501 haplotype.

We also performed haplotype analysis in this dataset to test if the haplotype effect sizes are concordant with the literature. We specifically calculated the risk estimates for haplotypes of classical alleles spanning *HLA-DRB1*, *HLA-DQA1* and *HLA-DQB1*, as these have been estimated by others [20]. Although the previously reported effects were odds ratios based on transmission/non-transmission of alleles (ORT) from familial data, we expect that the estimated odds ratios in the WTCCC case-control data will be concordant as long as the imputations and phasing are accurate. Indeed, our results show that the haplotype effect sizes are highly concordant within the range of sampling error between two different datasets (**Figure 5** and **Table S5 in File S1**). The haplotypes that are known as “high risk” confer high risk in our analysis, and haplotypes known as “low risk” confer similarly low risk. These empirical results demonstrate the validity of our imputations and the inferred HLA haplotypes.

## Discussion

We have developed a method, SNP2HLA, to impute HLA amino acids and classical HLA alleles using SNP genotype data within the MHC region. With a large reference panel we have demonstrated that our approach can yield high-quality imputations of classical HLA alleles and coding variation within the HLA genes. Even with relatively modest SNP genotyping coverage (for example, first-generation GWAS arrays), the long-range LD patterns in the region make it possible to accurately impute HLA variants.

The imputation quality of SNP2HLA is determined primarily by the size and quality of the reference panel rather than the SNP genotyping density. Accuracy is generally lower for low-frequency or rare alleles, which is similar to experience with imputation methods outside of the MHC region [21]. This limitation may be mitigated by using larger reference panels containing multiple observations (that is, haplotypes) of low-frequency alleles. As a result, application of SNP2HLA with a large T1DGC reference panel with >10,000 haplotypes achieved high imputation accuracy for both classical HLA alleles and amino acids. We could not assess performance at *HLA-DPA1*, *HLA-DPB1*, and *HLA-DQA1*, since these genes were not available to us for evaluation. Because *HLA-B* and *HLA-DRB1* are the most polymorphic genes in class I and II (and therefore considered the most difficult to impute), our results may be slightly conservative with respect to *HLA-DQA1*, -*DPA1* and -*DPB1*.

The density of SNPs typed within the MHC has a modest but measureable effect on HLA imputation quality. The Immunochip, which has between 2,500 and 5,000 overlapping SNPs with both HapMap and T1DGC reference panels, consistently showed higher imputation accuracy compared to the Affymetrix 500 K array, which only shares 300–400 SNPs with these reference panels. This effect is most prominent at the class II locus *HLA-DRB1*, where Immunochip showed an average improvement of 6% in four-digit HLA accuracy over the 500 K array, compared to an average improvement of 0.9% at class I loci. This may be due to the relatively shorter spans of linkage disequilibrium within the class II region compared to class I, rendering individual distal SNPs within the class II region somewhat less informative about the haplotypes.

The power of our approach is not only in highly accurate imputation of HLA classical alleles, but also in allowing individual amino acid polymorphisms to be tested. This gives users the ability to query variation within HLA genes for association in an entirely different way than previously applied. We demonstrated the potency of this approach using the WTCCC type 1 diabetes data [19]. By simultaneously testing HLA alleles, amino acids, and SNPs, we were able to pinpoint the HLA-DQβ1 position 57 as the top signal, which is recognized as the major risk factor for type 1 diabetes [16]. Another strength of SNP2HLA is that phased haplotypes are obtained. Using the WTCCC type 1 diabetes data, we were able to accurately assess the risk of haplotypes spanning *HLA-DRB1*, *HLA-DQA1* and *HLA-DQB1*. Our estimates of effect sizes were consistent with published effect sizes.

One limitation of our imputation method (and of all other imputation methods) is that the reference panel should properly represent the target population. We observed that imputation quality is inconsistent when imputing HLA variants in a non-European population using a predominantly European reference panel. Currently, we are not aware of a large data set with SNP and high-resolution HLA types in non-European populations, and argue that resources should be made available to generate multiethnic panels to enable HLA imputation in worldwide populations, including admixed populations. In constructing additional reference panels, each investigator will have to weigh the benefits of imputing rare alleles with greater accuracy against the additional resources required to expand the sample size of the reference panel.

There are other limitations of our method. First, there are known limitations to established methods for HLA typing [22]. As a result, there may be errors in the reference panel that may limit imputation accuracy, and errors in the gold standard that limit the evaluation of accuracy. Without commenting on the intrinsic error rate of classical HLA typing itself, our results show that LD-based imputation can achieve high quality using a large reference panel. Second, while this method enables interrogation of polymorphisms at the widely studied HLA loci, it does not capture variation at hundreds of other genes present in the MHC [23]–[25]. In many instances, variation at these genes is captured by SNPs throughout the MHC, but comprehensive interrogation of the entire region will ultimately require high-throughput sequencing, making imputation redundant.

The immense volume of data generated from recent GWAS provides an excellent opportunity to apply imputation techniques to fine-map MHC associations to classical alleles and amino acids of the HLA loci. We and others have previously demonstrated the potential of HLA imputation for a wide range of phenotypes including host control of HIV-1 replication [17], [26], rheumatoid arthritis [27], ulcerative colitis [28], primary biliary cirrhosis [29], psoriasis [30], ankylosing spondylitis [31], multiple sclerosis [32], liver carcinoma [33], Hodgkin lymphoma [34], carbamazepine-induced hypersensitivity [35], and myasthenia gravis [36]. For autoimmune or inflammatory diseases, the identification of classical allele associations or the fine-mapping of specific amino acid positions may facilitate the evaluation of specific peptides as antigens through binding assays and molecular modeling. For drug-induced hypersensitivity, a molecular model has recently been proposed that might explain how specific drug binding to the HLA pocket can perturb the T cell repertoire in an individual and cause T-cell mediated hypersensitivity [5]. Regardless of the underlying biology, we believe that imputation approaches for the MHC can add significant value to already existing data sets.

## Materials and Methods

### Reference Data

We constructed the HapMap-CEPH reference panel with MHC genotype data as described previously consisting of 182 individuals (29 extended families containing 45 unrelated parent-offspring trios) of European ancestry from the Centre d’Etude du Polymorphisme Humain (CEPH) collection [8]. Genotype data included 4,791 SNPs within the MHC region (chr6: 29–34 Mb) assayed using the Illumina GoldenGate platform and classical types for *HLA-A*, -*B*, -*C*, -*DQA1*, -*DQB1*, and -*DRB1* at four-digit resolution. We corrected a small number of HLA typing errors in these samples using next-generation 454 sequencing at the class I loci [22]. Of the CEPH individuals, we kept only founder individuals on the basis of the known familial relationships.

We constructed the T1DGC reference panel based on data collected in 5,225 unrelated individuals by the Type 1 Diabetes Genetics Consortium (T1DGC). Genotype data included 7,135 SNPs within the MHC region assayed with the Illumina Immunochip platform, and classical types for *HLA-A*, -*B*, -*C*, -*DQA1*, -*DQB1*, -*DPA1, -DPB1 and -DRB1* at four-digit resolution. For both reference panels, we used the software package PLINK [37] to remove SNPs with low minor allele frequency (<1%), high proportion of missing genotypes (>5% across individuals), and out of Hardy-Weinberg equilibrium (*P*<10^−6^). We used the KING software to test for relatedness between all individuals using the genome-wide Immunochip data, and kept only unrelated individuals [38]. We also performed principal components analysis on the same data, and confirmed that the overwhelming majority of the T1DGC individuals are of European ancestry (as judged by overlap with European ancestry reference populations from HapMap). The T1DGC reference panel can be obtained from the NIDDK repository at https://www.niddkrepository.org/niddk/home.do.

A key step in our approach is to impute not only classical HLA alleles but also amino acid polymorphisms and SNPs. First, we defined binary markers that correspond to the presence and absence of each unique 2- and 4-digit HLA allele. Second, we extracted the unique DNA and amino acid sequences for all observed HLA alleles from the EMBL-EBI Immunogenetics HLA Database [39] (http://www.ebi.ac.uk/imgt/hla/), and encoded polymorphic nucleotide and amino acid positions as binary markers in the reference panel. For a multi-allelic position, we added a binary marker for each allele. For example, if one amino acid position has three different alleles, we encoded the position using three binary markers each corresponding to the presence and absence of each allele. We also encoded separately insertions, deletions or truncations using binary markers. The purpose of converting all genetic variations into binary markers is to provide a basic unit that can be flexibly tested in the downstream association analysis. We removed markers (HLA alleles, amino acid positions, etc.) with very low allele frequency (<0.01%). We used Beagle [18] to phase genotype data into individual haplotypes, taking into account familial relationships wherever available. The procedure for generating a phased reference panel is fully implemented in our software and made available. Overall, the HapMap-CEPH panel comprises 180 haplotypes and the T1DGC panel 10,450 haplotypes.

### Imputation of Classical HLA Alleles and Amino Acids

Given SNP data of sample individuals, we imputed HLA types using the HapMap or T1DGC extended reference panel. First, we extracted SNPs located within the MHC region (chr6: 29–34 Mb on build 36/hg18), removed SNPs with minor allele frequency <2.5%, and checked the data to ensure that each SNP is oriented on the same strand as the reference panel. We used Beagle to impute all missing SNPs, classical HLA alleles, and amino acid polymorphisms using default parameters (10 iterations of phasing/imputation, testing 4 pairs of haplotype pairs for each individual at each iteration), but allowing a larger window size (maxwindow = 2000) for the Illumina Immunochip than for the Affymetrix 500 K chip (maxwindow = 1000). The output includes posterior probabilities and allelic dosages for each imputed variant, best-guess genotypes and phased haplotypes for each individual.

### Evaluation of Imputation Performance

To validate our imputation method, we utilized genotype data in 918 individuals from the British 1958 Birth Cohort [40]. These individuals have primarily Northern and Western European ancestry, as confirmed by principal components analysis in the WTCCC data [19]. Data for the British 1958 Birth Cohort were obtained from the European Genome-phenome Archive (EGA) at https://www.ebi.ac.uk/ega/. These individuals were genotyped using the Affymetrix GeneChip 500 K platform (with 916 SNPs in the MHC) and the Illumina Immunochip platform (with 7,563 SNPs in the MHC). In addition, these same subjects have gold-standard 4-digit HLA genotypes (at *HLA-A*, *HLA-B*, *HLA-C*, *HLA-DRB1* and *HLA-DQB1*) generated by the Juvenile Diabetes Research Foundation/Wellcome Trust Diabetes and Inflammation Laboratory.

For each imputation scenario, we calculated the imputation accuracy at each HLA locus by summing across all individuals the dosage of each true HLA allele in the individual, and divided by the total number of observation (i.e. number of chromosomes).
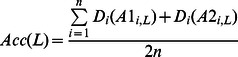
where *Acc(L)* represents the imputation accuracy at HLA locus *L*, where *L* might be a classical HLA locus (e.g. *DRB1*) or an individual polymorphic nucleotide or amino acid site. The parameter *n* denotes the number of individuals, *D_i_* represents the imputed dosage of an allele in individual *i*, and alleles *A1_i,L_* and *A2_i,L_* represent the true (gold standard) HLA types for individual *i* at locus *L*. If the individual was homozygous for a single allele (defined by A1), we only included the A1 term in the calculation. This scheme allows uncertain but partially correct imputations to contribute to the overall accuracy.

To evaluate imputation performance in individual HLA alleles and amino acids, we calculated the *r^2^* correlation between imputed and typed dosages for all HLA variants (encoded as bi-allelic markers). For amino acid positions with two alleles, we used the Pearson product moment correlation coefficient for two variables *x* and *y,* which denote the imputed and typed dosages respectively in *n* individuals.
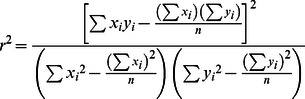



For amino acid positions with more than two alleles, we used a variation of the Pearson *r^2^* formula to determine the *R^2^* correlation between vectors of imputed and typed dosages, where each vector contains the dosages for different amino acid alleles at a specific position.
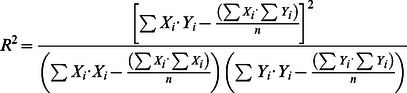
where *X_i_* represents the vector of imputed dosages for amino acids at a single position for individual *i*, and *Y_i_* represents the vector of typed dosages for amino acids at a single position for individual *i* across *n* individuals in the independent data set.

### Association Testing

We obtained the WTCCC genotype-phenotype data from the European Genotype Archive (http://www.ebi.ac.uk/ega), and imputed classical alleles and amino acids using the T1DGC reference panel. After imputation, we checked that the cumulative dosage of classical alleles of a given HLA locus summed to ∼2 for each individual. We used logistic regression modeling to test the allelic dosages of all imputed variants encoded by the T1DGC reference panel for association to disease status. By simultaneously testing all markers including HLA alleles, amino acids, and SNPs, we aim to avoid possible bias in the interpretation that can happen if we only examine one type of marker such as HLA alleles, since it is generally unknown *a priori* which variations are driving the association. To test individual amino acid positions, we test a model with all amino acid alleles of a given position, fitting individual effects for each of the alleles. The statistical significance is evaluated by calculating the deviance (−2 × log likelihood) of the alternative model compared to the null model.

### Obtaining SNP2HLA

Instructions for obtaining SNP2HLA and the HapMap and T1DGC reference panels can be found at http://www.broadinstitute.org/mpg/snp2hla/. Beagle should be obtained separately from the web site http://faculty.washington.edu/browning/beagle/beagle.html.

## Supporting Information

File S1 **Tables S1–S5. Figures S1–S4.**
(PDF)
