# Supplementary material for: Imputing Amino Acid Polymorphisms in Human Leukocyte Antigens
Source: PLoS One. 2013 Jun 6;8(6):e64683. doi: 10.1371/journal.pone.0064683 (PMC3675122; doi:10.1371/journal.pone.0064683)

Table S1: Imputation performance for two- and four-digit HLA alleles is shown for four imputation scenarios, each using a large (T1DGC) or small (HapMap CEU) reference panel, and a high-density (Illumina Immunochip) or low-density (Affymetrix 500K) SNP chip. Allele frequencies are shown for imputed and gold standard HLA types in 918 individuals from the British 1958 Birth Cohort. R^2 correlation (between imputed and genotyped dosages) and accuracy (genotype concordance) across individuals is provided for each classical allele at *HLA-A*, *-B*, *-C*, *-DQB1*, and *-DRB1*. Accuracy was based on the same set of variants, allowing a direct and fair comparison between reference panels and SNP chips.

		HapMap-CEPH (n=90)						T1DGC (n=5,225)					
		Affymetrix 500K			Illumina Immunochip			Affymetrix 500K			Illumina Immunochip		
**Allele**	**frq**	**frq**	***r2***	**accuracy**	**frq**	***r2***	**accuracy**	**frq**	***r2***	**accuracy**	**frq**	***r2***	**accuracy**
A*01	18.6%	18.9%	0.95	98.6%	18.5%	0.98	99.4%	18.5%	0.98	99.6%	18.5%	0.98	99.5%
A*01:01	18.6%	18.9%	0.95	98.6%	18.5%	0.98	99.4%	18.5%	0.98	99.6%	18.5%	0.98	99.5%
A*02	30.3%	29.4%	0.93	98.0%	32.6%	0.89	97.3%	30.4%	0.98	99.7%	30.3%	0.98	99.7%
A*02:01	28.8%	28.7%	0.97	98.8%	29.2%	0.98	99.2%	28.4%	0.99	99.3%	28.5%	0.99	99.4%
A*02:05	1.3%	0.6%	0.38	99.1%	1.4%	0.96	99.9%	1.4%	0.94	99.9%	1.3%	0.95	99.9%
A*02:06	0.1%	0.1%	0.02	99.8%	2.0%	0.00	97.9%	0.4%	0.55	99.7%	0.1%	1.00	100.0%
A*03	13.8%	13.9%	0.99	99.9%	14.9%	0.96	98.6%	13.8%	1.00	99.9%	13.8%	1.00	99.9%
A*03:01	13.8%	13.9%	0.99	99.9%	15.0%	0.96	98.6%	13.7%	0.99	99.8%	13.7%	0.99	99.9%
A*11	5.9%	5.8%	0.99	99.9%	5.8%	0.98	99.9%	5.9%	1.00	100.0%	5.9%	1.00	100.0%
A*11:01	5.9%	5.8%	0.99	99.9%	5.8%	0.98	99.9%	5.9%	1.00	100.0%	5.9%	1.00	100.0%
A*23	1.6%	3.8%	0.43	97.8%	1.6%	0.91	99.7%	1.7%	0.97	99.9%	1.7%	0.97	99.9%
A*23:01	1.6%	3.8%	0.43	97.8%	1.6%	0.91	99.7%	1.7%	0.97	99.9%	1.7%	0.97	99.9%
A*24	7.7%	7.9%	0.95	99.4%	7.0%	0.89	98.7%	7.7%	0.97	99.7%	7.7%	0.96	99.7%
A*24:02	7.5%	7.6%	0.93	99.5%	6.8%	0.88	98.7%	7.4%	0.95	99.4%	7.4%	0.94	99.4%
A*24:03	0.1%	0.0%	0.00	99.9%	0.0%	0.00	99.9%	0.1%	0.00	99.7%	0.1%	0.00	99.8%
A*25	1.5%	3.2%	0.43	97.9%	3.5%	0.35	97.5%	1.6%	0.68	99.3%	1.7%	0.68	99.3%
A*25:01	1.5%	3.2%	0.43	97.9%	3.5%	0.35	97.5%	1.6%	0.68	99.3%	1.7%	0.68	99.3%
A*26	2.4%	1.4%	0.52	98.0%	0.8%	0.53	97.7%	2.2%	0.81	99.4%	2.2%	0.81	99.4%
A*26:01	2.2%	1.4%	0.48	98.1%	0.8%	0.47	97.8%	2.2%	0.73	99.2%	2.1%	0.73	99.2%
A*26:08	0.2%	NA	NA	NA	NA	NA	NA	0.1%	0.02	99.8%	0.0%	0.29	99.8%

A*29	4.6%	3.9%	0.86	99.3%	4.5%	0.98	99.9%	4.6%	0.98	99.9%	4.6%	0.98	99.9%
A*29:02	4.6%	3.9%	0.86	99.3%	4.5%	0.98	99.9%	4.6%	0.98	99.9%	4.6%	0.98	99.9%
A*30	2.0%	0.2%	0.00	97.8%	0.1%	0.00	97.9%	2.0%	0.97	99.9%	2.0%	0.97	99.9%
A*30:01	1.4%	0.1%	0.00	98.5%	0.1%	0.00	98.5%	1.4%	1.00	100.0%	1.4%	1.00	100.0%
A*30:02	0.5%	0.1%	0.00	99.4%	0.0%	0.00	99.5%	0.4%	0.89	99.9%	0.4%	0.89	99.9%
A*30:04	0.1%	NA	NA	NA	NA	NA	NA	0.1%	1.00	100.0%	0.1%	1.00	100.0%
A*31	2.2%	2.7%	0.81	99.5%	2.7%	0.83	99.6%	2.3%	1.00	100.0%	2.2%	1.00	100.0%
A*31:01	2.2%	2.7%	0.81	99.5%	2.7%	0.83	99.6%	2.2%	1.00	100.0%	2.2%	1.00	100.0%
A*32	4.2%	4.8%	0.87	99.4%	4.3%	0.97	99.9%	4.1%	0.97	99.8%	4.2%	0.97	99.9%
A*32:01	4.2%	4.8%	0.87	99.4%	4.3%	0.97	99.9%	4.1%	0.97	99.9%	4.2%	0.97	99.9%
A*33	0.5%	NA	NA	NA	NA	NA	NA	0.5%	1.00	100.0%	0.5%	1.00	100.0%
A*33:03	0.1%	NA	NA	NA	NA	NA	NA	0.1%	0.50	99.9%	0.1%	0.50	99.9%
A*66	0.3%	NA	NA	NA	NA	NA	NA	0.3%	1.00	100.0%	0.3%	1.00	100.0%
A*66:01	0.3%	NA	NA	NA	NA	NA	NA	0.3%	1.00	100.0%	0.3%	1.00	100.0%
A*68	4.3%	4.4%	0.96	99.7%	3.9%	0.87	99.4%	4.3%	0.97	99.9%	4.3%	0.96	99.8%
A*68:01	3.9%	4.5%	0.87	99.3%	3.9%	0.96	99.8%	3.9%	0.97	99.9%	3.9%	0.96	99.8%
A*68:02	0.4%	NA	NA	NA	NA	NA	NA	0.4%	1.00	100.0%	0.4%	1.00	100.0%
C*01	3.6%	3.5%	0.78	99.2%	2.8%	0.70	98.9%	3.6%	0.90	99.6%	3.5%	0.92	99.7%
C*01:02	3.5%	3.5%	0.80	99.3%	2.8%	0.71	99.0%	3.6%	0.91	99.7%	3.5%	0.93	99.8%
C*02	3.4%	3.2%	0.76	99.4%	3.2%	0.76	99.4%	3.3%	0.78	99.5%	3.4%	0.80	99.6%
C*02:02	3.4%	3.2%	0.76	99.4%	3.2%	0.76	99.4%	3.3%	0.78	99.5%	3.4%	0.80	99.6%
C*03	15.7%	15.3%	0.91	98.6%	15.6%	0.95	99.4%	15.6%	0.94	99.3%	15.6%	0.94	99.3%
C*03:03	5.7%	5.9%	0.77	98.5%	5.9%	0.77	98.7%	5.8%	0.87	99.1%	5.9%	0.86	99.0%
C*03:04	9.0%	8.5%	0.86	98.6%	8.9%	0.85	98.7%	8.7%	0.89	98.8%	8.7%	0.90	98.9%
C*04	7.1%	7.2%	0.97	99.8%	7.2%	0.98	99.8%	7.2%	0.98	99.9%	7.2%	0.98	99.9%
C*04:01	7.1%	7.2%	0.97	99.8%	7.2%	0.98	99.8%	7.2%	0.98	99.9%	7.2%	0.98	99.9%
C*05	11.0%	10.1%	0.83	98.3%	12.8%	0.80	97.9%	11.3%	0.94	99.5%	11.3%	0.94	99.4%
C*05:01	11.0%	10.1%	0.83	98.3%	12.8%	0.80	97.9%	11.3%	0.94	99.5%	11.3%	0.94	99.4%
C*06	10.5%	10.7%	0.95	99.2%	10.7%	0.95	99.4%	10.5%	0.97	99.6%	10.4%	0.96	99.7%
C*06:02	10.5%	10.7%	0.95	99.2%	10.5%	0.96	99.6%	10.5%	0.97	99.6%	10.4%	0.96	99.7%
C*07	33.7%	36.3%	0.82	95.4%	33.9%	0.92	98.0%	33.5%	0.94	98.5%	33.5%	0.94	98.7%
C*07:01	16.8%	19.6%	0.78	96.1%	16.9%	0.96	99.3%	17.1%	0.96	99.2%	17.0%	0.96	99.5%
C*07:02	14.7%	14.7%	0.96	99.5%	16.0%	0.86	98.2%	14.7%	0.96	99.6%	14.7%	0.96	99.6%

C*07:04	2.0%	2.2%	0.84	99.5%	1.1%	0.10	97.9%	1.9%	0.89	99.8%	1.9%	0.91	99.8%
C*08	4.2%	2.9%	0.49	97.5%	2.5%	0.50	97.9%	4.0%	0.89	99.5%	4.0%	0.89	99.5%
C*08:02	4.2%	2.4%	0.50	98.0%	2.5%	0.50	97.9%	4.0%	0.89	99.5%	4.0%	0.89	99.5%
C*12	3.3%	4.0%	0.68	98.5%	3.5%	0.88	99.4%	3.4%	0.88	99.6%	3.4%	0.90	99.6%
C*12:02	0.6%	0.4%	0.70	99.8%	0.4%	0.70	99.8%	0.6%	1.00	100.0%	0.6%	1.00	100.0%
C*12:03	2.6%	3.4%	0.66	98.7%	3.0%	0.82	99.3%	2.6%	0.83	99.5%	2.7%	0.87	99.7%
C*14	0.7%	1.1%	0.39	99.2%	1.4%	0.37	99.1%	0.7%	0.85	99.9%	0.7%	0.85	99.9%
C*14:02	0.7%	1.1%	0.39	99.2%	1.4%	0.37	99.1%	0.7%	0.85	99.9%	0.7%	0.85	99.9%
C*15	1.8%	1.7%	0.84	99.7%	2.1%	0.81	99.6%	1.9%	0.91	99.8%	1.8%	0.94	99.9%
C*15:02	1.7%	1.7%	0.83	99.7%	2.1%	0.77	99.5%	1.6%	0.83	99.6%	1.7%	0.88	99.7%
C*15:05	0.1%	NA	NA	NA	NA	NA	NA	0.2%	0.17	99.8%	0.1%	0.30	99.9%
C*16	4.3%	4.2%	0.88	99.5%	4.1%	0.87	99.4%	4.4%	0.93	99.7%	4.4%	0.93	99.7%
C*16:01	4.2%	4.2%	0.92	99.7%	4.1%	0.90	99.6%	4.1%	0.93	99.7%	4.2%	0.93	99.7%
C*16:02	0.2%	NA	NA	NA	NA	NA	NA	0.2%	1.00	100.0%	0.2%	1.00	100.0%
C*17	0.6%	0.1%	0.17	99.5%	0.1%	0.09	99.4%	0.6%	0.93	100.0%	0.6%	1.00	100.0%
C*17:01	0.6%	0.1%	0.17	99.5%	0.1%	0.09	99.4%	0.6%	0.93	100.0%	0.6%	1.00	100.0%
B*07	13.7%	15.0%	0.89	97.8%	13.6%	0.97	99.4%	13.7%	0.97	99.5%	13.8%	0.98	99.7%
B*07:02	13.5%	14.9%	0.89	97.9%	13.5%	0.97	99.5%	13.5%	0.97	99.5%	13.5%	0.98	99.7%
B*08	13.9%	13.8%	0.95	98.7%	14.0%	0.95	99.0%	13.7%	0.96	99.1%	13.5%	0.97	99.4%
B*08:01	13.9%	13.8%	0.95	98.7%	14.2%	0.95	98.8%	13.7%	0.96	99.1%	13.5%	0.97	99.4%
B*13	1.9%	2.0%	0.94	99.7%	2.0%	0.97	99.9%	2.0%	0.96	99.9%	2.0%	0.97	99.9%
B*13:02	1.9%	2.0%	0.94	99.7%	2.0%	0.97	99.9%	2.0%	0.96	99.9%	2.0%	0.97	99.9%
B*14	3.9%	2.6%	0.52	98.0%	2.6%	0.50	98.0%	4.0%	0.98	99.9%	3.9%	0.97	99.9%
B*14:01	1.6%	0.2%	0.01	98.2%	0.3%	0.00	98.1%	1.6%	1.00	100.0%	1.6%	0.97	99.9%
B*14:02	2.3%	2.3%	0.95	99.9%	2.3%	0.95	99.9%	2.4%	0.97	99.9%	2.3%	0.95	99.9%
B*15	8.1%	7.4%	0.80	98.1%	8.9%	0.71	97.1%	8.1%	0.95	99.4%	8.1%	0.97	99.7%
B*15:01	7.2%	7.2%	0.82	98.5%	7.0%	0.94	99.4%	6.8%	0.93	99.2%	6.8%	0.96	99.5%
B*15:03	0.2%	NA	NA	NA	NA	NA	NA	0.1%	0.65	99.9%	0.2%	0.47	99.9%
B*15:16	0.1%	NA	NA	NA	NA	NA	NA	0.1%	1.00	100.0%	0.1%	0.99	100.0%
B*15:17	0.1%	NA	NA	NA	NA	NA	NA	0.1%	1.00	100.0%	0.1%	1.00	100.0%
B*15:18	0.4%	NA	NA	NA	NA	NA	NA	0.3%	0.85	99.9%	0.3%	1.00	99.9%
B*18	3.1%	2.9%	0.53	98.1%	3.4%	0.96	99.7%	3.3%	0.95	99.7%	3.2%	0.97	99.8%
B*18:01	3.1%	2.9%	0.53	98.1%	3.4%	0.96	99.7%	3.3%	0.95	99.7%	3.2%	0.97	99.8%

B*27	4.2%	6.0%	0.60	97.4%	4.4%	0.96	99.8%	4.2%	0.95	99.7%	4.3%	0.97	99.9%
B*27:05	4.2%	6.0%	0.60	97.4%	4.4%	0.96	99.8%	4.1%	0.94	99.6%	4.2%	0.97	99.8%
B*35	5.5%	5.5%	0.81	98.7%	5.0%	0.81	98.7%	5.5%	0.96	99.5%	5.5%	0.98	99.8%
B*35:01	3.0%	3.5%	0.70	98.8%	3.6%	0.79	99.2%	3.1%	0.87	99.4%	3.1%	0.90	99.5%
B*35:03	0.5%	0.6%	0.35	99.4%	0.1%	0.00	99.3%	0.6%	0.54	99.6%	0.5%	0.83	99.8%
B*35:08	0.3%	NA	NA	NA	NA	NA	NA	0.3%	0.67	99.7%	0.3%	0.64	99.8%
B*37	1.8%	2.0%	0.87	99.5%	1.8%	0.90	99.8%	1.8%	0.91	99.8%	1.8%	0.94	99.9%
B*37:01	1.8%	2.0%	0.87	99.5%	1.8%	0.90	99.8%	1.8%	0.91	99.8%	1.8%	0.94	99.9%
B*38	0.6%	0.2%	0.00	99.1%	0.5%	0.27	99.1%	0.6%	0.60	99.6%	0.6%	0.78	99.7%
B*38:01	0.6%	0.3%	0.00	99.1%	0.2%	0.16	99.2%	0.5%	0.65	99.6%	0.6%	0.77	99.8%
B*39	1.5%	0.8%	0.45	99.1%	0.8%	0.55	99.3%	1.5%	0.84	99.5%	1.6%	0.87	99.7%
B*39:01	0.8%	NA	NA	NA	NA	NA	NA	0.8%	0.81	99.7%	0.8%	0.85	99.8%
B*39:06	0.6%	0.7%	0.96	99.9%	0.8%	0.78	99.8%	0.6%	0.88	99.8%	0.6%	0.96	99.9%
B*40	6.9%	7.1%	0.95	99.4%	6.9%	0.96	99.7%	7.0%	0.96	99.7%	7.0%	0.96	99.7%
B*40:01	6.2%	6.3%	0.94	99.5%	6.2%	0.94	99.6%	6.3%	0.95	99.6%	6.3%	0.95	99.7%
B*40:02	0.6%	0.8%	0.86	99.8%	0.7%	0.92	99.9%	0.7%	0.92	99.9%	0.6%	0.89	99.9%
B*41	0.9%	0.1%	0.06	99.2%	0.1%	0.06	99.2%	0.8%	0.89	99.9%	0.8%	0.94	99.9%
B*41:01	0.5%	0.1%	0.13	99.6%	0.1%	0.13	99.6%	0.4%	0.73	99.9%	0.4%	0.87	99.9%
B*41:02	0.2%	NA	NA	NA	NA	NA	NA	0.2%	0.75	99.9%	0.2%	1.00	100.0%
B*44	17.9%	19.0%	0.92	97.8%	19.0%	0.92	98.2%	17.5%	0.96	99.3%	17.8%	0.98	99.7%
B*44:02	11.7%	12.8%	0.91	98.1%	12.5%	0.91	98.7%	11.4%	0.96	99.3%	11.7%	0.97	99.6%
B*44:03	5.6%	5.4%	0.93	99.5%	5.4%	0.89	99.4%	5.5%	0.94	99.7%	5.5%	0.95	99.7%
B*45	1.1%	NA	NA	NA	NA	NA	NA	1.0%	0.96	99.9%	1.1%	1.00	100.0%
B*45:01	1.1%	NA	NA	NA	NA	NA	NA	1.0%	0.96	99.9%	1.1%	1.00	100.0%
B*47	0.5%	NA	NA	NA	NA	NA	NA	0.5%	0.94	99.9%	0.5%	1.00	100.0%
B*47:01	0.5%	NA	NA	NA	NA	NA	NA	0.5%	0.94	99.9%	0.5%	1.00	100.0%
B*49	1.1%	1.1%	0.80	99.8%	2.9%	0.31	98.0%	1.1%	0.95	99.9%	1.1%	0.95	99.9%
B*49:01	1.1%	1.1%	0.80	99.8%	2.9%	0.31	98.0%	1.1%	0.95	99.9%	1.1%	0.95	99.9%
B*50	0.9%	1.8%	0.41	98.9%	0.4%	0.00	98.7%	0.9%	0.85	99.8%	0.8%	0.87	99.9%
B*50:01	0.9%	1.8%	0.41	98.9%	0.4%	0.00	98.7%	0.8%	0.85	99.8%	0.8%	0.87	99.9%
B*51	4.3%	4.8%	0.64	97.9%	5.0%	0.81	98.9%	4.2%	0.91	99.4%	4.2%	0.93	99.6%
B*51:01	3.7%	4.3%	0.64	98.1%	4.4%	0.79	98.8%	3.6%	0.91	99.5%	3.5%	0.92	99.5%
B*52	0.6%	0.7%	0.87	99.8%	1.2%	0.71	99.2%	0.7%	0.88	99.8%	0.8%	0.85	99.8%

B*52:01	0.6%	0.6%	0.87	99.8%	1.2%	0.71	99.2%	0.7%	0.88	99.8%	0.8%	0.85	99.8%
B*53	0.2%	NA	NA	NA	NA	NA	NA	0.2%	1.00	100.0%	0.2%	1.00	100.0%
B*53:01	0.2%	NA	NA	NA	NA	NA	NA	0.2%	1.00	100.0%	0.2%	1.00	100.0%
B*55	2.1%	2.3%	0.97	99.8%	2.4%	0.92	99.4%	2.2%	0.98	99.9%	2.2%	0.99	99.9%
B*55:01	2.1%	2.2%	0.98	99.8%	2.3%	0.92	99.5%	2.2%	0.98	99.9%	2.2%	0.99	99.9%
B*56	0.3%	1.2%	0.28	99.1%	0.4%	0.81	99.9%	0.3%	0.88	99.9%	0.3%	0.91	99.9%
B*56:01	0.3%	1.2%	0.28	99.1%	0.4%	0.81	99.9%	0.3%	0.84	99.9%	0.3%	0.88	99.9%
B*57	4.5%	4.4%	0.94	99.7%	5.1%	0.83	99.2%	4.5%	0.94	99.7%	4.4%	0.97	99.9%
B*57:01	4.5%	4.4%	0.94	99.7%	5.0%	0.83	99.2%	4.5%	0.94	99.7%	4.3%	0.97	99.8%
B*58	0.5%	0.0%	0.59	99.5%	0.4%	0.78	99.9%	0.5%	1.00	100.0%	0.5%	1.00	100.0%
B*58:01	0.5%	0.0%	0.60	99.5%	0.4%	0.78	99.9%	0.5%	1.00	100.0%	0.5%	1.00	100.0%
DRB1*01	11.5%	11.6%	0.82	97.9%	11.2%	0.83	98.1%	11.4%	0.98	99.8%	11.5%	1.00	100.0%
DRB1*01:01	9.1%	11.6%	0.73	97.0%	11.2%	0.79	97.7%	9.5%	0.91	98.8%	9.2%	0.98	99.6%
DRB1*01:02	0.7%	NA	NA	NA	NA	NA	NA	0.7%	1.00	100.0%	0.7%	1.00	100.0%
DRB1*01:03	1.6%	NA	NA	NA	NA	NA	NA	1.2%	0.65	99.1%	1.6%	0.90	99.7%
DRB1*03	13.1%	12.8%	0.85	96.4%	13.2%	0.87	97.3%	13.0%	0.97	99.5%	13.1%	0.96	99.4%
DRB1*03:01	13.0%	12.6%	0.84	96.4%	13.1%	0.87	97.3%	12.8%	0.97	99.5%	12.9%	0.96	99.5%
DRB1*04	20.7%	21.7%	0.88	96.1%	22.4%	0.90	97.7%	20.8%	0.95	98.8%	20.9%	0.98	99.6%
DRB1*04:01	11.9%	12.2%	0.66	94.3%	13.3%	0.77	96.7%	12.6%	0.81	97.4%	12.4%	0.87	98.5%
DRB1*04:02	0.3%	0.1%	0.01	99.7%	0.0%	0.00	99.7%	0.2%	0.41	99.7%	0.4%	0.49	99.8%
DRB1*04:03	1.8%	1.3%	0.03	97.2%	0.4%	0.02	98.0%	0.5%	0.03	98.0%	0.6%	0.14	98.2%
DRB1*04:04	5.1%	5.9%	0.62	96.6%	6.5%	0.64	97.3%	4.6%	0.71	98.1%	4.8%	0.81	98.8%
DRB1*04:05	0.5%	0.1%	0.00	99.4%	0.0%	0.00	99.5%	0.7%	0.59	99.6%	0.5%	0.88	99.9%
DRB1*04:07	0.6%	1.5%	0.22	98.7%	1.5%	0.26	98.8%	1.3%	0.36	99.1%	1.3%	0.37	99.2%
DRB1*07	14.7%	16.5%	0.84	96.8%	15.2%	0.92	98.9%	14.7%	0.97	99.5%	14.6%	0.98	99.7%
DRB1*07:01	14.7%	16.5%	0.84	96.8%	15.2%	0.92	98.9%	14.7%	0.97	99.5%	14.6%	0.98	99.7%
DRB1*08	2.5%	1.8%	0.73	99.3%	1.8%	0.72	99.2%	3.0%	0.79	99.2%	2.5%	0.98	99.9%
DRB1*08:01	1.5%	1.3%	0.72	99.5%	1.3%	0.72	99.5%	1.6%	0.86	99.7%	1.5%	0.92	99.8%
DRB1*08:03	0.1%	NA	NA	NA	NA	NA	NA	0.1%	0.50	99.9%	0.1%	0.25	99.9%
DRB1*08:04	0.3%	NA	NA	NA	NA	NA	NA	0.4%	0.01	99.4%	0.2%	0.68	99.9%
DRB1*09	1.6%	NA	NA	NA	NA	NA	NA	1.6%	0.74	99.4%	1.6%	1.00	100.0%
DRB1*09:01	1.6%	NA	NA	NA	NA	NA	NA	1.6%	0.74	99.4%	1.6%	1.00	100.0%
DRB1*10	0.8%	0.1%	0.00	99.1%	0.1%	0.00	99.2%	0.8%	1.00	100.0%	0.8%	1.00	100.0%

DRB1*10:01	0.8%	0.2%	0.00	99.1%	0.1%	0.00	99.2%	0.8%	1.00	100.0%	0.8%	1.00	100.0%
DRB1*11	6.9%	8.0%	0.74	97.0%	7.6%	0.82	98.2%	7.4%	0.87	98.8%	6.7%	0.94	99.5%
DRB1*11:01	2.9%	5.3%	0.55	96.9%	5.0%	0.50	97.2%	3.5%	0.71	98.5%	2.8%	0.89	99.4%
DRB1*11:02	0.5%	NA	NA	NA	NA	NA	NA	0.4%	0.77	99.8%	0.6%	0.70	99.8%
DRB1*11:03	0.4%	0.3%	0.21	99.5%	0.2%	0.28	99.6%	0.4%	0.58	99.7%	0.4%	0.66	99.8%
DRB1*11:04	0.6%	0.2%	0.00	99.1%	0.1%	0.00	99.3%	0.7%	0.53	99.5%	0.6%	0.56	99.5%
DRB1*12	1.9%	3.6%	0.53	98.2%	1.9%	0.97	99.9%	1.4%	0.81	99.4%	1.9%	1.00	100.0%
DRB1*12:01	1.6%	3.5%	0.48	98.1%	1.8%	0.90	99.8%	1.4%	0.84	99.6%	1.7%	0.93	99.8%
DRB1*12:02	0.1%	NA	NA	NA	NA	NA	NA	0.0%	0.00	99.9%	0.0%	0.00	99.9%
DRB1*13	10.1%	7.4%	0.65	95.6%	10.0%	0.94	98.4%	9.5%	0.88	98.6%	10.0%	0.99	99.8%
DRB1*13:01	4.6%	3.1%	0.56	97.1%	4.7%	0.90	98.6%	4.3%	0.83	99.1%	4.7%	0.98	99.9%
DRB1*13:02	4.5%	3.7%	0.46	97.1%	4.5%	0.97	99.7%	4.4%	0.97	99.9%	4.4%	0.99	99.9%
DRB1*13:03	0.7%	0.7%	1.00	100.0%	0.7%	1.00	100.0%	0.5%	0.60	99.7%	0.7%	1.00	100.0%
DRB1*14	2.5%	2.8%	0.43	97.7%	2.6%	0.86	99.3%	2.7%	0.65	98.9%	2.6%	0.93	99.8%
DRB1*14:01	2.5%	2.7%	0.45	97.9%	2.3%	0.84	99.5%	1.9%	0.74	99.1%	2.3%	0.90	99.6%
DRB1*15	13.2%	13.3%	0.92	97.7%	13.4%	0.89	97.6%	13.3%	0.99	99.8%	13.3%	0.99	99.9%
DRB1*15:01	12.9%	13.2%	0.94	97.9%	13.3%	0.92	97.9%	12.7%	0.97	99.6%	12.7%	0.98	99.7%
DRB1*15:02	0.3%	0.1%	0.00	99.6%	0.3%	0.00	99.4%	0.5%	0.50	99.7%	0.5%	0.51	99.7%
DRB1*16	0.5%	0.4%	0.61	99.5%	0.8%	0.62	99.5%	0.5%	0.89	99.9%	0.6%	0.97	100.0%
DRB1*16:01	0.5%	0.4%	0.65	99.5%	0.8%	0.59	99.5%	0.5%	0.86	99.9%	0.5%	1.00	100.0%
DQB1*02	23.4%	21.6%	0.65	92.2%	22.3%	0.64	92.0%	23.9%	0.95	98.9%	23.7%	0.95	98.9%
DQB1*02:01	13.1%	12.8%	0.71	95.1%	12.0%	0.73	96.1%	13.4%	0.95	99.0%	13.2%	0.94	99.1%
DQB1*02:02	10.2%	8.7%	0.60	95.5%	10.8%	0.50	93.2%	10.5%	0.95	99.4%	10.5%	0.95	99.5%
DQB1*03	36.6%	36.5%	0.75	92.2%	36.7%	0.69	90.6%	36.3%	0.97	99.3%	36.3%	0.97	99.3%
DQB1*03:01	20.2%	21.1%	0.67	92.2%	21.9%	0.64	91.5%	20.0%	0.94	98.9%	20.0%	0.95	99.2%
DQB1*03:02	9.1%	9.5%	0.74	96.2%	9.4%	0.77	97.5%	9.2%	0.95	99.6%	9.2%	0.95	99.6%
DQB1*03:03	5.8%	4.3%	0.65	97.7%	4.0%	0.65	98.0%	5.7%	0.91	99.4%	5.7%	0.97	99.8%
DQB1*03:04	0.1%	NA	NA	NA	NA	NA	NA	0.0%	0.00	99.9%	0.0%	0.00	99.9%
DQB1*04	2.4%	1.8%	0.79	99.4%	1.8%	0.80	99.4%	2.4%	1.00	100.0%	2.4%	1.00	100.0%
DQB1*04:02	2.4%	1.8%	0.79	99.4%	1.8%	0.80	99.4%	2.4%	1.00	100.0%	2.4%	1.00	100.0%
DQB1*05	15.2%	13.1%	0.62	93.0%	13.7%	0.62	93.8%	15.1%	0.95	99.1%	15.4%	0.98	99.7%
DQB1*05:01	12.0%	10.7%	0.68	95.1%	9.6%	0.77	96.8%	12.0%	1.00	99.9%	12.0%	1.00	100.0%
DQB1*05:02	0.7%	0.5%	0.59	99.3%	1.0%	0.59	99.3%	0.7%	0.90	99.8%	0.7%	0.79	99.8%

DQB1*05:03	2.6%	1.9%	0.43	98.2%	2.9%	0.29	97.6%	2.4%	0.74	99.1%	2.7%	0.93	99.8%
DQB1*06	22.4%	27.5%	0.63	90.4%	25.2%	0.55	90.4%	22.3%	0.96	98.9%	22.2%	0.98	99.5%
DQB1*06:01	0.4%	0.0%	nan	99.6%	0.0%	nan	99.6%	0.5%	0.89	99.9%	0.5%	0.89	99.9%
DQB1*06:02	12.6%	19.6%	0.55	90.4%	17.0%	0.62	92.7%	12.8%	0.91	98.3%	12.2%	0.95	99.0%
DQB1*06:03	5.2%	4.0%	0.56	96.7%	4.9%	0.55	96.5%	4.8%	0.80	98.6%	5.3%	0.92	99.2%
DQB1*06:04	3.2%	2.8%	0.72	98.9%	2.9%	0.77	99.1%	3.2%	0.98	99.9%	3.2%	1.00	99.9%
DQB1*06:09	1.0%	0.9%	0.87	99.9%	0.0%	nan	99.0%	1.0%	0.99	100.0%	1.0%	1.00	100.0%

Table S2: Comparison of imputation accuracy of four-digit classical HLA alleles between SNP2HLA and HLA*IMP. For SNP2HLA, we used the large T1DGC reference panel and the high-density Illumina Immunochip for HLA imputations, and measured their accuracy against gold-standard HLA types in 918 individuals of the British 1958 Birth Cohort (see Table 2). For HLA*IMP, we copied the accuracies from a published cross-validation experiment using the British 1958 Birth Cohort (2/3 training data, 1/3 validation data). Although this does not represent a formal head-to-head comparison, the results illustrate that both methods achieve comparable imputation quality and highlight a consistent trend that imputation of *HLA-B* and *HLA-DRB1* loci is more challenging.

	**HLA*IMP**	**SNP2HLA**
HLA-A	97%	98%
HLA-B	96%	97%
HLA-C	97%	97%
HLA-DQB1	98%	98%
HLA-DRB1	92%	93%

Table S3: Imputation performance (dosage r^2 per position) and accuracy (genotype concordance) is shown for individual HLA amino acid positions in 918 individuals from the British 1958 Birth Cohort. R^2 per position was calculated using the modified Pearson correlation formula for multi-allelic amino acid positions (see Methods). Performance is shown for four imputation scenarios, each using a large (T1DGC) or small (HapMap CEU) reference panel, and a high-density (Illumina Immunochip) or low-density (Affymetrix 500K) SNP chip. Accuracy was based on the same set of variants, allowing a direct and fair comparison between reference panels and SNP chips.

			HapMap-CEPH (n=90)				T1DGC (n=5,225)			
			Affymetrix 500K		Illumina Immunochip		Affymetrix 500K		Illumina Immunochip	
**locus**	**pos**	**# alleles**	***r2***	**accuracy**	***r2***	**accuracy**	***r2***	**accuracy**	***r2***	**accuracy**
A	-15	2	0.45	95.4%	0.49	97.3%	0.99	99.4%	0.99	99.4%
A	-11	2	0.49	99.4%	0.49	99.7%	0.98	99.6%	0.99	99.8%
A	9	4	0.92	96.2%	0.95	96.4%	0.97	99.0%	0.98	99.3%
A	17	2	0.02	96.0%	0.96	99.3%	0.97	99.9%	0.97	99.9%
A	43	2	0.38	98.4%	0.92	99.8%	0.96	99.9%	0.96	99.9%
A	44	2	0.95	98.1%	0.98	99.2%	0.98	99.3%	0.98	99.3%
A	56	2	0.47	95.9%	0.95	99.2%	0.99	99.9%	0.99	99.9%
A	62	5	0.89	94.6%	0.95	96.6%	0.98	99.3%	0.99	99.3%
A	63	3	0.91	97.8%	0.94	98.6%	0.98	99.6%	0.98	99.7%
A	65	2	0.78	97.4%	0.92	97.8%	0.98	99.7%	0.98	99.7%
A	66	2	0.88	95.1%	0.96	96.9%	0.99	99.5%	0.99	99.5%
A	67	2	0.95	98.1%	0.98	99.2%	0.98	99.3%	0.98	99.3%
A	70	2	0.88	95.7%	0.91	95.1%	0.99	99.5%	0.99	99.5%
A	73	2	1.00	100.0%	1.00	100.0%	1.00	100.0%	1.00	100.0%
A	74	2	0.93	97.2%	0.96	98.9%	0.98	99.4%	0.98	99.4%
A	76	3	0.83	92.7%	0.87	92.8%	0.96	98.5%	0.96	98.5%
A	77	3	0.83	93.0%	0.87	93.2%	0.96	98.7%	0.96	98.7%
A	79	2	0.74	93.8%	0.80	94.5%	0.93	98.6%	0.93	98.6%
A	80	2	0.74	93.8%	0.80	94.5%	0.93	98.6%	0.93	98.6%
A	81	2	0.74	93.8%	0.80	94.5%	0.93	98.6%	0.93	98.6%
A	82	2	0.74	93.8%	0.80	94.5%	0.93	98.6%	0.93	98.6%
A	83	2	0.74	93.8%	0.80	94.5%	0.93	98.6%	0.93	98.6%

A	90	2	0.93	97.3%	0.98	99.0%	0.99	99.3%	0.99	99.3%
A	95	3	0.89	95.2%	0.95	97.0%	0.99	99.5%	0.99	99.5%
A	97	3	0.90	94.6%	0.97	97.3%	0.99	99.4%	0.99	99.3%
A	99	2	0.78	97.4%	0.92	97.8%	0.98	99.7%	0.98	99.7%
A	105	2	0.90	95.7%	0.96	98.3%	0.99	99.3%	0.98	99.3%
A	107	2	0.94	97.1%	0.96	98.8%	0.98	99.5%	0.98	99.5%
A	109	2	0.89	99.3%	0.99	99.9%	0.98	99.8%	0.99	99.9%
A	114	4	0.87	93.4%	0.97	96.9%	0.99	99.1%	0.99	99.2%
A	116	3	0.85	93.9%	0.96	96.7%	0.99	99.3%	0.99	99.3%
A	127	2	0.88	94.9%	0.97	97.3%	0.99	99.5%	0.99	99.5%
A	142	2	0.94	97.1%	0.98	99.3%	0.98	99.6%	0.98	99.5%
A	144	2	0.95	98.3%	0.97	98.8%	0.97	99.5%	0.98	99.6%
A	145	2	0.94	97.1%	0.98	99.2%	0.98	99.6%	0.98	99.5%
A	149	2	0.88	99.0%	0.96	99.7%	0.96	99.6%	0.96	99.6%
A	150	2	0.95	97.9%	0.98	99.1%	0.98	99.2%	0.98	99.2%
A	151	2	0.96	98.8%	0.95	98.7%	0.98	99.7%	0.98	99.8%
A	152	5	0.90	94.5%	0.92	94.7%	0.99	98.9%	0.98	98.9%
A	156	4	0.95	96.8%	0.94	95.8%	0.97	98.4%	0.97	98.5%
A	158	2	0.95	97.8%	0.98	99.0%	0.98	99.2%	0.98	99.2%
A	161	2	0.99	99.8%	0.96	97.8%	1.00	99.9%	1.00	99.9%
A	163	2	0.93	97.1%	0.98	98.8%	0.99	99.2%	0.99	99.2%
A	166	2	0.86	95.2%	0.95	97.2%	0.98	99.1%	0.98	99.1%
A	167	2	0.86	95.2%	0.95	97.1%	0.98	99.1%	0.98	99.1%
A	184	2	0.95	97.4%	0.92	96.6%	0.99	99.6%	0.99	99.6%
A	193	2	0.95	97.5%	0.92	96.6%	0.99	99.6%	0.99	99.6%
A	194	2	0.95	97.5%	0.92	96.6%	0.99	99.6%	0.99	99.6%
A	207	2	0.95	97.4%	0.92	96.6%	0.99	99.6%	0.99	99.6%
A	245	2	0.95	99.4%	0.86	98.8%	0.96	99.7%	0.95	99.7%
A	246	2	0.94	98.5%	0.97	99.3%	0.98	99.5%	0.99	99.7%
A	253	2	0.95	97.7%	0.92	96.8%	0.99	99.7%	0.99	99.6%
A	276	2	0.45	95.4%	0.45	94.8%	0.99	99.3%	0.99	99.2%
A	282	2	0.38	97.0%	0.47	97.8%	0.97	99.5%	0.97	99.5%
A	283	2	0.24	97.7%	0.49	99.5%	0.91	99.7%	0.93	99.8%

A	294	2	0.47	97.6%	0.46	96.8%	0.99	99.6%	0.99	99.5%
A	297	2	0.44	99.2%	0.49	99.8%	0.96	99.6%	0.97	99.7%
A	298	2	0.49	99.5%	0.49	99.6%	0.98	99.6%	0.99	99.7%
A	299	2	0.47	98.4%	0.48	99.4%	0.98	99.4%	0.98	99.6%
A	307	2	0.49	99.5%	0.49	99.6%	0.98	99.6%	0.99	99.7%
A	311	2	0.38	97.0%	0.47	97.8%	0.97	99.5%	0.97	99.5%
A	321	2	0.45	95.4%	0.45	94.8%	0.99	99.3%	0.99	99.3%
A	334	2	0.47	98.4%	0.48	99.4%	0.98	99.4%	0.98	99.6%
B	-23	2	0.89	95.0%	0.92	96.5%	0.96	97.9%	0.98	99.2%
B	-21	2	0.89	95.0%	0.92	96.5%	0.96	97.9%	0.98	99.2%
B	-16	2	0.80	92.6%	0.94	97.6%	0.95	97.8%	0.97	99.0%
B	-11	2	0.86	93.8%	0.91	95.3%	0.95	97.3%	0.96	98.7%
B	-10	2	0.85	93.6%	0.88	94.9%	0.94	97.3%	0.96	98.8%
B	-8	2	0.86	93.8%	0.91	95.4%	0.95	97.3%	0.96	98.8%
B	9	3	0.82	92.0%	0.94	96.9%	0.96	97.7%	0.98	98.8%
B	11	2	0.84	94.2%	0.95	97.7%	0.96	98.6%	0.97	99.2%
B	12	2	0.86	94.4%	0.95	97.8%	0.96	98.7%	0.97	99.4%
B	24	3	0.83	91.7%	0.93	95.6%	0.96	97.7%	0.98	99.0%
B	30	2	0.58	97.4%	0.96	99.5%	0.95	99.6%	0.98	99.8%
B	32	2	0.85	94.0%	0.95	98.0%	0.96	98.2%	0.98	99.3%
B	41	2	0.93	96.8%	0.94	97.8%	0.96	98.4%	0.97	99.2%
B	45	4	0.89	93.9%	0.92	95.7%	0.95	97.1%	0.97	98.7%
B	46	2	0.88	97.3%	0.90	97.9%	0.94	98.6%	0.97	99.3%
B	62	2	0.84	98.6%	0.86	99.1%	0.94	99.5%	0.97	99.7%
B	63	2	0.83	92.8%	0.94	97.0%	0.95	97.5%	0.97	98.8%
B	65	2	0.79	98.1%	0.82	98.6%	0.94	99.4%	0.97	99.7%
B	66	2	0.79	98.1%	0.82	98.6%	0.94	99.4%	0.97	99.7%
B	67	5	0.83	90.6%	0.88	93.5%	0.95	96.8%	0.97	98.3%
B	69	2	0.84	94.0%	0.88	96.4%	0.98	98.8%	0.99	99.4%
B	70	4	0.83	93.8%	0.89	96.4%	0.97	98.6%	0.98	99.3%
B	71	2	0.84	94.0%	0.88	96.4%	0.98	98.8%	0.99	99.4%
B	74	2	0.87	93.2%	0.95	97.0%	0.96	97.5%	0.97	98.7%
B	77	3	0.78	90.8%	0.81	91.6%	0.94	96.8%	0.96	98.0%

B	80	3	0.80	91.2%	0.82	92.7%	0.94	96.8%	0.96	98.0%
B	81	2	0.80	92.1%	0.79	91.9%	0.95	97.1%	0.97	98.1%
B	82	2	0.82	92.1%	0.81	91.9%	0.94	97.0%	0.96	98.1%
B	83	2	0.82	92.2%	0.81	91.9%	0.94	97.0%	0.96	98.1%
B	94	2	0.88	95.6%	0.90	96.0%	0.95	97.6%	0.98	99.0%
B	95	3	0.77	89.4%	0.80	90.9%	0.95	97.0%	0.97	98.5%
B	97	6	0.73	86.7%	0.82	91.0%	0.96	97.1%	0.98	98.2%
B	99	2	0.51	98.8%	0.52	99.1%	0.92	99.6%	0.95	99.8%
B	103	2	0.59	92.1%	0.66	92.9%	0.96	98.7%	0.98	99.4%
B	113	2	0.79	91.8%	0.88	95.2%	0.97	98.4%	0.98	99.2%
B	114	3	0.65	85.0%	0.76	88.7%	0.95	96.9%	0.97	98.5%
B	116	5	0.71	85.3%	0.80	89.3%	0.95	96.7%	0.97	98.4%
B	131	2	0.77	91.3%	0.80	92.7%	0.96	97.7%	0.98	98.9%
B	143	2	0.94	99.3%	0.94	99.4%	0.95	99.4%	0.95	99.6%
B	145	2	0.94	99.6%	0.97	99.9%	0.96	99.9%	0.97	99.9%
B	147	2	0.94	99.3%	0.94	99.4%	0.95	99.4%	0.95	99.5%
B	152	2	0.82	92.5%	0.88	94.4%	0.97	98.2%	0.97	98.6%
B	156	4	0.74	87.1%	0.78	89.0%	0.95	96.4%	0.96	97.7%
B	158	2	0.31	96.9%	0.76	97.9%	0.94	99.5%	0.97	99.8%
B	163	3	0.74	86.8%	0.83	91.6%	0.96	97.5%	0.98	98.7%
B	167	2	0.87	96.1%	0.86	96.1%	0.96	98.6%	0.98	99.4%
B	171	2	0.73	94.5%	0.76	94.9%	0.95	98.9%	0.97	99.2%
B	177	2	0.76	91.2%	0.79	93.0%	0.96	97.7%	0.97	98.7%
B	178	2	0.78	94.1%	0.79	95.0%	0.97	98.8%	0.98	99.2%
B	180	2	0.76	91.2%	0.79	93.1%	0.96	97.7%	0.97	98.7%
B	194	2	0.72	95.5%	0.92	97.8%	0.95	98.7%	0.96	99.3%
B	199	2	0.91	97.1%	0.92	97.8%	0.96	98.6%	0.98	99.3%
B	282	2	0.84	93.5%	0.73	90.1%	0.96	98.0%	0.98	99.1%
B	305	2	0.84	93.5%	0.73	90.1%	0.96	98.1%	0.98	99.2%
B	325	2	0.80	91.2%	0.87	94.4%	0.97	98.3%	0.98	99.3%
C	-18	2	0.09	98.8%	0.09	98.8%	0.89	99.8%	0.96	99.9%
C	-17	2	0.82	93.9%	0.91	96.9%	0.96	98.6%	0.97	99.1%
C	-15	2	0.81	93.4%	0.94	98.3%	0.94	98.3%	0.95	98.8%

C	-9	2	0.84	94.4%	0.94	98.2%	0.96	98.6%	0.97	99.1%
C	-5	2	0.09	98.8%	0.09	98.8%	0.89	99.8%	0.96	99.9%
C	1	2	0.90	97.2%	0.92	97.7%	0.97	99.2%	0.97	99.4%
C	6	2	0.78	98.7%	0.70	97.9%	0.90	99.4%	0.92	99.5%
C	9	4	0.84	93.1%	0.92	96.7%	0.96	98.5%	0.97	99.0%
C	11	2	0.88	97.8%	0.97	99.5%	0.97	99.5%	0.98	99.6%
C	14	2	0.98	99.8%	0.98	99.8%	0.99	99.9%	0.99	99.9%
C	16	2	0.78	99.3%	0.78	99.3%	0.80	99.4%	0.82	99.6%
C	21	2	0.91	97.7%	0.91	98.2%	0.95	99.0%	0.96	99.5%
C	24	2	0.84	93.9%	0.90	96.1%	0.96	98.6%	0.97	99.1%
C	35	2	0.74	93.8%	0.96	99.3%	0.96	99.5%	0.97	99.6%
C	49	2	0.98	99.8%	0.98	99.8%	0.99	99.9%	0.99	99.9%
C	66	2	0.77	94.5%	0.94	98.6%	0.94	98.6%	0.96	99.2%
C	73	2	0.83	93.6%	0.94	98.3%	0.95	98.7%	0.96	99.0%
C	77	2	0.86	94.6%	0.84	95.4%	0.95	98.5%	0.96	99.0%
C	80	2	0.86	94.6%	0.84	95.3%	0.95	98.5%	0.96	99.0%
C	90	2	0.85	94.1%	0.94	97.7%	0.96	98.7%	0.97	99.1%
C	91	2	0.80	98.2%	0.80	98.5%	0.90	98.9%	0.89	98.8%
C	94	2	0.92	98.0%	0.93	98.7%	0.96	99.3%	0.97	99.6%
C	95	3	0.88	96.9%	0.80	95.1%	0.95	98.9%	0.96	99.2%
C	97	2	0.87	96.4%	0.91	97.9%	0.95	98.6%	0.95	98.9%
C	99	4	0.91	97.3%	0.87	96.7%	0.96	99.0%	0.96	99.1%
C	103	2	0.92	98.4%	0.94	99.0%	0.96	99.4%	0.96	99.5%
C	113	2	0.84	99.4%	0.81	99.4%	0.91	99.6%	0.93	99.7%
C	114	2	0.78	93.1%	0.94	98.2%	0.97	99.3%	0.97	99.4%
C	116	4	0.81	91.7%	0.86	94.1%	0.94	98.0%	0.95	98.3%
C	138	2	0.74	93.9%	0.97	99.5%	0.96	99.5%	0.96	99.5%
C	143	2	0.10	98.8%	0.09	98.9%	0.93	99.9%	1.00	100.0%
C	147	2	0.82	93.7%	0.97	98.9%	0.96	98.6%	0.97	98.9%
C	152	2	0.83	93.7%	0.93	97.5%	0.96	98.4%	0.97	98.9%
C	156	5	0.82	91.7%	0.89	95.4%	0.95	98.1%	0.96	98.5%
C	163	3	0.87	96.7%	0.90	97.9%	0.93	99.0%	0.94	99.2%
C	170	2	0.16	98.8%	0.09	98.9%	0.93	99.9%	1.00	100.0%

C	173	2	0.92	98.3%	0.97	99.6%	0.96	99.4%	0.96	99.5%
C	177	2	0.75	93.4%	0.83	96.3%	0.95	99.1%	0.96	99.2%
C	184	3	0.82	93.6%	0.94	97.9%	0.96	98.5%	0.97	99.0%
C	193	2	0.91	99.3%	0.90	99.2%	0.97	99.7%	0.97	99.7%
C	194	2	0.84	94.3%	0.94	97.9%	0.96	98.5%	0.97	99.0%
C	211	2	0.78	99.2%	0.77	99.3%	0.80	99.4%	0.82	99.6%
C	219	2	0.90	97.0%	0.97	98.9%	0.96	99.0%	0.97	99.1%
C	248	2	0.79	98.8%	0.71	98.0%	0.91	99.4%	0.93	99.6%
C	253	2	0.82	93.9%	0.97	98.9%	0.96	98.7%	0.97	99.0%
C	261	2	0.84	94.4%	0.94	97.9%	0.96	98.6%	0.97	99.0%
C	267	2	0.82	93.9%	0.97	98.9%	0.96	98.7%	0.97	99.0%
C	270	2	0.16	98.9%	0.09	98.8%	0.89	99.8%	0.96	99.9%
C	273	2	0.84	94.4%	0.94	98.0%	0.96	98.6%	0.97	99.0%
C	275	3	0.75	92.5%	0.94	98.4%	0.97	99.2%	0.97	99.3%
C	284	2	0.16	98.9%	0.09	98.8%	0.89	99.8%	0.96	99.9%
C	285	3	0.82	93.8%	0.94	98.0%	0.96	98.6%	0.97	98.9%
C	289	2	0.16	98.9%	0.09	98.8%	0.89	99.8%	0.96	99.9%
C	291	2	0.16	98.9%	0.09	98.8%	0.89	99.8%	0.96	99.9%
C	295	2	0.84	94.4%	0.94	98.0%	0.96	98.7%	0.96	99.0%
C	303	2	0.98	99.7%	0.98	99.8%	0.99	99.8%	0.99	99.9%
C	304	2	0.80	92.3%	0.96	98.4%	0.97	98.7%	0.97	98.9%
C	305	2	0.84	94.4%	0.94	98.1%	0.96	98.7%	0.96	99.0%
C	306	2	0.82	93.9%	0.97	98.8%	0.96	98.7%	0.97	99.0%
C	307	2	0.84	94.4%	0.94	98.0%	0.96	98.7%	0.96	98.9%
C	308	2	0.16	98.9%	0.09	98.8%	0.89	99.8%	0.96	99.9%
C	309	2	0.16	98.8%	0.09	98.8%	0.89	99.8%	0.96	99.9%
C	326	2	0.84	94.0%	0.94	97.4%	0.96	98.7%	0.96	98.9%
C	339	2	0.84	94.4%	0.94	98.0%	0.96	98.7%	0.96	98.9%
DQB1	-27	3	0.63	87.0%	0.64	88.5%	0.95	98.3%	0.97	99.1%
DQB1	-21	3	0.63	82.8%	0.61	82.9%	0.98	98.7%	0.97	98.5%
DQB1	-18	3	0.62	81.0%	0.61	82.2%	0.96	98.0%	0.97	98.3%
DQB1	-17	3	0.61	97.0%	0.61	96.8%	0.94	99.5%	0.94	99.5%
DQB1	-10	3	0.67	87.8%	0.65	87.2%	0.96	98.4%	0.95	98.5%

DQB1	-9	3	0.63	87.0%	0.64	88.5%	0.95	98.3%	0.97	99.1%
DQB1	-6	3	0.63	82.8%	0.61	83.0%	0.98	98.7%	0.97	98.5%
DQB1	-5	4	0.63	81.1%	0.60	81.7%	0.97	97.9%	0.97	98.6%
DQB1	-4	3	0.64	82.9%	0.61	83.1%	0.98	98.7%	0.97	98.5%
DQB1	3	2	NaN	99.0%	NaN	99.0%	0.93	99.9%	0.89	99.9%
DQB1	9	3	0.56	87.6%	0.60	89.2%	0.93	97.5%	0.96	98.3%
DQB1	13	2	0.65	88.3%	0.63	87.7%	0.95	98.5%	0.96	99.2%
DQB1	14	2	0.61	88.4%	0.62	89.8%	0.96	98.6%	0.98	99.6%
DQB1	26	3	0.67	82.4%	0.64	82.2%	0.95	97.4%	0.97	99.1%
DQB1	28	2	0.67	89.2%	0.66	88.7%	0.96	98.7%	0.96	98.8%
DQB1	30	3	0.62	78.9%	0.61	79.4%	0.94	96.6%	0.96	97.8%
DQB1	37	3	0.67	89.0%	0.66	88.4%	0.96	98.7%	0.96	98.8%
DQB1	38	2	0.59	81.8%	0.60	83.1%	0.95	97.8%	0.96	98.8%
DQB1	45	2	0.67	89.0%	0.64	88.2%	0.95	98.5%	0.96	99.2%
DQB1	46	2	0.67	89.2%	0.66	88.7%	0.96	98.7%	0.96	98.8%
DQB1	47	2	0.67	89.2%	0.66	88.7%	0.96	98.7%	0.96	98.8%
DQB1	52	2	0.67	89.2%	0.66	88.6%	0.96	98.7%	0.96	98.8%
DQB1	53	2	0.63	83.4%	0.58	82.7%	0.99	99.2%	0.98	98.9%
DQB1	55	3	0.69	82.7%	0.64	81.2%	0.97	98.6%	0.97	98.6%
DQB1	56	2	0.79	98.9%	0.80	98.8%	1.00	100.0%	1.00	100.0%
DQB1	57	4	0.65	80.4%	0.67	82.3%	0.97	98.2%	0.96	98.5%
DQB1	66	2	0.69	88.6%	0.68	88.3%	0.96	98.6%	0.96	98.7%
DQB1	67	2	0.69	88.6%	0.68	88.3%	0.96	98.6%	0.96	98.7%
DQB1	70	3	0.64	83.5%	0.64	84.7%	0.99	99.0%	0.98	98.8%
DQB1	71	4	0.63	80.6%	0.62	81.4%	0.95	97.7%	0.96	98.8%
DQB1	74	3	0.63	80.7%	0.62	81.4%	0.95	97.8%	0.97	98.8%
DQB1	75	2	0.59	81.3%	0.59	82.3%	0.94	97.8%	0.96	98.8%
DQB1	77	2	0.58	81.6%	0.59	82.8%	0.94	97.8%	0.96	98.8%
DQB1	84	2	0.64	83.5%	0.58	82.8%	0.99	99.2%	0.98	98.9%
DQB1	85	2	0.64	83.4%	0.58	82.8%	0.99	99.2%	0.98	98.9%
DQB1	86	3	0.64	82.9%	0.59	82.4%	0.99	99.0%	0.98	98.8%
DQB1	87	3	0.63	81.4%	0.59	81.1%	0.98	98.3%	0.98	98.9%
DQB1	89	2	0.64	83.5%	0.58	82.8%	0.99	99.2%	0.98	98.9%

DQB1	90	2	0.64	83.5%	0.58	82.8%	0.99	99.2%	0.98	98.9%
DQB1	116	2	0.62	88.8%	0.62	89.7%	0.95	98.5%	0.98	99.5%
DQB1	125	3	0.64	81.9%	0.59	82.3%	0.97	98.2%	0.98	98.9%
DQB1	126	2	0.56	98.7%	0.57	98.8%	0.89	99.7%	0.79	99.7%
DQB1	130	2	0.75	98.2%	0.53	97.0%	0.98	99.8%	0.98	99.8%
DQB1	135	2	0.62	93.7%	0.55	92.0%	0.95	99.4%	0.94	99.4%
DQB1	140	2	0.74	88.4%	0.69	87.3%	0.97	99.0%	0.96	99.1%
DQB1	167	2	0.65	88.2%	0.65	88.5%	0.95	98.4%	0.96	99.1%
DQB1	182	2	0.74	88.4%	0.69	87.3%	0.97	99.0%	0.96	99.1%
DQB1	185	2	0.70	91.1%	0.71	92.1%	0.95	98.8%	0.97	99.5%
DQB1	197	2	NaN	99.0%	NaN	99.0%	0.93	99.9%	0.89	99.9%
DQB1	203	2	0.33	84.2%	0.32	85.0%	0.98	98.9%	0.97	98.6%
DQB1	220	2	0.32	83.7%	0.31	84.5%	0.98	98.9%	0.97	98.6%
DQB1	221	2	0.32	83.8%	0.31	84.5%	0.98	98.9%	0.97	98.5%
DQB1	224	2	0.32	88.9%	0.31	89.5%	0.94	98.2%	0.96	99.2%
DRB1	-25	3	0.80	89.3%	0.77	90.5%	0.95	98.3%	0.96	98.9%
DRB1	-24	3	0.86	93.8%	0.86	96.1%	0.92	97.3%	0.95	98.7%
DRB1	-17	3	0.78	90.2%	0.77	90.8%	0.96	98.4%	0.97	99.1%
DRB1	-16	3	0.80	89.3%	0.77	90.5%	0.95	98.3%	0.96	98.9%
DRB1	-1	3	0.81	93.2%	0.78	93.4%	0.94	98.5%	0.95	98.9%
DRB1	4	2	0.38	92.9%	0.41	95.1%	0.92	97.8%	0.97	99.2%
DRB1	9	2	0.40	90.6%	0.40	92.0%	0.96	98.0%	0.99	99.5%
DRB1	10	3	0.81	89.9%	0.81	91.6%	0.97	99.3%	0.98	99.6%
DRB1	11	5	0.05	89.3%	0.05	92.1%	0.97	98.3%	0.99	99.4%
DRB1	12	2	0.84	91.3%	0.84	92.8%	0.97	99.4%	0.98	99.6%
DRB1	13	6	0.86	89.0%	0.87	91.1%	0.96	97.2%	0.99	99.1%
DRB1	14	2	0.84	95.8%	0.92	98.4%	0.97	99.2%	0.98	99.5%
DRB1	16	2	0.80	98.4%	0.82	98.3%	0.84	98.6%	0.96	99.5%
DRB1	25	2	0.84	95.8%	0.92	98.4%	0.97	99.2%	0.98	99.5%
DRB1	26	3	0.76	88.7%	0.85	92.2%	0.95	97.5%	0.98	99.2%
DRB1	28	2	0.39	92.3%	0.41	93.7%	0.93	97.0%	0.99	99.5%
DRB1	30	5	0.11	91.1%	0.13	93.3%	0.95	97.4%	0.99	99.5%
DRB1	31	3	0.72	93.6%	0.73	93.5%	0.95	98.7%	1.00	100.0%

DRB1	32	2	0.87	93.7%	0.85	94.1%	0.91	96.5%	0.98	99.4%
DRB1	33	2	0.90	95.2%	0.91	97.4%	0.95	98.4%	0.98	99.6%
DRB1	37	5	0.78	85.5%	0.86	91.4%	0.93	95.7%	0.98	99.2%
DRB1	38	3	0.34	96.4%	0.64	98.2%	0.90	99.1%	1.00	99.9%
DRB1	40	2	0.00	98.2%	0.00	98.3%	1.00	100.0%	1.00	100.0%
DRB1	47	2	0.84	90.9%	0.89	94.2%	0.95	97.9%	0.97	99.2%
DRB1	57	4	0.66	88.3%	0.80	93.0%	0.89	95.8%	0.97	99.2%
DRB1	58	2	0.64	96.0%	0.75	97.7%	0.81	98.5%	0.93	99.3%
DRB1	60	3	0.66	89.1%	0.83	94.6%	0.91	96.6%	0.98	99.4%
DRB1	67	3	0.75	86.5%	0.79	89.8%	0.87	93.9%	0.95	97.8%
DRB1	70	3	0.67	83.3%	0.76	88.6%	0.89	94.5%	0.96	98.3%
DRB1	71	4	0.70	80.6%	0.78	88.0%	0.87	92.9%	0.94	97.0%
DRB1	73	2	0.83	91.7%	0.90	95.0%	0.98	98.9%	0.98	99.1%
DRB1	74	5	0.75	86.6%	0.78	89.3%	0.89	95.0%	0.94	97.5%
DRB1	77	2	0.86	94.8%	0.88	96.0%	0.97	99.2%	0.96	99.2%
DRB1	78	2	0.76	93.2%	0.82	95.2%	0.95	98.4%	0.98	99.6%
DRB1	85	2	0.36	96.9%	0.69	98.6%	0.88	99.1%	1.00	99.9%
DRB1	86	2	0.65	83.4%	0.78	90.0%	0.82	91.6%	0.88	95.1%
DRB1	96	4	0.86	90.7%	0.87	93.2%	0.97	98.3%	0.99	99.6%
DRB1	98	2	0.84	92.1%	0.90	95.8%	0.98	99.5%	0.98	99.5%
DRB1	104	2	0.84	92.1%	0.90	95.8%	0.98	99.5%	0.98	99.5%
DRB1	112	2	0.47	96.9%	0.83	99.1%	0.76	98.2%	0.90	99.2%
DRB1	120	2	0.85	93.5%	0.87	96.0%	0.95	98.4%	0.98	99.5%
DRB1	133	2	0.93	96.6%	0.90	97.2%	1.00	99.8%	1.00	99.9%
DRB1	140	2	0.85	91.9%	0.81	92.0%	0.96	98.0%	0.99	99.5%
DRB1	142	2	0.93	96.9%	0.90	97.1%	1.00	99.8%	1.00	99.9%
DRB1	149	2	0.87	92.8%	0.85	93.4%	0.97	99.3%	0.98	99.6%
DRB1	166	2	0.00	98.2%	0.00	97.9%	1.00	99.9%	1.00	99.9%
DRB1	180	3	0.87	94.2%	0.92	97.7%	0.94	98.0%	0.97	99.3%
DRB1	181	3	0.72	91.2%	0.76	92.9%	0.94	98.1%	0.97	99.3%
DRB1	231	3	0.52	96.5%	0.55	96.5%	0.86	98.8%	0.90	99.2%
DRB1	233	3	0.86	92.4%	0.85	93.3%	0.95	98.0%	0.97	99.0%

Table S4: Imputation performance for all imputed HLA amino acids is shown for four imputation scenarios, each using a large (T1DGC) or small (HapMap-CEPH) reference panel, and a high-density (Illumina Immunochip) or low-density (Affymetrix 500K) SNP chip. Allele frequencies are shown for imputed and known amino acid residues in 918 individuals from the British 1958 Birth Cohort. R^2 correlation (between imputed and genotyped dosages) and accuracy (genotype concordance) is shown for each amino acid allele at *HLA-A*, *-B*, *-C*, *-DQB1*, and *-DRB1*. Allele "x" denotes deletion/truncation. Accuracy was based on the same set of variants, allowing a direct and fair comparison between reference panels and SNP chips.

				**HapMap-CEPH (n=90)**						**T1DGC (n=5,225)**					
				**Affymetrix 500K**			**Illumina ImmunoChip**			**Affymetrix 500K**			**Illumina ImmunoChip**		
**locus**	**pos**	**allele**	**frq**	**frq**	***r2***	**accuracy**	**frq**	***r2***	**accuracy**	**frq**	***r2***	**accuracy**	**frq**	***r2***	**accuracy**
A	-15	L	51.7%	50.1%	0.89	96.7%	52.3%	0.97	98.5%	51.4%	0.99	99.7%	51.5%	0.99	99.6%
A	-15	V	48.2%	49.9%	0.89	96.8%	47.7%	0.97	98.5%	48.5%	0.99	99.7%	48.4%	0.99	99.7%
A	-15	x	0.1%	NA	NA	NA	NA	NA	NA	0.1%	0.14	99.8%	0.1%	0.39	99.9%
A	-11	S	88.7%	88.9%	0.98	99.6%	88.9%	0.99	99.8%	88.8%	0.99	99.8%	88.7%	0.99	99.9%
A	-11	L	11.2%	11.1%	0.98	99.6%	11.1%	0.99	99.8%	11.1%	0.99	99.8%	11.2%	0.99	99.9%
A	-11	x	0.1%	NA	NA	NA	NA	NA	NA	0.1%	0.14	99.8%	0.1%	0.39	99.9%
A	9	F	65.9%	66.7%	0.92	97.6%	67.3%	0.94	97.8%	65.5%	0.97	99.2%	65.7%	0.98	99.5%
A	9	T	7.0%	6.3%	0.90	99.3%	6.9%	0.98	99.9%	7.0%	0.98	99.9%	7.0%	0.98	99.9%
A	9	S	11.4%	11.5%	0.94	99.0%	10.3%	0.93	98.5%	11.4%	0.98	99.7%	11.4%	0.98	99.7%
A	9	Y	15.8%	15.5%	0.92	98.3%	15.6%	0.95	99.0%	16.1%	0.97	99.4%	15.9%	0.98	99.7%
A	12	M	0.4%	NA	NA	NA	NA	NA	NA	0.4%	1.00	100.0%	0.4%	1.00	100.0%
A	12	V	99.6%	NA	NA	NA	NA	NA	NA	99.6%	1.00	100.0%	99.6%	1.00	100.0%
A	17	S	2.0%	0.2%	0.02	97.9%	1.8%	0.96	99.6%	2.0%	0.97	99.9%	2.0%	0.97	99.9%
A	17	R	98.0%	99.8%	0.02	97.9%	98.2%	0.96	99.6%	98.0%	0.97	99.9%	98.0%	0.97	99.9%
A	43	R	1.3%	0.6%	0.38	99.1%	1.3%	0.92	99.9%	1.4%	0.96	99.9%	1.4%	0.96	99.9%
A	43	Q	98.7%	99.4%	0.38	99.1%	98.7%	0.92	99.9%	98.6%	0.96	99.9%	98.6%	0.96	99.9%
A	44	K	18.7%	19.1%	0.95	98.7%	18.6%	0.98	99.6%	18.6%	0.98	99.6%	18.6%	0.98	99.6%
A	44	R	81.3%	80.9%	0.95	98.7%	81.4%	0.98	99.6%	81.4%	0.98	99.6%	81.4%	0.98	99.6%
A	56	G	95.7%	97.5%	0.47	97.8%	95.8%	0.95	99.5%	95.7%	0.99	99.9%	95.7%	0.99	99.9%
A	56	R	4.3%	2.5%	0.47	97.8%	4.2%	0.95	99.5%	4.3%	0.99	99.9%	4.3%	0.99	99.9%
A	62	Q	47.0%	46.1%	0.88	96.3%	47.9%	0.97	98.4%	46.8%	0.99	99.7%	46.8%	0.99	99.7%

A	62	G	30.4%	29.9%	0.94	98.3%	30.9%	0.96	99.3%	30.6%	0.98	99.7%	30.5%	0.98	99.7%
A	62	R	8.6%	8.7%	0.94	99.2%	8.2%	0.91	99.2%	8.6%	0.97	99.7%	8.7%	0.98	99.8%
A	62	E	9.3%	11.4%	0.78	97.6%	8.5%	0.92	98.8%	9.4%	0.98	99.8%	9.4%	0.98	99.8%
A	62	L	4.6%	3.9%	0.85	99.3%	4.5%	0.98	99.9%	4.6%	0.98	99.9%	4.6%	0.98	99.9%
A	63	E	86.8%	87.4%	0.91	98.6%	87.3%	0.94	99.2%	86.8%	0.98	99.7%	86.7%	0.99	99.8%
A	63	N	8.6%	8.7%	0.94	99.2%	8.2%	0.91	99.2%	8.6%	0.97	99.7%	8.7%	0.98	99.8%
A	63	Q	4.6%	3.9%	0.85	99.3%	4.5%	0.98	99.9%	4.6%	0.98	99.9%	4.6%	0.98	99.9%
A	65	G	9.3%	11.4%	0.78	97.6%	8.5%	0.92	98.8%	9.4%	0.98	99.8%	9.4%	0.98	99.8%
A	65	R	90.7%	88.6%	0.78	97.6%	91.5%	0.92	98.8%	90.6%	0.98	99.8%	90.6%	0.98	99.8%
A	66	K	39.8%	41.3%	0.88	96.5%	39.4%	0.96	98.3%	40.0%	0.99	99.7%	39.9%	0.99	99.7%
A	66	N	60.2%	58.7%	0.88	96.5%	60.6%	0.96	98.3%	60.0%	0.99	99.7%	60.1%	0.99	99.7%
A	67	M	18.7%	19.1%	0.95	98.7%	18.6%	0.98	99.6%	18.6%	0.98	99.6%	18.6%	0.98	99.6%
A	67	V	81.3%	80.9%	0.95	98.7%	81.4%	0.98	99.6%	81.4%	0.98	99.6%	81.4%	0.98	99.6%
A	70	Q	30.4%	28.0%	0.88	97.3%	29.9%	0.91	97.1%	30.5%	0.99	99.7%	30.6%	0.99	99.6%
A	70	H	69.6%	72.0%	0.88	97.3%	70.1%	0.91	97.1%	69.5%	0.99	99.7%	69.4%	0.99	99.6%
A	73	T	97.6%	97.6%	1.00	100.0%	97.6%	1.00	100.0%	97.6%	1.00	100.0%	97.6%	1.00	100.0%
A	73	I	2.4%	2.4%	1.00	100.0%	2.4%	1.00	100.0%	2.4%	1.00	100.0%	2.4%	1.00	100.0%
A	74	D	69.5%	70.1%	0.93	98.2%	69.1%	0.96	99.2%	69.4%	0.98	99.6%	69.5%	0.98	99.6%
A	74	H	30.5%	29.9%	0.93	98.2%	30.9%	0.96	99.2%	30.6%	0.98	99.6%	30.5%	0.98	99.6%
A	76	A	25.6%	24.2%	0.86	96.4%	23.9%	0.89	97.3%	25.4%	0.96	99.0%	25.3%	0.96	99.0%
A	76	V	58.7%	56.4%	0.87	96.4%	58.6%	0.92	97.0%	58.9%	0.98	99.5%	58.8%	0.98	99.5%
A	76	E	15.6%	19.4%	0.75	95.1%	17.4%	0.77	95.2%	15.7%	0.93	98.9%	15.9%	0.93	98.9%
A	77	N	35.6%	35.7%	0.82	95.0%	33.1%	0.87	95.6%	35.4%	0.96	99.0%	35.3%	0.96	99.0%
A	77	D	58.7%	56.4%	0.87	96.4%	58.7%	0.92	97.1%	58.9%	0.98	99.5%	58.8%	0.98	99.5%
A	77	S	5.7%	8.0%	0.70	97.2%	7.8%	0.72	97.4%	5.7%	0.88	99.2%	5.9%	0.88	99.2%
A	79	R	15.0%	19.3%	0.74	95.0%	16.3%	0.80	96.2%	15.2%	0.93	99.0%	15.3%	0.93	99.0%
A	79	G	85.0%	80.7%	0.74	95.0%	83.7%	0.80	96.2%	84.8%	0.93	99.0%	84.7%	0.93	99.0%
A	80	I	15.0%	19.3%	0.74	95.0%	16.2%	0.80	96.2%	15.2%	0.93	99.0%	15.3%	0.93	99.0%
A	80	T	85.0%	80.7%	0.74	95.0%	83.8%	0.80	96.2%	84.8%	0.93	99.0%	84.7%	0.93	99.0%
A	81	A	15.0%	19.3%	0.74	95.0%	16.3%	0.80	96.2%	15.2%	0.93	99.0%	15.3%	0.93	99.0%
A	81	L	85.0%	80.7%	0.74	95.0%	83.7%	0.80	96.2%	84.8%	0.93	99.0%	84.7%	0.93	99.0%
A	82	L	15.0%	19.3%	0.74	95.0%	16.2%	0.80	96.2%	15.2%	0.93	99.0%	15.3%	0.93	99.0%
A	82	R	85.0%	80.7%	0.74	95.0%	83.8%	0.80	96.2%	84.8%	0.93	99.0%	84.7%	0.93	99.0%

A	83	R	15.0%	19.3%	0.74	95.0%	16.2%	0.80	96.2%	15.2%	0.93	99.0%	15.3%	0.93	99.0%
A	83	G	85.0%	80.7%	0.74	95.0%	83.8%	0.80	96.2%	84.8%	0.93	99.0%	84.7%	0.93	99.0%
A	90	D	28.8%	29.3%	0.93	98.1%	28.7%	0.98	99.4%	28.7%	0.99	99.6%	28.8%	0.99	99.6%
A	90	A	71.2%	70.7%	0.93	98.1%	71.3%	0.98	99.4%	71.3%	0.99	99.6%	71.2%	0.99	99.6%
A	95	I	60.2%	58.6%	0.88	96.2%	60.5%	0.95	98.2%	60.0%	0.99	99.7%	60.1%	0.99	99.7%
A	95	V	29.1%	29.3%	0.98	99.0%	29.7%	0.97	99.2%	29.2%	0.99	99.7%	29.1%	0.99	99.7%
A	95	L	10.7%	12.2%	0.75	96.9%	9.8%	0.93	98.8%	10.9%	0.99	99.8%	10.8%	0.99	99.8%
A	97	I	40.6%	38.7%	0.90	96.8%	41.2%	0.97	98.4%	40.4%	0.99	99.7%	40.4%	0.99	99.7%
A	97	R	35.0%	34.5%	0.93	97.8%	35.3%	0.98	99.5%	35.1%	0.99	99.7%	35.1%	0.98	99.6%
A	97	M	24.4%	26.9%	0.86	96.8%	23.5%	0.95	98.5%	24.5%	0.99	99.6%	24.6%	0.98	99.6%
A	99	Y	90.7%	88.6%	0.78	97.6%	91.5%	0.92	98.8%	90.5%	0.98	99.7%	90.5%	0.98	99.7%
A	99	F	9.3%	11.4%	0.78	97.6%	8.5%	0.92	98.8%	9.5%	0.98	99.8%	9.5%	0.98	99.8%
A	105	P	33.4%	33.9%	0.90	97.0%	33.0%	0.96	98.9%	33.2%	0.99	99.5%	33.3%	0.98	99.5%
A	105	S	66.6%	66.1%	0.90	97.0%	67.0%	0.96	98.9%	66.8%	0.99	99.5%	66.7%	0.98	99.5%
A	107	W	30.4%	29.9%	0.94	98.1%	31.0%	0.96	99.1%	30.6%	0.98	99.7%	30.5%	0.98	99.7%
A	107	G	69.6%	70.1%	0.94	98.1%	69.0%	0.96	99.1%	69.4%	0.98	99.7%	69.5%	0.98	99.7%
A	109	L	4.3%	4.8%	0.89	99.4%	4.3%	0.99	99.9%	4.2%	0.98	99.9%	4.2%	0.99	99.9%
A	109	F	95.7%	95.2%	0.89	99.4%	95.7%	0.99	99.9%	95.8%	0.98	99.9%	95.8%	0.99	99.9%
A	114	R	47.0%	46.9%	0.93	97.5%	47.7%	0.97	98.2%	46.9%	0.99	99.5%	46.9%	0.98	99.5%
A	114	H	40.2%	41.3%	0.86	96.0%	39.5%	0.97	98.5%	40.4%	0.99	99.7%	40.3%	0.99	99.7%
A	114	Q	10.8%	11.6%	0.90	98.7%	11.0%	0.98	99.8%	10.7%	0.98	99.7%	10.8%	0.98	99.8%
A	114	E	2.0%	0.2%	0.02	97.8%	1.9%	0.93	99.6%	2.0%	0.97	99.9%	2.0%	0.97	99.9%
A	116	D	57.8%	58.6%	0.92	97.6%	58.6%	0.96	98.1%	57.6%	0.99	99.6%	57.7%	0.99	99.7%
A	116	Y	40.2%	41.3%	0.86	96.0%	39.5%	0.97	98.5%	40.4%	0.99	99.7%	40.3%	0.99	99.7%
A	116	H	2.0%	0.2%	0.01	97.8%	1.9%	0.93	99.6%	2.0%	0.97	99.9%	2.0%	0.97	99.9%
A	127	K	44.0%	45.7%	0.88	96.3%	43.3%	0.97	98.5%	44.3%	0.99	99.7%	44.2%	0.99	99.6%
A	127	N	56.0%	54.3%	0.88	96.3%	56.7%	0.97	98.5%	55.7%	0.99	99.7%	55.8%	0.99	99.6%
A	142	T	34.7%	34.3%	0.94	98.0%	34.9%	0.98	99.4%	34.9%	0.98	99.6%	34.8%	0.98	99.6%
A	142	I	65.3%	65.7%	0.94	98.0%	65.1%	0.98	99.4%	65.1%	0.98	99.6%	65.2%	0.98	99.6%
A	144	K	80.8%	80.6%	0.95	98.7%	81.0%	0.97	99.1%	80.9%	0.97	99.6%	80.8%	0.98	99.6%
A	144	Q	19.2%	19.4%	0.95	98.7%	19.0%	0.97	99.1%	19.1%	0.97	99.6%	19.2%	0.98	99.6%
A	145	H	34.7%	34.3%	0.94	98.0%	34.9%	0.98	99.4%	34.9%	0.98	99.7%	34.8%	0.98	99.6%
A	145	R	65.3%	65.7%	0.94	98.0%	65.1%	0.98	99.4%	65.1%	0.98	99.7%	65.2%	0.98	99.6%

A	149	T	4.3%	4.4%	0.88	99.2%	4.3%	0.96	99.8%	4.4%	0.96	99.7%	4.5%	0.96	99.6%
A	149	A	95.7%	95.6%	0.88	99.2%	95.7%	0.96	99.8%	95.6%	0.96	99.7%	95.5%	0.96	99.6%
A	150	V	18.7%	18.9%	0.95	98.6%	18.6%	0.98	99.5%	18.5%	0.98	99.6%	18.6%	0.98	99.6%
A	150	A	81.3%	81.1%	0.95	98.6%	81.4%	0.98	99.5%	81.5%	0.98	99.6%	81.4%	0.98	99.6%
A	151	R	14.8%	15.0%	0.96	99.1%	14.7%	0.95	99.1%	14.8%	0.98	99.7%	14.8%	0.98	99.8%
A	151	H	85.2%	85.0%	0.96	99.1%	85.3%	0.95	99.1%	85.2%	0.98	99.7%	85.2%	0.98	99.8%
A	152	A	24.6%	24.8%	0.96	98.5%	24.4%	0.98	99.4%	24.5%	0.98	99.6%	24.5%	0.98	99.6%
A	152	E	18.1%	18.3%	0.96	99.1%	19.2%	0.96	98.5%	18.1%	0.98	99.5%	18.2%	0.98	99.5%
A	152	V	55.4%	56.8%	0.88	96.4%	56.3%	0.92	96.9%	55.6%	0.99	99.4%	55.4%	0.99	99.4%
A	152	W	1.4%	0.1%	0.01	98.4%	0.1%	0.01	98.5%	1.4%	1.00	100.0%	1.4%	1.00	100.0%
A	152	R	0.5%	0.1%	0.05	99.4%	0.2%	0.16	99.4%	0.4%	0.89	99.9%	0.4%	0.89	99.9%
A	156	R	18.7%	18.9%	0.95	98.6%	18.5%	0.98	99.4%	18.5%	0.98	99.6%	18.5%	0.98	99.6%
A	156	L	57.8%	58.1%	0.96	98.5%	59.4%	0.94	97.7%	57.6%	0.98	99.2%	57.5%	0.97	99.2%
A	156	Q	13.8%	13.5%	0.96	99.4%	12.7%	0.92	98.5%	13.8%	0.96	99.4%	13.7%	0.96	99.5%
A	156	W	9.7%	9.5%	0.88	98.5%	9.4%	0.88	98.9%	10.0%	0.95	99.3%	10.2%	0.95	99.4%
A	158	V	18.7%	18.8%	0.95	98.5%	18.5%	0.98	99.4%	18.5%	0.98	99.5%	18.5%	0.98	99.5%
A	158	A	81.3%	81.2%	0.95	98.5%	81.5%	0.98	99.4%	81.5%	0.98	99.5%	81.5%	0.98	99.5%
A	161	D	13.9%	13.9%	0.99	99.9%	14.9%	0.96	98.6%	13.8%	1.00	99.9%	13.8%	1.00	99.9%
A	161	E	86.1%	86.1%	0.99	99.9%	85.1%	0.96	98.6%	86.2%	1.00	99.9%	86.2%	1.00	99.9%
A	163	R	28.8%	29.2%	0.93	97.9%	28.6%	0.98	99.3%	28.6%	0.99	99.5%	28.7%	0.99	99.5%
A	163	T	71.2%	70.8%	0.93	97.9%	71.4%	0.98	99.3%	71.3%	0.99	99.5%	71.3%	0.99	99.5%
A	166	D	27.9%	30.2%	0.86	96.5%	27.0%	0.95	98.4%	27.8%	0.98	99.3%	27.8%	0.98	99.4%
A	166	E	72.1%	69.8%	0.86	96.5%	73.0%	0.95	98.4%	72.2%	0.98	99.3%	72.2%	0.98	99.4%
A	167	G	27.9%	30.2%	0.86	96.4%	26.9%	0.95	98.3%	27.8%	0.98	99.3%	27.8%	0.98	99.4%
A	167	W	72.1%	69.8%	0.86	96.4%	73.1%	0.95	98.3%	72.2%	0.98	99.3%	72.2%	0.98	99.4%
A	184	P	52.2%	52.6%	0.95	98.3%	50.3%	0.92	97.7%	52.1%	0.99	99.7%	52.1%	0.99	99.7%
A	184	A	47.7%	47.4%	0.95	98.2%	49.7%	0.92	97.6%	47.9%	0.99	99.7%	47.9%	0.99	99.7%
A	193	P	49.8%	50.3%	0.95	98.4%	47.9%	0.92	97.7%	49.7%	0.99	99.7%	49.7%	0.99	99.7%
A	193	A	50.1%	49.7%	0.95	98.3%	52.1%	0.92	97.6%	50.3%	0.99	99.7%	50.3%	0.99	99.7%
A	194	I	49.8%	50.3%	0.95	98.4%	47.9%	0.92	97.7%	49.7%	0.99	99.7%	49.7%	0.99	99.7%
A	194	V	50.1%	49.7%	0.95	98.3%	52.1%	0.92	97.6%	50.3%	0.99	99.7%	50.3%	0.99	99.7%
A	207	G	49.8%	50.4%	0.95	98.3%	48.0%	0.92	97.7%	49.7%	0.99	99.7%	49.7%	0.99	99.7%
A	207	S	50.1%	49.6%	0.95	98.3%	52.0%	0.92	97.7%	50.3%	0.99	99.7%	50.3%	0.99	99.7%

A	245	A	95.7%	95.4%	0.95	99.5%	96.1%	0.85	99.3%	95.7%	0.96	99.8%	95.7%	0.95	99.7%
A	245	V	4.3%	4.6%	0.96	99.5%	3.9%	0.87	99.4%	4.3%	0.97	99.9%	4.3%	0.96	99.8%
A	246	A	84.5%	84.4%	0.95	98.9%	84.5%	0.97	99.6%	84.6%	0.98	99.7%	84.5%	0.99	99.7%
A	246	S	15.4%	15.6%	0.94	98.8%	15.5%	0.96	99.5%	15.4%	0.98	99.7%	15.4%	0.98	99.7%
A	253	E	49.8%	50.4%	0.95	98.5%	47.9%	0.92	97.8%	49.7%	0.99	99.8%	49.7%	0.99	99.7%
A	253	Q	50.1%	49.6%	0.95	98.4%	52.1%	0.92	97.8%	50.2%	0.99	99.7%	50.2%	0.99	99.7%
A	276	L	40.5%	38.8%	0.90	97.0%	39.4%	0.91	96.8%	40.3%	0.99	99.7%	40.3%	0.99	99.7%
A	276	P	59.4%	61.2%	0.90	96.9%	60.6%	0.91	96.7%	59.6%	0.99	99.6%	59.5%	0.99	99.6%
A	276	x	0.1%	NA	NA	NA	NA	NA	NA	0.1%	0.01	99.8%	0.2%	0.39	99.8%
A	282	I	90.6%	88.5%	0.74	97.2%	91.5%	0.91	98.7%	90.5%	0.97	99.6%	90.4%	0.97	99.6%
A	282	V	9.3%	11.5%	0.75	97.3%	8.5%	0.92	98.8%	9.4%	0.98	99.8%	9.4%	0.98	99.8%
A	282	x	0.1%	NA	NA	NA	NA	NA	NA	0.1%	0.01	99.8%	0.2%	0.39	99.8%
A	283	P	98.2%	96.1%	0.40	97.7%	98.4%	0.86	99.6%	98.2%	0.91	99.8%	98.1%	0.93	99.8%
A	283	H	1.7%	3.9%	0.43	97.8%	1.6%	0.91	99.7%	1.7%	0.97	99.9%	1.7%	0.97	99.9%
A	283	x	0.1%	NA	NA	NA	NA	NA	NA	0.1%	0.00	99.8%	0.2%	0.39	99.8%
A	294	L	49.8%	50.3%	0.95	98.5%	47.9%	0.92	97.8%	49.7%	0.99	99.8%	49.7%	0.99	99.7%
A	294	F	50.1%	49.7%	0.95	98.4%	52.1%	0.92	97.7%	50.2%	0.99	99.7%	50.1%	0.99	99.6%
A	294	x	0.1%	NA	NA	NA	NA	NA	NA	0.1%	0.01	99.8%	0.2%	0.39	99.8%
A	297	V	95.7%	95.2%	0.87	99.3%	95.7%	0.97	99.9%	95.7%	0.97	99.8%	95.6%	0.98	99.8%
A	297	M	4.2%	4.8%	0.87	99.3%	4.3%	0.97	99.9%	4.2%	0.97	99.9%	4.2%	0.97	99.9%
A	297	x	0.1%	NA	NA	NA	NA	NA	NA	0.1%	0.01	99.8%	0.2%	0.39	99.8%
A	298	I	88.7%	88.9%	0.98	99.7%	88.8%	0.98	99.8%	88.8%	0.99	99.8%	88.7%	0.99	99.8%
A	298	F	11.2%	11.1%	0.98	99.7%	11.2%	0.98	99.8%	11.1%	0.99	99.9%	11.2%	0.99	99.9%
A	298	x	0.1%	NA	NA	NA	NA	NA	NA	0.1%	0.01	99.8%	0.2%	0.39	99.8%
A	299	T	84.5%	84.3%	0.95	98.8%	84.5%	0.97	99.6%	84.5%	0.98	99.6%	84.4%	0.99	99.7%
A	299	A	15.4%	15.7%	0.94	98.7%	15.5%	0.96	99.5%	15.4%	0.98	99.7%	15.4%	0.98	99.7%
A	299	x	0.1%	NA	NA	NA	NA	NA	NA	0.1%	0.01	99.8%	0.2%	0.39	99.8%
A	307	M	88.7%	88.9%	0.98	99.7%	88.8%	0.98	99.8%	88.8%	0.99	99.8%	88.7%	0.99	99.8%
A	307	R	11.2%	11.1%	0.98	99.7%	11.2%	0.98	99.8%	11.1%	0.99	99.9%	11.2%	0.99	99.9%
A	307	x	0.1%	NA	NA	NA	NA	NA	NA	0.1%	0.01	99.8%	0.2%	0.39	99.8%
A	311	K	90.6%	88.5%	0.74	97.2%	91.5%	0.91	98.7%	90.5%	0.97	99.6%	90.4%	0.97	99.6%
A	311	N	9.3%	11.5%	0.75	97.4%	8.5%	0.92	98.8%	9.4%	0.98	99.8%	9.4%	0.98	99.7%
A	311	x	0.1%	NA	NA	NA	NA	NA	NA	0.1%	0.01	99.8%	0.2%	0.39	99.8%

A	321	T	40.5%	38.9%	0.90	97.0%	39.4%	0.91	96.8%	40.3%	0.99	99.8%	40.3%	0.99	99.7%
A	321	S	59.4%	61.1%	0.90	97.0%	60.6%	0.91	96.7%	59.6%	0.99	99.6%	59.5%	0.99	99.6%
A	321	x	0.1%	NA	NA	NA	NA	NA	NA	0.1%	0.00	99.8%	0.2%	0.39	99.8%
A	334	V	84.5%	84.3%	0.95	98.8%	84.5%	0.97	99.6%	84.6%	0.98	99.7%	84.4%	0.99	99.7%
A	334	M	15.4%	15.7%	0.94	98.8%	15.5%	0.96	99.5%	15.4%	0.98	99.7%	15.4%	0.98	99.8%
A	334	x	0.1%	NA	NA	NA	NA	NA	NA	0.0%	0.01	99.8%	0.1%	0.39	99.9%
C	339	T	34.0%	37.0%	0.84	95.7%	34.9%	0.94	98.7%	34.1%	0.96	99.1%	34.2%	0.96	99.2%
C	339	A	66.0%	63.1%	0.84	95.8%	64.9%	0.94	98.6%	65.8%	0.96	99.1%	65.8%	0.97	99.3%
C	326	C	34.0%	36.7%	0.84	95.5%	34.9%	0.94	98.7%	34.1%	0.96	99.0%	34.2%	0.96	99.2%
C	326	S	66.0%	62.9%	0.84	95.6%	64.3%	0.94	98.0%	65.8%	0.96	99.1%	65.8%	0.97	99.3%
C	309	C	99.3%	99.9%	0.16	99.4%	99.9%	0.09	99.4%	99.4%	0.85	99.8%	99.3%	0.92	99.9%
C	309	H	0.6%	0.1%	0.17	99.4%	0.1%	0.09	99.4%	0.6%	0.93	99.9%	0.6%	1.00	100.0%
C	308	M	99.3%	99.9%	0.16	99.4%	99.9%	0.09	99.4%	99.4%	0.85	99.8%	99.3%	0.92	99.9%
C	308	I	0.6%	0.1%	0.17	99.5%	0.1%	0.09	99.4%	0.6%	0.93	99.9%	0.6%	1.00	100.0%
C	307	M	34.0%	37.0%	0.84	95.8%	34.8%	0.94	98.6%	34.1%	0.96	99.0%	34.2%	0.96	99.2%
C	307	V	66.0%	63.0%	0.84	95.8%	65.2%	0.94	98.7%	65.8%	0.96	99.1%	65.8%	0.97	99.3%
C	306	A	34.6%	37.1%	0.82	95.3%	34.8%	0.96	99.1%	34.7%	0.96	99.1%	34.8%	0.96	99.2%
C	306	V	65.4%	62.9%	0.82	95.4%	65.2%	0.97	99.2%	65.3%	0.97	99.1%	65.2%	0.97	99.3%
C	305	T	34.0%	37.0%	0.84	95.8%	34.9%	0.94	98.7%	34.1%	0.96	99.0%	34.2%	0.96	99.2%
C	305	A	66.0%	63.0%	0.84	95.8%	65.1%	0.94	98.7%	65.8%	0.96	99.1%	65.8%	0.97	99.3%
C	304	V	68.7%	71.0%	0.79	95.0%	68.4%	0.96	98.9%	68.8%	0.96	99.0%	68.9%	0.97	99.2%
C	304	M	31.2%	29.0%	0.80	95.0%	31.6%	0.96	98.9%	31.2%	0.97	99.1%	31.1%	0.97	99.3%
C	303	V	92.7%	92.6%	0.97	99.8%	92.7%	0.98	99.8%	92.6%	0.98	99.8%	92.6%	0.98	99.9%
C	303	M	7.3%	7.4%	0.98	99.8%	7.3%	0.98	99.9%	7.3%	0.99	99.9%	7.3%	0.99	99.9%
C	295	V	34.0%	37.0%	0.84	95.8%	34.8%	0.94	98.6%	34.1%	0.96	99.0%	34.2%	0.96	99.2%
C	295	A	66.0%	63.0%	0.84	95.8%	65.2%	0.94	98.6%	65.8%	0.96	99.1%	65.8%	0.97	99.3%
C	291	L	99.3%	99.9%	0.16	99.4%	99.9%	0.09	99.4%	99.4%	0.85	99.8%	99.3%	0.92	99.9%
C	291	P	0.6%	0.1%	0.17	99.5%	0.1%	0.09	99.4%	0.6%	0.93	99.9%	0.6%	1.00	100.0%
C	289	A	99.3%	99.9%	0.16	99.4%	99.9%	0.09	99.4%	99.4%	0.85	99.8%	99.3%	0.92	99.9%
C	289	S	0.6%	0.1%	0.17	99.5%	0.1%	0.09	99.4%	0.6%	0.93	99.9%	0.6%	1.00	100.0%
C	285	M	34.0%	37.0%	0.84	95.8%	34.8%	0.94	98.6%	34.1%	0.96	99.0%	34.2%	0.96	99.2%
C	285	V	65.4%	62.9%	0.82	95.4%	65.1%	0.97	99.3%	65.3%	0.97	99.1%	65.2%	0.97	99.3%
C	285	L	0.6%	0.1%	0.17	99.5%	0.1%	0.09	99.4%	0.6%	0.93	99.9%	0.6%	1.00	100.0%

C	284	I	99.3%	99.9%	0.16	99.4%	99.9%	0.09	99.4%	99.4%	0.85	99.8%	99.3%	0.92	99.9%
C	284	N	0.6%	0.1%	0.17	99.5%	0.1%	0.09	99.4%	0.6%	0.93	99.9%	0.6%	1.00	100.0%
C	275	E	76.6%	78.8%	0.76	95.6%	77.4%	0.94	98.9%	76.7%	0.97	99.4%	76.7%	0.97	99.5%
C	275	K	7.9%	7.5%	0.91	99.3%	7.3%	0.91	99.3%	7.9%	0.99	99.9%	8.0%	0.99	99.9%
C	275	G	15.4%	13.8%	0.66	95.5%	15.3%	0.97	99.6%	15.3%	0.96	99.6%	15.3%	0.96	99.6%
C	273	S	34.0%	37.0%	0.84	95.8%	34.8%	0.94	98.6%	34.1%	0.96	99.0%	34.2%	0.96	99.2%
C	273	R	66.0%	63.0%	0.84	95.8%	65.2%	0.94	98.7%	65.9%	0.97	99.1%	65.8%	0.97	99.3%
C	270	L	99.3%	99.9%	0.16	99.4%	99.9%	0.09	99.4%	99.4%	0.85	99.8%	99.3%	0.91	99.9%
C	270	C	0.6%	0.1%	0.17	99.4%	0.1%	0.09	99.4%	0.6%	0.93	99.9%	0.6%	1.00	100.0%
C	267	Q	34.6%	37.1%	0.82	95.3%	34.9%	0.96	99.2%	34.7%	0.96	99.0%	34.8%	0.97	99.2%
C	267	P	65.4%	62.9%	0.82	95.4%	65.1%	0.97	99.2%	65.3%	0.97	99.1%	65.1%	0.97	99.3%
C	261	M	34.0%	37.0%	0.84	95.8%	34.8%	0.94	98.6%	34.1%	0.96	99.0%	34.2%	0.96	99.2%
C	261	V	66.0%	63.0%	0.84	95.8%	65.2%	0.94	98.6%	65.9%	0.97	99.1%	65.8%	0.97	99.3%
C	253	Q	34.6%	37.1%	0.82	95.3%	34.9%	0.96	99.2%	34.7%	0.96	99.0%	34.8%	0.97	99.2%
C	253	E	65.4%	62.9%	0.82	95.4%	65.1%	0.97	99.2%	65.3%	0.97	99.1%	65.1%	0.97	99.3%
C	248	V	96.3%	96.4%	0.78	99.2%	97.2%	0.70	98.9%	96.3%	0.90	99.6%	96.3%	0.92	99.7%
C	248	M	3.6%	3.6%	0.80	99.2%	2.8%	0.71	99.0%	3.7%	0.91	99.7%	3.6%	0.93	99.8%
C	219	R	73.5%	73.4%	0.90	97.9%	73.4%	0.96	99.2%	73.4%	0.96	99.2%	73.5%	0.96	99.4%
C	219	W	26.4%	26.6%	0.90	98.0%	26.6%	0.97	99.3%	26.5%	0.96	99.3%	26.5%	0.97	99.5%
C	211	A	96.5%	96.8%	0.77	99.4%	96.8%	0.77	99.4%	96.7%	0.79	99.5%	96.6%	0.81	99.6%
C	211	T	3.5%	3.2%	0.78	99.5%	3.2%	0.78	99.5%	3.3%	0.80	99.6%	3.4%	0.82	99.7%
C	194	L	34.0%	37.0%	0.84	95.6%	34.7%	0.94	98.6%	34.1%	0.96	98.9%	34.2%	0.96	99.2%
C	194	V	66.0%	63.0%	0.84	95.7%	65.3%	0.94	98.6%	65.8%	0.96	99.0%	65.8%	0.97	99.3%
C	193	P	95.7%	95.9%	0.91	99.6%	96.0%	0.89	99.5%	95.7%	0.96	99.8%	95.7%	0.96	99.8%
C	193	L	4.3%	4.1%	0.92	99.6%	4.0%	0.90	99.6%	4.3%	0.97	99.9%	4.3%	0.97	99.9%
C	184	P	34.0%	37.0%	0.84	95.6%	34.7%	0.94	98.6%	34.1%	0.96	98.9%	34.2%	0.96	99.2%
C	184	H	65.4%	62.9%	0.82	95.3%	65.2%	0.97	99.2%	65.2%	0.96	99.0%	65.2%	0.97	99.3%
C	184	R	0.6%	0.1%	0.17	99.5%	0.1%	0.09	99.4%	0.6%	0.93	99.9%	0.6%	1.00	100.0%
C	177	K	17.4%	14.8%	0.75	96.3%	16.4%	0.83	97.6%	17.2%	0.95	99.4%	17.2%	0.96	99.5%
C	177	E	82.6%	85.2%	0.75	96.3%	83.6%	0.83	97.6%	82.8%	0.95	99.4%	82.8%	0.96	99.5%
C	173	K	14.7%	14.5%	0.92	99.0%	14.8%	0.97	99.7%	14.8%	0.96	99.6%	14.7%	0.96	99.6%
C	173	E	85.3%	85.5%	0.92	99.0%	85.2%	0.97	99.7%	85.2%	0.96	99.6%	85.3%	0.96	99.6%
C	170	G	0.6%	0.1%	0.16	99.4%	0.1%	0.09	99.4%	0.6%	0.93	99.9%	0.6%	1.00	100.0%

C	170	R	99.4%	99.9%	0.16	99.4%	99.9%	0.09	99.4%	99.4%	0.93	99.9%	99.4%	1.00	100.0%
C	163	T	81.2%	82.1%	0.88	98.1%	82.0%	0.91	98.9%	81.4%	0.95	99.4%	81.3%	0.96	99.5%
C	163	L	14.7%	14.5%	0.92	99.0%	14.8%	0.97	99.7%	14.8%	0.96	99.6%	14.7%	0.96	99.6%
C	163	E	4.1%	3.4%	0.69	98.8%	3.2%	0.68	98.9%	3.8%	0.82	99.5%	4.0%	0.85	99.6%
C	156	L	49.2%	51.1%	0.80	94.6%	50.7%	0.88	97.0%	49.5%	0.96	98.9%	49.6%	0.96	99.2%
C	156	W	17.4%	17.9%	0.85	97.5%	17.4%	0.93	99.2%	17.3%	0.92	99.0%	17.3%	0.93	99.2%
C	156	R	27.1%	24.7%	0.79	95.7%	27.2%	0.97	99.1%	27.1%	0.97	99.3%	27.0%	0.98	99.5%
C	156	D	2.0%	2.2%	0.84	99.5%	1.1%	0.10	97.9%	1.9%	0.89	99.8%	1.9%	0.91	99.8%
C	156	Q	4.3%	4.1%	0.92	99.6%	4.0%	0.90	99.6%	4.2%	0.97	99.8%	4.2%	0.97	99.8%
C	152	A	38.2%	41.2%	0.83	95.4%	38.8%	0.93	98.4%	38.5%	0.96	98.9%	38.5%	0.97	99.2%
C	152	E	61.8%	58.8%	0.83	95.4%	61.2%	0.93	98.4%	61.5%	0.96	98.9%	61.5%	0.97	99.2%
C	147	L	34.6%	37.2%	0.82	95.2%	34.8%	0.97	99.2%	34.7%	0.96	99.0%	34.8%	0.97	99.2%
C	147	W	65.4%	62.8%	0.82	95.2%	65.2%	0.97	99.2%	65.3%	0.96	99.0%	65.2%	0.97	99.2%
C	143	S	0.6%	0.1%	0.10	99.4%	0.1%	0.09	99.4%	0.6%	0.93	100.0%	0.6%	1.00	100.0%
C	143	T	99.4%	99.9%	0.10	99.4%	99.9%	0.09	99.4%	99.4%	0.93	100.0%	99.4%	1.00	100.0%
C	138	K	15.4%	12.7%	0.74	96.6%	15.4%	0.97	99.6%	15.3%	0.96	99.6%	15.3%	0.96	99.6%
C	138	T	84.6%	87.3%	0.74	96.6%	84.6%	0.97	99.6%	84.7%	0.96	99.6%	84.7%	0.96	99.6%
C	116	S	54.4%	58.0%	0.79	94.9%	56.5%	0.84	96.0%	54.7%	0.94	98.7%	54.6%	0.94	98.9%
C	116	Y	18.4%	18.0%	0.89	98.4%	17.5%	0.87	98.2%	18.4%	0.94	99.2%	18.3%	0.95	99.4%
C	116	F	25.4%	22.2%	0.78	95.6%	23.9%	0.89	97.8%	25.3%	0.96	99.1%	25.3%	0.96	99.2%
C	116	L	1.8%	1.7%	0.83	99.7%	2.1%	0.78	99.5%	1.7%	0.83	99.6%	1.7%	0.88	99.7%
C	114	D	76.7%	79.9%	0.78	96.0%	77.2%	0.94	98.8%	76.7%	0.97	99.5%	76.7%	0.97	99.6%
C	114	N	23.3%	20.1%	0.78	96.0%	22.8%	0.94	98.8%	23.3%	0.97	99.5%	23.3%	0.97	99.6%
C	113	H	1.9%	1.7%	0.84	99.7%	2.1%	0.81	99.6%	1.8%	0.91	99.7%	1.8%	0.93	99.8%
C	113	Y	98.1%	98.3%	0.84	99.7%	97.9%	0.81	99.6%	98.2%	0.91	99.7%	98.2%	0.93	99.8%
C	103	V	14.7%	14.4%	0.92	99.0%	14.7%	0.94	99.4%	14.8%	0.96	99.6%	14.7%	0.96	99.6%
C	103	L	85.3%	85.6%	0.92	99.0%	85.3%	0.94	99.4%	85.2%	0.96	99.6%	85.3%	0.96	99.6%
C	99	Y	73.3%	72.9%	0.91	98.1%	72.2%	0.90	98.0%	73.3%	0.96	99.3%	73.3%	0.96	99.4%
C	99	S	15.0%	14.9%	0.96	99.5%	16.2%	0.86	98.1%	14.9%	0.96	99.6%	15.0%	0.96	99.5%
C	99	F	8.0%	8.5%	0.90	99.1%	8.7%	0.88	99.0%	8.1%	0.98	99.8%	8.1%	0.98	99.8%
C	99	C	3.7%	3.6%	0.78	99.2%	2.8%	0.70	98.9%	3.7%	0.90	99.6%	3.6%	0.92	99.7%
C	97	R	77.9%	76.9%	0.87	97.1%	77.9%	0.91	98.5%	77.9%	0.95	99.0%	78.1%	0.95	99.2%
C	97	W	22.1%	23.1%	0.87	97.1%	22.1%	0.91	98.5%	22.1%	0.95	99.0%	21.9%	0.95	99.2%

C	95	L	80.8%	81.5%	0.89	98.2%	82.0%	0.83	97.5%	81.0%	0.95	99.3%	80.9%	0.96	99.5%
C	95	I	17.2%	16.2%	0.88	98.3%	16.9%	0.88	98.4%	17.2%	0.96	99.5%	17.2%	0.97	99.6%
C	95	F	2.0%	2.2%	0.84	99.5%	1.1%	0.10	97.9%	1.9%	0.89	99.7%	1.9%	0.91	99.8%
C	94	I	16.6%	16.1%	0.92	98.8%	16.8%	0.93	99.0%	16.7%	0.96	99.5%	16.7%	0.97	99.7%
C	94	T	83.4%	83.9%	0.92	98.8%	83.2%	0.93	99.0%	83.3%	0.96	99.5%	83.3%	0.97	99.7%
C	91	R	5.7%	5.9%	0.80	98.7%	5.9%	0.80	98.9%	5.8%	0.90	99.2%	5.9%	0.89	99.2%
C	91	G	94.3%	94.1%	0.80	98.7%	94.1%	0.80	98.9%	94.2%	0.90	99.2%	94.1%	0.89	99.2%
C	90	A	48.0%	44.7%	0.85	96.0%	46.9%	0.94	98.4%	47.7%	0.96	99.1%	47.8%	0.97	99.3%
C	90	D	52.0%	55.3%	0.85	96.0%	53.1%	0.94	98.4%	52.3%	0.96	99.1%	52.2%	0.97	99.3%
C	80	K	35.4%	33.4%	0.86	96.5%	36.4%	0.84	96.6%	35.4%	0.95	98.9%	35.3%	0.96	99.2%
C	80	N	64.6%	66.6%	0.86	96.5%	63.6%	0.84	96.6%	64.6%	0.95	98.9%	64.7%	0.96	99.2%
C	77	N	35.4%	33.4%	0.86	96.5%	36.4%	0.84	96.6%	35.4%	0.95	98.9%	35.3%	0.96	99.2%
C	77	S	64.6%	66.6%	0.86	96.5%	63.6%	0.84	96.6%	64.6%	0.95	98.9%	64.7%	0.96	99.2%
C	73	T	44.2%	40.8%	0.83	95.9%	43.6%	0.94	98.8%	44.0%	0.95	99.0%	43.9%	0.96	99.2%
C	73	A	55.8%	59.2%	0.83	95.9%	56.4%	0.94	98.8%	56.0%	0.95	99.0%	56.1%	0.96	99.2%
C	66	N	18.9%	21.8%	0.77	95.8%	19.6%	0.94	99.0%	19.3%	0.94	99.0%	19.2%	0.96	99.4%
C	66	K	81.1%	78.2%	0.77	95.8%	80.4%	0.94	99.0%	80.7%	0.94	99.0%	80.8%	0.96	99.4%
C	49	E	7.3%	7.4%	0.98	99.8%	7.3%	0.98	99.9%	7.3%	0.99	99.9%	7.3%	0.99	99.9%
C	49	A	92.7%	92.6%	0.98	99.8%	92.7%	0.98	99.9%	92.7%	0.99	99.9%	92.7%	0.99	99.9%
C	35	Q	15.4%	12.9%	0.74	96.5%	15.5%	0.96	99.4%	15.3%	0.96	99.6%	15.3%	0.97	99.7%
C	35	R	84.6%	87.1%	0.74	96.5%	84.5%	0.96	99.4%	84.7%	0.96	99.6%	84.7%	0.97	99.7%
C	24	S	48.4%	51.5%	0.84	95.8%	48.5%	0.90	97.5%	48.7%	0.96	99.0%	48.5%	0.97	99.3%
C	24	A	51.6%	48.5%	0.84	95.8%	51.5%	0.90	97.5%	51.3%	0.96	99.0%	51.5%	0.97	99.3%
C	21	H	20.1%	19.4%	0.91	98.6%	20.0%	0.91	98.7%	19.9%	0.95	99.3%	20.0%	0.96	99.6%
C	21	R	79.9%	80.6%	0.91	98.6%	80.0%	0.91	98.7%	80.1%	0.95	99.3%	80.0%	0.96	99.6%
C	16	S	3.5%	3.2%	0.78	99.5%	3.2%	0.78	99.5%	3.3%	0.80	99.5%	3.4%	0.82	99.6%
C	16	G	96.5%	96.8%	0.78	99.5%	96.8%	0.78	99.5%	96.7%	0.80	99.5%	96.6%	0.82	99.6%
C	14	W	7.3%	7.4%	0.98	99.8%	7.3%	0.98	99.9%	7.3%	0.99	99.9%	7.4%	0.99	99.9%
C	14	R	92.7%	92.6%	0.98	99.8%	92.7%	0.98	99.9%	92.7%	0.99	99.9%	92.6%	0.99	99.9%
C	11	S	11.7%	12.1%	0.88	98.5%	11.5%	0.97	99.7%	11.8%	0.97	99.6%	11.7%	0.98	99.8%
C	11	A	88.3%	87.9%	0.88	98.5%	88.5%	0.97	99.7%	88.2%	0.97	99.6%	88.3%	0.98	99.8%
C	9	D	44.7%	47.9%	0.85	96.2%	45.7%	0.94	98.5%	45.0%	0.97	99.1%	44.9%	0.97	99.4%
C	9	Y	43.6%	40.0%	0.81	95.2%	42.7%	0.94	98.6%	43.2%	0.95	99.0%	43.4%	0.96	99.4%

C	9	S	8.0%	8.5%	0.90	99.1%	8.7%	0.88	98.9%	8.1%	0.98	99.8%	8.1%	0.98	99.8%
C	9	F	3.7%	3.6%	0.78	99.2%	2.8%	0.70	98.9%	3.7%	0.90	99.6%	3.6%	0.92	99.7%
C	6	K	3.7%	3.6%	0.78	99.2%	2.8%	0.70	98.9%	3.7%	0.90	99.6%	3.6%	0.92	99.7%
C	6	R	96.3%	96.4%	0.78	99.2%	97.2%	0.70	98.9%	96.3%	0.90	99.6%	96.4%	0.92	99.7%
C	1	C	77.3%	78.1%	0.90	98.3%	78.0%	0.91	98.6%	77.3%	0.96	99.4%	77.2%	0.97	99.6%
C	1	G	22.6%	21.9%	0.91	98.3%	22.0%	0.92	98.7%	22.6%	0.97	99.5%	22.8%	0.97	99.6%
C	-5	T	99.3%	99.9%	0.08	99.4%	99.9%	0.09	99.4%	99.4%	0.85	99.9%	99.3%	0.92	99.9%
C	-5	I	0.6%	0.1%	0.09	99.4%	0.1%	0.09	99.4%	0.6%	0.93	100.0%	0.6%	1.00	100.0%
C	-9	G	34.0%	37.0%	0.84	95.7%	34.8%	0.94	98.7%	34.2%	0.96	99.0%	34.2%	0.96	99.3%
C	-9	A	66.0%	63.0%	0.84	95.8%	65.2%	0.95	98.8%	65.7%	0.96	99.0%	65.7%	0.97	99.3%
C	-15	L	39.9%	42.0%	0.81	95.0%	39.7%	0.94	98.8%	40.0%	0.94	98.7%	40.1%	0.95	99.0%
C	-15	I	60.0%	58.0%	0.81	95.0%	60.3%	0.94	98.8%	60.0%	0.94	98.8%	59.9%	0.95	99.1%
C	-17	A	34.6%	37.1%	0.82	95.4%	33.5%	0.91	97.9%	34.8%	0.96	99.0%	34.9%	0.97	99.3%
C	-17	T	65.4%	62.9%	0.82	95.4%	66.5%	0.91	98.0%	65.2%	0.96	99.0%	65.1%	0.97	99.3%
C	-18	R	99.3%	99.9%	0.08	99.4%	99.9%	0.09	99.4%	99.4%	0.85	99.9%	99.3%	0.92	99.9%
C	-18	Q	0.6%	0.1%	0.09	99.4%	0.1%	0.09	99.4%	0.6%	0.93	100.0%	0.6%	1.00	100.0%
B	325	C	65.6%	67.9%	0.80	94.2%	67.6%	0.87	96.6%	65.2%	0.97	98.9%	65.3%	0.98	99.6%
B	325	S	34.4%	32.1%	0.80	94.2%	32.4%	0.87	96.6%	34.6%	0.97	99.0%	34.5%	0.99	99.6%
B	307	M	99.5%	NA	NA	NA	NA	NA	NA	99.3%	0.92	99.7%	99.3%	0.97	99.8%
B	307	V	0.5%	NA	NA	NA	NA	NA	NA	0.5%	0.94	99.9%	0.5%	1.00	100.0%
B	305	A	71.9%	72.6%	0.84	95.7%	72.1%	0.73	93.6%	71.6%	0.96	98.8%	71.6%	0.98	99.4%
B	305	T	28.1%	27.4%	0.84	95.7%	27.9%	0.73	93.6%	28.2%	0.96	98.9%	28.2%	0.98	99.5%
B	282	V	71.9%	72.6%	0.84	95.7%	72.1%	0.73	93.6%	71.5%	0.96	98.7%	71.6%	0.98	99.4%
B	282	I	28.1%	27.4%	0.84	95.7%	27.9%	0.73	93.6%	28.3%	0.96	98.8%	28.2%	0.98	99.5%
B	199	A	81.9%	80.7%	0.91	97.8%	81.0%	0.92	98.4%	82.0%	0.96	99.1%	81.8%	0.98	99.5%
B	199	V	18.1%	19.3%	0.91	97.8%	19.0%	0.92	98.4%	17.8%	0.96	99.2%	18.1%	0.98	99.7%
B	194	I	90.9%	90.9%	0.72	97.0%	90.3%	0.92	98.4%	90.5%	0.95	99.1%	90.6%	0.96	99.5%
B	194	V	9.1%	9.1%	0.72	97.0%	9.7%	0.92	98.4%	9.3%	0.95	99.2%	9.3%	0.96	99.6%
B	180	E	35.6%	39.3%	0.76	93.6%	38.5%	0.79	94.9%	35.3%	0.96	98.5%	35.2%	0.97	99.1%
B	180	Q	64.4%	60.7%	0.76	93.6%	61.5%	0.79	94.9%	64.7%	0.96	98.5%	64.8%	0.97	99.1%
B	178	K	20.4%	24.1%	0.78	95.2%	24.1%	0.79	95.8%	20.5%	0.97	99.2%	20.5%	0.98	99.5%
B	178	T	79.6%	75.9%	0.78	95.2%	75.9%	0.79	95.8%	79.5%	0.97	99.2%	79.5%	0.98	99.5%
B	177	D	35.6%	39.3%	0.76	93.6%	38.5%	0.79	94.9%	35.3%	0.96	98.5%	35.2%	0.97	99.1%

B	177	E	64.4%	60.7%	0.76	93.6%	61.5%	0.79	94.9%	64.7%	0.96	98.5%	64.8%	0.97	99.1%
B	171	Y	88.3%	89.8%	0.73	96.5%	88.7%	0.76	96.5%	87.9%	0.95	99.2%	88.1%	0.97	99.4%
B	171	H	11.7%	10.2%	0.73	96.5%	11.3%	0.76	96.5%	12.1%	0.95	99.2%	11.9%	0.97	99.4%
B	167	W	80.7%	81.4%	0.87	97.5%	81.1%	0.86	97.5%	81.1%	0.96	99.2%	80.7%	0.98	99.6%
B	167	S	19.3%	18.6%	0.87	97.5%	18.9%	0.86	97.5%	18.9%	0.96	99.2%	19.2%	0.98	99.6%
B	163	E	28.1%	33.3%	0.71	92.3%	31.5%	0.80	95.3%	28.4%	0.97	99.0%	28.4%	0.98	99.4%
B	163	T	28.9%	23.8%	0.65	91.4%	24.8%	0.76	93.9%	28.7%	0.96	98.5%	28.6%	0.97	99.1%
B	163	L	43.1%	42.9%	0.85	95.3%	43.6%	0.92	97.3%	42.8%	0.96	98.4%	43.0%	0.98	99.2%
B	158	A	97.8%	99.2%	0.31	98.4%	98.1%	0.76	98.7%	97.8%	0.94	99.7%	97.7%	0.97	99.8%
B	158	T	2.2%	0.8%	0.31	98.4%	1.9%	0.76	98.7%	2.2%	0.94	99.7%	2.3%	0.97	99.8%
B	156	R	14.4%	17.9%	0.72	95.0%	17.9%	0.73	95.4%	14.4%	0.96	99.1%	14.4%	0.97	99.3%
B	156	D	30.5%	29.9%	0.83	95.4%	29.3%	0.85	96.2%	29.8%	0.96	98.6%	30.1%	0.97	98.9%
B	156	L	47.9%	45.0%	0.66	90.5%	45.7%	0.71	91.9%	48.7%	0.93	97.6%	48.3%	0.95	98.5%
B	156	W	7.3%	7.1%	0.85	98.7%	7.1%	0.94	99.5%	7.2%	0.95	99.4%	7.3%	0.97	99.8%
B	152	E	34.9%	35.3%	0.82	94.6%	36.6%	0.88	95.9%	35.1%	0.97	98.7%	35.0%	0.97	99.0%
B	152	V	65.1%	64.7%	0.82	94.6%	63.4%	0.88	95.9%	64.9%	0.97	98.7%	65.0%	0.97	99.0%
B	147	W	93.5%	93.5%	0.94	99.5%	93.6%	0.94	99.6%	93.4%	0.95	99.6%	93.5%	0.95	99.7%
B	147	L	6.5%	6.5%	0.94	99.5%	6.4%	0.94	99.6%	6.6%	0.95	99.6%	6.5%	0.95	99.7%
B	145	R	98.0%	97.9%	0.94	99.7%	97.9%	0.97	99.9%	97.9%	0.96	99.9%	97.9%	0.97	99.9%
B	145	L	2.0%	2.1%	0.94	99.7%	2.1%	0.97	99.9%	2.1%	0.96	99.9%	2.1%	0.97	99.9%
B	143	T	93.5%	93.5%	0.94	99.5%	93.6%	0.94	99.6%	93.4%	0.95	99.6%	93.5%	0.95	99.7%
B	143	S	6.5%	6.5%	0.94	99.5%	6.4%	0.94	99.6%	6.6%	0.95	99.6%	6.5%	0.95	99.7%
B	131	R	36.2%	40.1%	0.77	93.6%	39.0%	0.80	94.7%	36.2%	0.96	98.5%	36.0%	0.98	99.3%
B	131	S	63.8%	59.9%	0.77	93.6%	61.0%	0.80	94.7%	63.8%	0.96	98.5%	64.0%	0.98	99.3%
B	116	Y	40.8%	45.3%	0.73	92.2%	44.8%	0.78	93.6%	41.0%	0.95	98.2%	40.8%	0.97	99.0%
B	116	F	8.7%	5.9%	0.53	95.7%	6.8%	0.66	96.3%	8.9%	0.94	99.2%	8.7%	0.98	99.6%
B	116	D	23.2%	24.8%	0.83	96.0%	23.3%	0.95	98.6%	22.8%	0.96	98.9%	23.2%	0.98	99.5%
B	116	L	7.8%	6.3%	0.54	96.5%	5.9%	0.57	96.6%	7.9%	0.98	99.7%	7.9%	0.98	99.8%
B	116	S	19.5%	17.7%	0.73	95.2%	19.3%	0.86	97.4%	19.5%	0.93	98.4%	19.4%	0.96	99.2%
B	114	D	52.1%	54.5%	0.68	90.7%	56.4%	0.75	92.6%	51.8%	0.95	98.1%	52.0%	0.97	98.9%
B	114	N	42.8%	39.3%	0.66	90.6%	39.1%	0.75	93.0%	43.1%	0.95	98.1%	42.8%	0.97	99.0%
B	114	H	5.1%	6.2%	0.52	96.7%	4.6%	0.86	99.3%	5.1%	0.95	99.6%	5.2%	0.97	99.8%
B	113	H	67.6%	67.6%	0.79	94.4%	68.8%	0.88	96.9%	67.8%	0.97	98.9%	67.6%	0.98	99.5%

B	113	Y	32.4%	32.4%	0.79	94.4%	31.2%	0.88	96.9%	32.2%	0.97	99.0%	32.4%	0.98	99.5%
B	103	V	87.4%	89.6%	0.59	95.1%	89.4%	0.66	95.6%	87.1%	0.96	99.1%	87.3%	0.98	99.6%
B	103	L	12.6%	10.4%	0.59	95.1%	10.6%	0.66	95.6%	12.8%	0.96	99.2%	12.7%	0.98	99.6%
B	99	Y	97.6%	97.9%	0.68	99.0%	98.1%	0.70	99.2%	97.6%	0.92	99.7%	97.5%	0.95	99.8%
B	99	S	1.9%	2.1%	0.88	99.5%	1.9%	0.90	99.8%	1.9%	0.91	99.8%	1.9%	0.94	99.9%
B	99	F	0.5%	NA	NA	NA	NA	NA	NA	0.5%	0.94	99.9%	0.6%	1.00	100.0%
B	97	S	29.4%	33.6%	0.77	94.1%	32.8%	0.81	95.4%	29.4%	0.96	98.6%	29.3%	0.98	99.1%
B	97	R	48.2%	44.4%	0.80	93.6%	46.0%	0.92	96.6%	47.9%	0.97	98.3%	48.2%	0.98	98.9%
B	97	T	9.7%	9.1%	0.54	94.9%	9.3%	0.62	95.4%	10.0%	0.95	99.2%	9.9%	0.97	99.5%
B	97	W	4.1%	2.5%	0.51	98.0%	2.6%	0.52	97.9%	4.2%	0.99	99.9%	4.1%	0.96	99.8%
B	97	N	4.4%	6.2%	0.61	97.4%	4.6%	0.96	99.8%	4.4%	0.95	99.7%	4.6%	0.97	99.8%
B	97	V	4.2%	4.1%	0.93	99.7%	4.6%	0.86	99.4%	4.2%	0.93	99.7%	4.1%	0.97	99.9%
B	95	L	57.5%	58.9%	0.76	92.9%	57.3%	0.79	94.1%	57.7%	0.95	98.2%	57.6%	0.97	99.0%
B	95	I	28.9%	28.9%	0.88	96.8%	29.6%	0.90	97.3%	28.8%	0.95	98.6%	28.8%	0.97	99.4%
B	95	W	13.5%	12.2%	0.61	94.4%	13.1%	0.65	94.7%	13.6%	0.94	98.9%	13.6%	0.97	99.4%
B	94	I	27.0%	26.8%	0.88	97.0%	27.8%	0.90	97.3%	26.9%	0.95	98.5%	26.9%	0.98	99.4%
B	94	T	73.0%	73.2%	0.88	97.0%	72.2%	0.90	97.3%	73.1%	0.95	98.5%	73.1%	0.98	99.4%
B	83	R	38.4%	39.9%	0.82	94.6%	40.7%	0.81	94.7%	38.3%	0.94	98.1%	38.5%	0.96	98.8%
B	83	G	61.6%	60.1%	0.82	94.6%	59.3%	0.81	94.7%	61.7%	0.94	98.1%	61.5%	0.96	98.8%
B	82	L	38.4%	40.0%	0.82	94.5%	40.7%	0.81	94.7%	38.3%	0.94	98.1%	38.5%	0.96	98.8%
B	82	R	61.6%	60.0%	0.82	94.5%	59.3%	0.81	94.7%	61.7%	0.94	98.1%	61.5%	0.96	98.8%
B	81	A	31.5%	31.5%	0.80	94.9%	34.2%	0.79	94.6%	31.5%	0.95	98.3%	31.6%	0.97	98.8%
B	81	L	68.5%	68.5%	0.80	94.9%	65.8%	0.79	94.6%	68.5%	0.95	98.3%	68.4%	0.97	98.8%
B	80	N	61.6%	60.2%	0.82	94.6%	59.3%	0.81	94.6%	61.7%	0.94	98.1%	61.5%	0.96	98.8%
B	80	T	27.0%	29.0%	0.84	95.8%	27.1%	0.96	98.9%	26.8%	0.95	98.7%	27.1%	0.97	99.3%
B	80	I	11.3%	10.8%	0.66	95.8%	13.6%	0.64	95.1%	11.5%	0.93	98.8%	11.4%	0.94	99.0%
B	77	S	61.6%	60.2%	0.82	94.6%	59.3%	0.81	94.7%	61.6%	0.95	98.2%	61.5%	0.96	98.8%
B	77	D	6.9%	8.3%	0.60	96.4%	6.6%	0.86	99.1%	6.7%	0.92	99.4%	6.9%	0.94	99.7%
B	77	N	31.5%	31.4%	0.80	95.0%	34.2%	0.79	94.6%	31.5%	0.95	98.3%	31.6%	0.97	98.9%
B	74	D	41.1%	42.7%	0.87	95.3%	41.1%	0.95	98.0%	41.3%	0.96	98.4%	41.0%	0.97	99.1%
B	74	Y	58.9%	57.3%	0.87	95.3%	58.9%	0.95	98.0%	58.7%	0.96	98.4%	59.0%	0.97	99.1%
B	71	A	25.9%	28.5%	0.84	95.5%	27.8%	0.88	97.1%	26.2%	0.98	99.2%	26.0%	0.99	99.6%
B	71	T	74.1%	71.5%	0.84	95.5%	72.2%	0.88	97.1%	73.8%	0.98	99.2%	74.0%	0.99	99.6%

B	70	Q	16.5%	18.1%	0.90	97.7%	18.2%	0.89	97.9%	16.7%	0.97	99.3%	16.6%	0.98	99.7%
B	70	N	74.1%	71.5%	0.84	95.5%	72.2%	0.88	97.1%	73.8%	0.98	99.2%	74.0%	0.99	99.6%
B	70	S	4.9%	4.2%	0.79	99.0%	5.0%	0.82	99.1%	4.9%	0.94	99.6%	4.8%	0.97	99.8%
B	70	K	4.4%	6.2%	0.61	97.4%	4.6%	0.96	99.8%	4.6%	0.96	99.7%	4.5%	0.97	99.8%
B	69	A	25.9%	28.5%	0.84	95.5%	27.8%	0.88	97.1%	26.1%	0.98	99.3%	26.0%	0.99	99.6%
B	69	T	74.1%	71.5%	0.84	95.5%	72.2%	0.88	97.1%	73.8%	0.98	99.2%	74.0%	0.99	99.6%
B	67	Y	16.5%	18.0%	0.90	97.7%	18.2%	0.89	97.9%	16.7%	0.97	99.3%	16.7%	0.98	99.6%
B	67	F	22.5%	23.6%	0.83	95.8%	23.0%	0.93	97.8%	22.5%	0.94	98.4%	22.2%	0.95	99.1%
B	67	S	44.8%	43.9%	0.88	95.9%	44.6%	0.93	97.5%	44.6%	0.95	98.2%	45.1%	0.97	99.1%
B	67	C	11.2%	10.2%	0.62	95.8%	9.2%	0.73	96.7%	11.3%	0.95	99.2%	11.2%	0.97	99.6%
B	67	M	4.9%	4.2%	0.79	99.0%	5.0%	0.82	99.1%	4.9%	0.94	99.6%	4.8%	0.97	99.8%
B	66	I	95.1%	95.8%	0.79	99.0%	95.0%	0.82	99.1%	95.0%	0.94	99.6%	95.2%	0.97	99.8%
B	66	N	4.9%	4.2%	0.79	99.0%	5.0%	0.82	99.1%	4.9%	0.94	99.6%	4.8%	0.97	99.8%
B	65	R	4.9%	4.2%	0.79	99.0%	5.0%	0.82	99.1%	4.9%	0.94	99.6%	4.8%	0.97	99.8%
B	65	Q	95.1%	95.8%	0.79	99.0%	95.0%	0.82	99.1%	95.1%	0.94	99.6%	95.2%	0.97	99.8%
B	63	E	51.0%	51.5%	0.83	94.8%	50.9%	0.94	98.0%	50.7%	0.95	98.2%	51.2%	0.97	99.1%
B	63	N	49.0%	48.5%	0.83	94.8%	49.1%	0.94	98.0%	49.3%	0.95	98.2%	48.8%	0.97	99.1%
B	62	G	4.7%	4.2%	0.84	99.2%	5.0%	0.86	99.3%	4.7%	0.94	99.7%	4.6%	0.97	99.8%
B	62	R	95.3%	95.8%	0.84	99.2%	95.0%	0.86	99.3%	95.3%	0.94	99.7%	95.4%	0.97	99.8%
B	46	A	13.8%	13.4%	0.88	98.2%	13.7%	0.90	98.5%	13.8%	0.94	99.0%	13.7%	0.97	99.5%
B	46	E	86.2%	86.6%	0.88	98.2%	86.3%	0.90	98.5%	86.2%	0.94	99.0%	86.3%	0.97	99.5%
B	45	E	42.3%	43.5%	0.90	96.6%	41.8%	0.94	97.8%	42.4%	0.96	98.5%	42.2%	0.97	99.3%
B	45	T	14.2%	14.2%	0.83	97.1%	15.5%	0.89	97.7%	14.5%	0.94	98.9%	14.4%	0.96	99.4%
B	45	K	29.7%	29.0%	0.93	98.0%	28.9%	0.94	98.5%	29.3%	0.96	98.9%	29.6%	0.97	99.5%
B	45	M	13.8%	13.4%	0.88	98.2%	13.7%	0.90	98.5%	13.8%	0.94	99.0%	13.7%	0.97	99.5%
B	41	T	31.7%	31.1%	0.93	97.8%	30.8%	0.94	98.5%	31.4%	0.96	98.9%	31.7%	0.97	99.4%
B	41	A	68.3%	68.9%	0.93	97.8%	69.2%	0.94	98.5%	68.6%	0.96	98.9%	68.3%	0.97	99.4%
B	32	L	34.2%	35.2%	0.85	95.7%	33.4%	0.95	98.6%	33.7%	0.96	98.8%	34.2%	0.98	99.5%
B	32	Q	65.8%	64.8%	0.85	95.7%	66.6%	0.95	98.6%	66.3%	0.96	98.8%	65.8%	0.98	99.5%
B	30	G	3.2%	2.9%	0.58	98.3%	3.4%	0.96	99.6%	3.3%	0.95	99.7%	3.3%	0.98	99.9%
B	30	D	96.8%	97.1%	0.58	98.3%	96.6%	0.96	99.6%	96.7%	0.95	99.7%	96.7%	0.98	99.9%
B	24	S	40.3%	39.6%	0.83	94.9%	40.4%	0.94	97.6%	40.4%	0.96	98.6%	40.2%	0.98	99.4%
B	24	T	36.2%	37.3%	0.85	95.5%	35.5%	0.95	98.5%	36.0%	0.97	98.8%	36.3%	0.98	99.4%

B	24	A	23.5%	23.1%	0.82	96.2%	24.0%	0.88	97.0%	23.7%	0.96	99.0%	23.4%	0.98	99.6%
B	12	V	30.4%	30.8%	0.86	95.9%	30.7%	0.95	98.4%	30.8%	0.96	99.0%	30.8%	0.97	99.4%
B	12	M	69.6%	69.2%	0.86	95.9%	69.3%	0.95	98.4%	69.2%	0.96	99.0%	69.2%	0.97	99.4%
B	11	S	28.1%	28.4%	0.84	95.8%	28.3%	0.95	98.3%	28.3%	0.96	98.9%	28.4%	0.97	99.3%
B	11	A	71.9%	71.6%	0.84	95.8%	71.7%	0.95	98.3%	71.7%	0.96	98.9%	71.6%	0.97	99.3%
B	9	Y	64.9%	63.6%	0.81	94.4%	65.3%	0.93	98.0%	65.0%	0.96	98.6%	65.1%	0.98	99.3%
B	9	D	14.5%	14.8%	0.95	98.5%	14.5%	0.96	99.2%	14.4%	0.96	99.1%	14.2%	0.97	99.5%
B	9	H	20.6%	21.6%	0.76	95.1%	20.2%	0.93	98.5%	20.6%	0.96	99.0%	20.8%	0.98	99.5%
B	4	F	0.1%	NA	NA	NA	NA	NA	NA	0.1%	1.00	100.0%	0.1%	0.99	100.0%
B	4	S	99.9%	NA	NA	NA	NA	NA	NA	99.9%	1.00	100.0%	99.9%	0.99	100.0%
B	-8	L	55.8%	53.8%	0.86	95.7%	54.9%	0.91	96.9%	55.6%	0.95	98.3%	55.4%	0.96	99.1%
B	-8	V	44.2%	46.2%	0.86	95.7%	45.1%	0.91	96.9%	44.2%	0.95	98.3%	44.5%	0.96	99.1%
B	-10	A	45.1%	43.1%	0.85	95.8%	44.5%	0.88	96.8%	45.0%	0.94	98.3%	44.8%	0.96	99.2%
B	-10	G	54.9%	56.9%	0.85	95.8%	55.5%	0.88	96.8%	54.9%	0.94	98.3%	55.1%	0.96	99.2%
B	-11	S	53.1%	51.2%	0.86	95.7%	52.3%	0.91	96.9%	53.0%	0.95	98.3%	52.8%	0.96	99.1%
B	-11	W	46.9%	48.8%	0.86	95.7%	47.7%	0.91	96.9%	46.9%	0.95	98.3%	47.1%	0.96	99.1%
B	-16	V	66.5%	64.5%	0.80	94.6%	66.6%	0.94	98.4%	66.4%	0.95	98.5%	66.1%	0.97	99.3%
B	-16	L	33.5%	35.5%	0.80	94.6%	33.4%	0.94	98.4%	33.5%	0.95	98.6%	33.8%	0.97	99.4%
B	-21	M	34.7%	33.5%	0.89	96.8%	34.8%	0.92	97.7%	34.8%	0.96	98.6%	34.5%	0.98	99.4%
B	-21	T	65.3%	66.5%	0.89	96.8%	65.2%	0.92	97.7%	65.0%	0.96	98.6%	65.3%	0.98	99.4%
B	-23	L	34.7%	33.5%	0.89	96.8%	34.8%	0.92	97.7%	34.8%	0.96	98.6%	34.5%	0.98	99.4%
B	-23	R	65.3%	66.5%	0.89	96.8%	65.2%	0.92	97.7%	65.0%	0.96	98.6%	65.3%	0.98	99.4%
DRB1	233	R	31.8%	32.0%	0.88	95.2%	33.1%	0.86	95.9%	31.3%	0.95	98.6%	31.7%	0.98	99.5%
DRB1	233	T	65.3%	65.7%	0.86	95.1%	64.7%	0.85	95.5%	65.7%	0.96	99.0%	65.6%	0.97	99.4%
DRB1	233	x	2.9%	2.4%	0.68	99.0%	2.2%	0.72	99.1%	3.0%	0.82	99.2%	2.7%	0.87	99.5%
DRB1	231	Q	96.3%	97.6%	0.52	98.1%	97.6%	0.55	98.1%	96.1%	0.86	99.2%	96.5%	0.90	99.5%
DRB1	231	x	2.9%	2.4%	0.68	99.0%	2.2%	0.72	99.1%	3.0%	0.82	99.2%	2.7%	0.87	99.5%
DRB1	231	P	0.8%	0.0%	0.00	99.1%	0.2%	0.00	98.9%	0.8%	1.00	100.0%	0.8%	1.00	100.0%
DRB1	189	R	97.1%	NA	NA	NA	NA	NA	NA	96.6%	0.80	99.0%	97.2%	0.93	99.7%
DRB1	189	x	2.8%	NA	NA	NA	NA	NA	NA	3.0%	0.84	99.2%	2.7%	0.89	99.6%
DRB1	189	S	0.1%	NA	NA	NA	NA	NA	NA	0.4%	0.09	99.6%	0.1%	0.24	99.9%
DRB1	181	T	80.3%	80.7%	0.72	94.6%	82.2%	0.76	95.7%	80.2%	0.94	98.6%	80.3%	0.97	99.5%
DRB1	181	M	18.1%	17.7%	0.71	94.7%	16.6%	0.77	96.1%	18.0%	0.95	98.9%	18.0%	0.98	99.7%

DRB1	181	x	1.6%	1.6%	0.66	99.3%	1.4%	0.72	99.5%	1.8%	0.86	99.7%	1.7%	0.92	99.8%
DRB1	180	V	77.5%	77.0%	0.87	96.0%	76.4%	0.94	98.4%	77.1%	0.94	98.5%	77.2%	0.97	99.4%
DRB1	180	L	20.9%	21.4%	0.89	96.6%	22.1%	0.92	98.2%	21.1%	0.95	98.8%	21.1%	0.98	99.6%
DRB1	180	x	1.6%	1.6%	0.66	99.3%	1.4%	0.72	99.5%	1.8%	0.86	99.7%	1.7%	0.92	99.8%
DRB1	166	R	99.2%	99.9%	0.00	99.0%	99.6%	0.00	98.8%	99.1%	1.00	99.9%	99.1%	1.00	99.9%
DRB1	166	Q	0.8%	0.1%	0.00	99.0%	0.4%	0.00	98.8%	0.8%	1.00	100.0%	0.8%	1.00	100.0%
DRB1	149	H	34.7%	34.5%	0.87	94.9%	35.2%	0.85	95.3%	34.6%	0.97	99.4%	34.5%	0.98	99.6%
DRB1	149	Q	65.3%	65.5%	0.87	94.9%	64.8%	0.85	95.3%	65.3%	0.97	99.4%	65.5%	0.98	99.6%
DRB1	142	V	85.6%	85.7%	0.93	97.9%	85.6%	0.90	98.0%	85.5%	1.00	99.9%	85.5%	1.00	99.9%
DRB1	142	M	14.4%	14.3%	0.93	97.9%	14.4%	0.90	98.0%	14.4%	1.00	99.9%	14.4%	1.00	99.9%
DRB1	140	T	56.4%	56.0%	0.85	94.5%	57.5%	0.81	94.5%	56.5%	0.96	98.6%	56.4%	0.99	99.6%
DRB1	140	A	43.6%	44.0%	0.85	94.5%	42.5%	0.81	94.5%	43.4%	0.96	98.7%	43.6%	0.99	99.6%
DRB1	133	R	85.6%	85.8%	0.93	97.7%	85.5%	0.90	98.0%	85.5%	1.00	99.9%	85.5%	1.00	99.9%
DRB1	133	L	14.4%	14.2%	0.93	97.7%	14.5%	0.90	98.0%	14.4%	1.00	99.9%	14.4%	1.00	99.9%
DRB1	120	S	78.3%	78.4%	0.85	95.7%	77.7%	0.87	97.2%	78.0%	0.95	98.8%	78.0%	0.98	99.6%
DRB1	120	N	21.7%	21.6%	0.85	95.7%	22.3%	0.87	97.2%	21.9%	0.95	98.8%	21.9%	0.98	99.6%
DRB1	112	H	97.4%	97.2%	0.47	97.8%	97.5%	0.83	99.5%	97.9%	0.76	99.0%	97.5%	0.90	99.5%
DRB1	112	Y	2.6%	2.8%	0.47	97.8%	2.5%	0.83	99.5%	2.0%	0.75	99.0%	2.4%	0.90	99.5%
DRB1	104	S	61.9%	61.0%	0.84	94.6%	61.6%	0.90	97.3%	61.7%	0.98	99.5%	61.7%	0.98	99.5%
DRB1	104	A	38.1%	39.0%	0.84	94.6%	38.4%	0.90	97.3%	38.2%	0.98	99.6%	38.3%	0.98	99.6%
DRB1	98	K	61.9%	61.0%	0.84	94.6%	61.6%	0.90	97.3%	61.7%	0.98	99.5%	61.7%	0.98	99.5%
DRB1	98	E	38.1%	39.0%	0.84	94.6%	38.4%	0.90	97.3%	38.2%	0.98	99.6%	38.3%	0.98	99.6%
DRB1	96	H	52.0%	51.9%	0.86	93.8%	51.6%	0.87	95.2%	51.8%	0.96	98.7%	51.6%	0.99	99.6%
DRB1	96	Q	15.2%	14.4%	0.87	96.9%	14.5%	0.85	97.2%	15.3%	1.00	99.9%	15.3%	1.00	99.9%
DRB1	96	Y	20.9%	21.4%	0.89	96.6%	22.1%	0.92	98.2%	21.1%	0.95	98.8%	21.1%	0.98	99.6%
DRB1	96	E	12.0%	12.2%	0.82	97.8%	11.8%	0.83	98.1%	11.8%	0.98	99.8%	12.0%	1.00	100.0%
DRB1	86	V	46.2%	44.5%	0.65	88.5%	44.1%	0.78	93.2%	43.6%	0.82	94.4%	44.4%	0.88	96.6%
DRB1	86	G	53.8%	55.5%	0.65	88.5%	55.9%	0.78	93.2%	56.4%	0.82	94.4%	55.6%	0.88	96.6%
DRB1	85	A	2.4%	3.5%	0.36	97.6%	1.8%	0.69	99.3%	2.1%	0.88	99.5%	2.4%	1.00	100.0%
DRB1	85	V	97.6%	96.5%	0.36	97.6%	98.2%	0.69	99.3%	97.9%	0.88	99.5%	97.6%	1.00	100.0%
DRB1	78	V	17.2%	17.5%	0.76	95.6%	16.2%	0.82	97.2%	17.2%	0.95	98.9%	17.2%	0.98	99.7%
DRB1	78	Y	82.8%	82.5%	0.76	95.6%	83.8%	0.82	97.2%	82.8%	0.95	98.9%	82.8%	0.98	99.7%
DRB1	77	N	14.0%	13.5%	0.86	96.6%	13.8%	0.88	97.4%	13.7%	0.97	99.5%	13.8%	0.96	99.5%

DRB1	77	T	86.0%	86.5%	0.86	96.6%	86.2%	0.88	97.4%	86.3%	0.97	99.5%	86.2%	0.96	99.5%
DRB1	74	R	13.9%	13.5%	0.86	96.7%	13.8%	0.89	97.4%	13.7%	0.97	99.5%	13.8%	0.97	99.5%
DRB1	74	A	61.7%	62.0%	0.77	91.9%	64.3%	0.76	92.8%	62.3%	0.89	96.5%	62.4%	0.94	98.3%
DRB1	74	Q	15.6%	17.5%	0.84	96.9%	16.2%	0.92	98.8%	15.6%	0.97	99.4%	15.6%	0.98	99.7%
DRB1	74	L	2.0%	1.6%	0.65	99.2%	1.4%	0.70	99.3%	2.4%	0.78	99.3%	2.0%	0.97	99.9%
DRB1	74	E	6.7%	5.4%	0.30	93.7%	4.2%	0.44	96.1%	5.9%	0.63	96.8%	6.3%	0.79	98.3%
DRB1	73	G	29.6%	31.0%	0.83	94.1%	30.1%	0.90	96.6%	29.3%	0.98	99.2%	29.4%	0.98	99.4%
DRB1	73	A	70.4%	69.0%	0.83	94.1%	69.9%	0.90	96.6%	70.7%	0.98	99.2%	70.6%	0.98	99.4%
DRB1	71	K	27.2%	26.9%	0.73	91.4%	28.5%	0.80	94.4%	27.4%	0.87	96.7%	27.6%	0.91	98.1%
DRB1	71	A	13.8%	13.8%	0.94	98.0%	14.1%	0.91	98.0%	13.8%	0.99	99.8%	13.8%	1.00	99.9%
DRB1	71	E	11.8%	6.5%	0.46	92.7%	9.6%	0.68	95.5%	10.9%	0.82	97.2%	11.9%	0.94	99.0%
DRB1	71	R	47.2%	52.7%	0.66	87.9%	47.8%	0.74	92.4%	47.9%	0.84	94.7%	46.6%	0.93	97.8%
DRB1	70	Q	58.5%	60.6%	0.71	89.9%	61.6%	0.80	93.6%	59.2%	0.91	96.8%	58.6%	0.97	98.9%
DRB1	70	D	36.4%	36.2%	0.72	91.3%	35.5%	0.79	94.2%	35.9%	0.89	96.8%	36.2%	0.96	98.8%
DRB1	70	R	5.1%	3.2%	0.23	95.2%	2.8%	0.43	96.8%	4.9%	0.76	98.6%	5.1%	0.96	99.8%
DRB1	67	L	48.3%	50.0%	0.75	91.8%	50.4%	0.80	94.0%	48.9%	0.87	96.4%	48.4%	0.95	98.8%
DRB1	67	I	43.4%	41.8%	0.78	92.6%	42.3%	0.82	94.4%	41.9%	0.89	96.4%	43.6%	0.96	98.7%
DRB1	67	F	8.3%	8.2%	0.70	96.3%	7.2%	0.66	96.8%	9.3%	0.78	97.6%	8.1%	0.91	99.1%
DRB1	60	Y	78.4%	76.1%	0.66	92.2%	79.2%	0.84	96.7%	78.9%	0.91	97.8%	78.4%	0.98	99.5%
DRB1	60	S	19.0%	21.0%	0.68	94.0%	18.0%	0.82	97.1%	18.5%	0.93	98.5%	18.9%	0.98	99.7%
DRB1	60	H	2.7%	3.0%	0.46	97.6%	2.6%	0.86	99.4%	2.5%	0.76	99.2%	2.7%	0.93	99.8%
DRB1	58	A	95.4%	94.0%	0.64	96.9%	94.6%	0.75	98.2%	94.7%	0.81	98.8%	95.4%	0.93	99.6%
DRB1	58	E	4.6%	6.0%	0.64	96.9%	5.4%	0.75	98.2%	5.2%	0.81	98.8%	4.6%	0.93	99.6%
DRB1	57	D	75.3%	73.7%	0.68	92.0%	76.9%	0.80	95.5%	75.6%	0.90	97.4%	75.4%	0.98	99.4%
DRB1	57	V	19.0%	21.0%	0.68	94.0%	18.0%	0.82	97.1%	18.5%	0.93	98.5%	18.9%	0.98	99.7%
DRB1	57	S	3.0%	2.4%	0.63	98.7%	2.4%	0.66	98.8%	3.3%	0.70	98.7%	3.0%	0.97	99.8%
DRB1	57	A	2.7%	3.0%	0.45	97.7%	2.7%	0.85	99.3%	2.5%	0.76	99.2%	2.7%	0.93	99.8%
DRB1	47	F	43.0%	43.0%	0.84	94.1%	43.9%	0.89	96.4%	42.7%	0.95	98.5%	42.8%	0.97	99.4%
DRB1	47	Y	57.0%	57.0%	0.84	94.1%	56.1%	0.89	96.4%	57.3%	0.95	98.5%	57.2%	0.97	99.4%
DRB1	40	Y	0.8%	0.1%	0.00	99.1%	0.1%	0.00	99.1%	0.8%	1.00	100.0%	0.8%	1.00	100.0%
DRB1	40	F	99.2%	99.9%	0.00	99.1%	99.9%	0.00	99.1%	99.2%	1.00	100.0%	99.2%	1.00	100.0%
DRB1	38	V	97.4%	96.4%	0.34	97.3%	98.1%	0.64	99.0%	97.8%	0.90	99.6%	97.4%	1.00	100.0%
DRB1	38	L	1.7%	3.5%	0.52	98.3%	1.8%	0.97	99.9%	1.4%	0.86	99.6%	1.7%	1.00	100.0%

DRB1	38	A	0.8%	0.1%	0.00	99.1%	0.1%	0.00	99.0%	0.8%	1.00	100.0%	0.8%	1.00	100.0%
DRB1	37	N	24.5%	19.8%	0.73	92.5%	22.6%	0.81	95.3%	24.1%	0.90	97.6%	24.3%	0.97	99.4%
DRB1	37	S	26.3%	26.4%	0.85	95.4%	26.6%	0.85	95.5%	26.2%	0.99	99.7%	26.4%	1.00	99.9%
DRB1	37	Y	29.1%	30.1%	0.81	94.4%	30.2%	0.87	96.5%	30.1%	0.90	97.2%	29.3%	0.99	99.6%
DRB1	37	F	18.3%	20.3%	0.78	94.8%	18.8%	0.92	98.5%	18.2%	0.94	98.8%	18.3%	0.98	99.6%
DRB1	37	L	1.7%	3.5%	0.52	98.3%	1.8%	0.97	99.9%	1.4%	0.86	99.6%	1.7%	1.00	100.0%
DRB1	33	H	20.9%	21.3%	0.90	96.7%	22.3%	0.91	97.9%	21.1%	0.95	98.8%	21.1%	0.98	99.6%
DRB1	33	N	79.1%	78.7%	0.90	96.7%	77.7%	0.91	97.9%	78.9%	0.95	98.8%	78.9%	0.98	99.6%
DRB1	32	H	28.1%	26.8%	0.87	95.7%	27.2%	0.85	95.9%	27.3%	0.91	97.7%	28.0%	0.98	99.5%
DRB1	32	Y	71.9%	73.2%	0.87	95.7%	72.8%	0.85	95.9%	72.7%	0.91	97.7%	72.0%	0.98	99.5%
DRB1	31	F	85.6%	87.7%	0.78	97.0%	87.9%	0.79	97.0%	85.8%	0.95	99.2%	85.6%	1.00	100.0%
DRB1	31	I	13.6%	12.2%	0.71	96.2%	11.8%	0.72	96.5%	13.4%	0.95	99.2%	13.6%	1.00	100.0%
DRB1	31	V	0.8%	0.2%	0.00	99.0%	0.6%	0.00	98.6%	0.8%	1.00	100.0%	0.8%	1.00	100.0%
DRB1	30	Y	68.2%	66.8%	0.78	94.0%	69.5%	0.81	95.3%	68.8%	0.94	98.3%	68.3%	0.99	99.7%
DRB1	30	L	15.6%	17.3%	0.84	96.8%	16.2%	0.92	98.8%	15.6%	0.97	99.4%	15.6%	0.98	99.7%
DRB1	30	C	12.0%	12.2%	0.82	97.8%	11.8%	0.83	98.1%	11.8%	0.98	99.8%	12.0%	1.00	100.0%
DRB1	30	H	1.7%	3.5%	0.52	98.3%	1.8%	0.97	99.9%	1.4%	0.86	99.5%	1.7%	1.00	100.0%
DRB1	30	G	1.6%	NA	NA	NA	NA	NA	NA	1.6%	0.74	99.4%	1.6%	1.00	100.0%
DRB1	30	R	0.8%	0.2%	0.00	98.9%	0.6%	0.00	98.6%	0.8%	1.00	100.0%	0.8%	1.00	100.0%
DRB1	28	D	68.2%	66.8%	0.78	94.0%	69.3%	0.81	95.2%	68.5%	0.93	97.9%	68.3%	0.99	99.7%
DRB1	28	E	30.2%	33.2%	0.83	95.1%	30.7%	0.88	96.8%	30.0%	0.94	98.5%	30.1%	0.99	99.6%
DRB1	28	H	1.6%	NA	NA	NA	NA	NA	NA	1.6%	0.74	99.4%	1.6%	1.00	100.0%
DRB1	26	Y	15.6%	13.4%	0.78	95.0%	13.8%	0.77	95.8%	15.2%	0.94	98.9%	15.5%	0.97	99.4%
DRB1	26	F	69.9%	70.9%	0.74	92.4%	72.4%	0.86	95.5%	70.7%	0.94	98.3%	70.0%	0.98	99.5%
DRB1	26	L	14.5%	15.8%	0.80	96.8%	14.1%	0.91	98.3%	14.0%	0.97	99.4%	14.6%	1.00	100.0%
DRB1	25	Q	15.6%	17.3%	0.84	96.8%	16.2%	0.92	98.8%	15.6%	0.97	99.4%	15.6%	0.98	99.7%
DRB1	25	R	84.4%	82.7%	0.84	96.8%	83.8%	0.92	98.8%	84.4%	0.97	99.4%	84.4%	0.98	99.7%
DRB1	16	H	96.2%	96.8%	0.80	99.1%	96.3%	0.82	98.9%	95.7%	0.83	98.9%	96.0%	0.96	99.6%
DRB1	16	Y	3.8%	3.2%	0.80	99.1%	3.7%	0.82	98.9%	4.3%	0.84	98.9%	4.0%	0.96	99.6%
DRB1	14	K	15.6%	17.3%	0.84	96.8%	16.2%	0.92	98.8%	15.6%	0.97	99.4%	15.6%	0.98	99.7%
DRB1	14	E	84.4%	82.7%	0.84	96.8%	83.8%	0.92	98.8%	84.4%	0.97	99.4%	84.4%	0.98	99.7%
DRB1	13	S	30.9%	31.5%	0.87	94.6%	31.3%	0.85	95.0%	30.4%	0.96	98.6%	30.5%	0.98	99.4%
DRB1	13	R	14.4%	14.3%	0.90	97.0%	14.9%	0.86	96.7%	14.4%	1.00	99.9%	14.4%	1.00	99.9%

DRB1	13	H	20.9%	21.4%	0.90	96.6%	22.3%	0.91	97.9%	21.1%	0.95	98.8%	21.1%	0.98	99.6%
DRB1	13	Y	15.6%	17.3%	0.84	96.8%	16.2%	0.92	98.8%	15.6%	0.97	99.4%	15.6%	0.98	99.7%
DRB1	13	F	14.4%	12.5%	0.78	96.8%	12.1%	0.80	97.1%	14.2%	0.95	99.2%	14.4%	1.00	100.0%
DRB1	13	G	3.8%	3.2%	0.80	99.1%	3.4%	0.82	99.1%	4.3%	0.84	98.9%	4.0%	0.96	99.6%
DRB1	12	T	34.7%	34.6%	0.84	93.9%	34.7%	0.84	94.9%	34.7%	0.97	99.4%	34.5%	0.98	99.6%
DRB1	12	K	65.3%	65.4%	0.84	93.9%	65.3%	0.84	94.9%	65.3%	0.97	99.4%	65.5%	0.98	99.6%
DRB1	11	S	34.7%	34.6%	0.84	93.9%	34.7%	0.84	94.9%	34.7%	0.97	99.4%	34.5%	0.98	99.6%
DRB1	11	P	14.4%	14.5%	0.88	96.5%	15.0%	0.86	96.6%	14.4%	1.00	99.9%	14.4%	1.00	99.9%
DRB1	11	V	21.7%	21.4%	0.85	95.9%	22.3%	0.85	97.0%	21.9%	0.95	98.8%	22.0%	0.98	99.6%
DRB1	11	G	15.6%	17.3%	0.84	96.8%	16.2%	0.92	98.8%	15.6%	0.97	99.4%	15.6%	0.98	99.7%
DRB1	11	L	12.0%	12.2%	0.82	97.8%	11.8%	0.83	98.1%	11.8%	0.98	99.8%	12.0%	1.00	100.0%
DRB1	11	D	1.6%	NA	NA	NA	NA	NA	NA	1.6%	0.74	99.4%	1.6%	1.00	100.0%
DRB1	10	Y	34.7%	34.6%	0.84	93.9%	34.7%	0.84	94.8%	34.6%	0.97	99.4%	34.5%	0.98	99.6%
DRB1	10	Q	64.4%	65.1%	0.81	92.9%	65.2%	0.81	94.0%	64.6%	0.97	99.4%	64.7%	0.98	99.6%
DRB1	10	E	0.8%	0.1%	0.00	99.1%	0.1%	0.00	99.1%	0.8%	1.00	100.0%	0.8%	1.00	100.0%
DRB1	9	E	56.4%	55.8%	0.80	92.7%	57.1%	0.80	93.6%	56.5%	0.96	98.6%	56.4%	0.99	99.6%
DRB1	9	W	42.0%	44.2%	0.86	93.8%	42.9%	0.84	94.6%	41.9%	0.97	99.1%	42.0%	0.99	99.6%
DRB1	9	K	1.6%	NA	NA	NA	NA	NA	NA	1.6%	0.74	99.4%	1.6%	1.00	100.0%
DRB1	4	R	82.7%	82.7%	0.75	95.3%	83.8%	0.81	97.1%	82.4%	0.92	98.4%	82.7%	0.97	99.5%
DRB1	4	Q	17.2%	17.3%	0.76	95.5%	16.2%	0.82	97.2%	17.2%	0.95	98.9%	17.2%	0.98	99.7%
DRB1	4	x	0.1%	NA	NA	NA	NA	NA	NA	0.4%	0.00	99.5%	0.1%	0.00	99.8%
DRB1	-1	A	82.8%	83.5%	0.80	95.4%	83.7%	0.78	95.5%	82.7%	0.94	98.8%	82.9%	0.95	99.2%
DRB1	-1	S	13.8%	14.3%	0.88	96.8%	14.1%	0.85	96.9%	13.8%	0.99	99.8%	13.8%	1.00	99.9%
DRB1	-1	x	3.4%	2.2%	0.58	98.6%	2.2%	0.58	98.6%	3.5%	0.79	99.0%	3.3%	0.82	99.3%
DRB1	-16	V	32.2%	32.3%	0.83	93.4%	32.6%	0.78	93.7%	32.0%	0.96	98.7%	32.0%	0.97	99.2%
DRB1	-16	A	64.4%	65.5%	0.80	92.8%	65.2%	0.80	93.8%	64.5%	0.97	99.3%	64.7%	0.98	99.6%
DRB1	-16	x	3.4%	2.2%	0.58	98.6%	2.2%	0.58	98.6%	3.5%	0.79	99.0%	3.3%	0.82	99.3%
DRB1	-17	A	70.3%	71.0%	0.77	93.0%	71.1%	0.77	93.5%	70.3%	0.96	98.7%	70.3%	0.97	99.3%
DRB1	-17	T	26.3%	26.9%	0.82	94.1%	26.7%	0.81	94.6%	26.3%	0.98	99.6%	26.4%	0.99	99.9%
DRB1	-17	x	3.4%	2.2%	0.58	98.6%	2.2%	0.58	98.6%	3.5%	0.79	99.0%	3.3%	0.82	99.3%
DRB1	-24	L	75.7%	76.5%	0.87	96.1%	75.5%	0.89	97.6%	75.5%	0.93	98.0%	75.6%	0.96	99.1%
DRB1	-24	F	20.9%	21.3%	0.90	96.7%	22.4%	0.90	97.8%	21.1%	0.95	98.8%	21.1%	0.98	99.6%
DRB1	-24	x	3.4%	2.2%	0.58	98.6%	2.2%	0.58	98.6%	3.5%	0.79	99.0%	3.3%	0.82	99.3%

DRB1	-25	R	32.2%	32.3%	0.83	93.4%	32.6%	0.78	93.7%	32.0%	0.96	98.7%	32.0%	0.97	99.2%
DRB1	-25	K	64.4%	65.5%	0.80	92.8%	65.2%	0.80	93.8%	64.5%	0.97	99.3%	64.7%	0.98	99.6%
DRB1	-25	x	3.4%	2.2%	0.58	98.6%	2.2%	0.58	98.6%	3.5%	0.79	99.0%	3.3%	0.82	99.3%
DQB1	224	Q	83.7%	86.3%	0.62	93.1%	85.7%	0.61	93.4%	84.0%	0.94	98.9%	83.6%	0.97	99.4%
DQB1	224	R	16.2%	13.7%	0.63	93.2%	14.3%	0.62	93.6%	15.9%	0.96	99.1%	16.3%	0.98	99.6%
DQB1	224	x	0.2%	NA	NA	NA	NA	NA	NA	0.1%	0.00	99.8%	0.1%	0.00	99.7%
DQB1	221	H	61.1%	59.1%	0.64	88.5%	57.8%	0.61	88.7%	61.5%	0.98	99.3%	61.3%	0.97	99.1%
DQB1	221	Q	38.7%	40.9%	0.65	88.6%	42.2%	0.62	88.8%	38.5%	0.99	99.5%	38.6%	0.98	99.3%
DQB1	221	x	0.2%	NA	NA	NA	NA	NA	NA	0.0%	0.00	99.8%	0.1%	0.00	99.7%
DQB1	220	H	61.1%	59.1%	0.64	88.4%	57.8%	0.61	88.7%	61.5%	0.98	99.3%	61.3%	0.97	99.1%
DQB1	220	R	38.7%	40.9%	0.65	88.6%	42.2%	0.62	88.9%	38.5%	0.99	99.5%	38.6%	0.98	99.3%
DQB1	220	x	0.2%	NA	NA	NA	NA	NA	NA	0.0%	0.00	99.8%	0.1%	0.00	99.8%
DQB1	203	I	61.6%	59.1%	0.65	88.7%	57.8%	0.62	89.0%	62.0%	0.98	99.3%	61.7%	0.97	99.1%
DQB1	203	V	38.3%	40.9%	0.66	88.8%	42.2%	0.63	89.2%	38.0%	0.99	99.5%	38.2%	0.98	99.3%
DQB1	203	x	0.2%	NA	NA	NA	NA	NA	NA	0.0%	0.00	99.8%	0.1%	0.00	99.7%
DQB1	197	S	99.5%	100.0%	nan	99.5%	100.0%	nan	99.5%	99.5%	0.87	99.9%	99.5%	0.79	99.9%
DQB1	197	N	0.5%	0.0%	nan	99.5%	0.0%	nan	99.5%	0.5%	1.00	100.0%	0.5%	1.00	100.0%
DQB1	185	T	82.4%	84.0%	0.70	94.3%	84.5%	0.71	95.2%	82.4%	0.95	99.1%	82.4%	0.97	99.5%
DQB1	185	I	17.5%	16.0%	0.70	94.4%	15.5%	0.71	95.2%	17.5%	0.95	99.2%	17.5%	0.97	99.6%
DQB1	182	S	62.1%	62.7%	0.74	91.6%	63.9%	0.69	90.9%	62.4%	0.96	99.1%	62.4%	0.96	99.2%
DQB1	182	N	37.8%	37.3%	0.74	91.6%	36.1%	0.69	91.0%	37.5%	0.97	99.2%	37.5%	0.97	99.3%
DQB1	167	R	79.2%	78.7%	0.65	91.7%	79.4%	0.65	92.1%	79.5%	0.94	98.9%	79.5%	0.96	99.3%
DQB1	167	H	20.8%	21.3%	0.65	91.8%	20.6%	0.65	92.2%	20.5%	0.95	99.0%	20.5%	0.96	99.4%
DQB1	140	A	62.1%	62.8%	0.74	91.6%	63.9%	0.69	90.9%	62.4%	0.96	99.1%	62.4%	0.96	99.2%
DQB1	140	T	37.8%	37.2%	0.74	91.7%	36.1%	0.69	91.0%	37.5%	0.97	99.2%	37.5%	0.97	99.3%
DQB1	135	D	89.8%	91.1%	0.62	95.9%	90.2%	0.56	94.4%	89.5%	0.95	99.4%	89.5%	0.94	99.5%
DQB1	135	G	10.2%	8.9%	0.62	95.8%	9.8%	0.55	94.3%	10.5%	0.94	99.4%	10.4%	0.94	99.5%
DQB1	130	R	95.6%	96.3%	0.74	98.9%	94.6%	0.53	97.6%	95.6%	0.97	99.8%	95.6%	0.97	99.8%
DQB1	130	Q	4.3%	3.7%	0.75	99.0%	5.4%	0.54	97.6%	4.4%	0.99	99.9%	4.3%	1.00	99.9%
DQB1	126	Q	99.2%	99.5%	0.53	99.2%	98.7%	0.55	99.0%	99.2%	0.88	99.8%	99.1%	0.80	99.8%
DQB1	126	H	0.7%	0.5%	0.58	99.2%	1.3%	0.60	99.1%	0.7%	0.90	99.8%	0.8%	0.79	99.8%
DQB1	125	A	61.2%	59.1%	0.65	88.5%	57.9%	0.61	88.8%	61.5%	0.99	99.4%	61.3%	0.98	99.2%
DQB1	125	G	22.6%	27.3%	0.65	91.0%	27.9%	0.55	90.0%	22.5%	0.96	99.0%	22.4%	0.98	99.6%

DQB1	125	S	16.2%	13.7%	0.63	93.0%	14.2%	0.62	93.6%	15.9%	0.96	99.1%	16.3%	0.98	99.6%
DQB1	116	V	83.8%	86.4%	0.62	93.1%	85.8%	0.61	93.6%	84.0%	0.95	99.0%	83.7%	0.98	99.6%
DQB1	116	I	16.2%	13.6%	0.63	93.1%	14.2%	0.62	93.6%	15.9%	0.96	99.1%	16.3%	0.98	99.6%
DQB1	90	T	61.3%	58.7%	0.64	88.2%	60.2%	0.58	87.8%	61.5%	0.99	99.5%	61.3%	0.98	99.3%
DQB1	90	I	38.7%	41.3%	0.64	88.2%	39.8%	0.58	87.8%	38.4%	0.99	99.5%	38.6%	0.98	99.2%
DQB1	89	T	61.3%	58.7%	0.64	88.2%	60.2%	0.58	87.8%	61.5%	0.99	99.5%	61.3%	0.98	99.3%
DQB1	89	G	38.7%	41.3%	0.64	88.2%	39.8%	0.58	87.8%	38.4%	0.99	99.5%	38.6%	0.98	99.2%
DQB1	87	L	61.3%	58.7%	0.64	88.2%	60.2%	0.58	87.8%	61.5%	0.99	99.5%	61.3%	0.98	99.3%
DQB1	87	F	18.3%	23.8%	0.61	90.7%	22.2%	0.58	91.5%	18.1%	0.96	99.0%	18.1%	0.98	99.5%
DQB1	87	Y	20.5%	17.5%	0.65	92.1%	17.2%	0.60	92.1%	20.3%	0.97	99.1%	20.6%	0.98	99.6%
DQB1	86	E	61.3%	58.7%	0.64	88.2%	60.2%	0.58	87.8%	61.5%	0.99	99.5%	61.3%	0.98	99.3%
DQB1	86	A	34.4%	37.5%	0.62	87.9%	36.8%	0.61	88.8%	34.1%	0.99	99.4%	34.4%	0.98	99.2%
DQB1	86	G	4.3%	3.8%	0.76	98.9%	3.0%	0.58	98.0%	4.4%	0.99	99.9%	4.3%	1.00	99.9%
DQB1	85	L	61.3%	58.7%	0.64	88.2%	60.2%	0.58	87.8%	61.5%	0.99	99.5%	61.3%	0.98	99.3%
DQB1	85	V	38.7%	41.3%	0.64	88.2%	39.8%	0.58	87.8%	38.4%	0.99	99.5%	38.6%	0.98	99.2%
DQB1	84	Q	61.3%	58.7%	0.64	88.2%	60.2%	0.58	87.8%	61.5%	0.99	99.5%	61.3%	0.98	99.3%
DQB1	84	E	38.7%	41.3%	0.64	88.2%	39.8%	0.58	87.8%	38.4%	0.99	99.5%	38.6%	0.98	99.2%
DQB1	77	R	39.6%	35.4%	0.58	87.4%	37.0%	0.59	88.2%	39.9%	0.94	98.3%	40.1%	0.96	99.0%
DQB1	77	T	60.4%	64.6%	0.58	87.4%	63.0%	0.59	88.2%	60.1%	0.94	98.3%	59.9%	0.96	99.0%
DQB1	75	V	42.1%	37.3%	0.59	87.3%	38.9%	0.59	87.9%	42.5%	0.94	98.3%	42.6%	0.96	99.0%
DQB1	75	L	57.9%	62.7%	0.59	87.3%	61.1%	0.59	87.9%	57.5%	0.94	98.3%	57.4%	0.96	99.0%
DQB1	74	A	23.5%	21.7%	0.67	92.6%	22.8%	0.66	92.0%	24.0%	0.96	99.0%	23.8%	0.96	99.1%
DQB1	74	E	57.9%	62.7%	0.59	87.3%	60.8%	0.59	87.8%	57.5%	0.94	98.3%	57.4%	0.96	99.0%
DQB1	74	S	18.7%	15.6%	0.64	92.4%	16.1%	0.63	93.0%	18.5%	0.96	99.1%	18.8%	0.99	99.7%
DQB1	71	K	23.5%	21.7%	0.67	92.6%	22.8%	0.66	92.0%	24.0%	0.96	99.0%	23.8%	0.96	99.1%
DQB1	71	T	57.9%	62.7%	0.59	87.3%	61.0%	0.59	87.9%	57.5%	0.95	98.3%	57.4%	0.96	99.0%
DQB1	71	A	16.2%	13.7%	0.62	92.8%	14.2%	0.62	93.6%	15.9%	0.96	99.1%	16.3%	0.98	99.6%
DQB1	71	D	2.5%	1.9%	0.79	99.4%	1.9%	0.80	99.4%	2.6%	0.99	100.0%	2.6%	0.98	99.9%
DQB1	70	R	63.5%	60.5%	0.65	88.3%	61.2%	0.64	89.1%	63.8%	0.99	99.3%	63.5%	0.98	99.2%
DQB1	70	G	34.0%	37.5%	0.63	88.1%	36.9%	0.63	89.1%	33.6%	0.99	99.4%	33.9%	0.98	99.2%
DQB1	70	E	2.5%	1.9%	0.79	99.4%	1.9%	0.80	99.4%	2.6%	0.99	100.0%	2.6%	0.98	99.9%
DQB1	67	I	26.4%	23.6%	0.69	92.5%	24.8%	0.68	92.0%	27.0%	0.96	98.9%	26.8%	0.96	99.1%
DQB1	67	V	73.6%	76.4%	0.69	92.5%	75.2%	0.68	92.0%	73.0%	0.96	98.9%	73.2%	0.96	99.1%

DQB1	66	D	26.4%	23.6%	0.69	92.5%	24.8%	0.68	92.0%	27.0%	0.96	98.9%	26.8%	0.96	99.1%
DQB1	66	E	73.6%	76.4%	0.69	92.5%	75.2%	0.68	92.0%	73.0%	0.96	98.9%	73.2%	0.96	99.1%
DQB1	57	A	32.7%	31.5%	0.67	90.4%	32.4%	0.67	90.9%	33.2%	0.96	98.9%	33.0%	0.95	99.0%
DQB1	57	D	49.6%	53.1%	0.60	86.6%	53.4%	0.65	88.6%	48.9%	0.96	98.7%	49.1%	0.96	98.8%
DQB1	57	V	17.0%	15.1%	0.70	94.0%	13.2%	0.71	94.9%	17.1%	1.00	99.8%	17.0%	1.00	99.9%
DQB1	57	S	0.7%	0.4%	0.59	99.3%	1.0%	0.60	99.3%	0.8%	0.96	99.9%	0.8%	0.87	99.9%
DQB1	56	L	2.5%	1.9%	0.79	99.4%	1.9%	0.80	99.4%	2.5%	1.00	100.0%	2.5%	1.00	100.0%
DQB1	56	P	97.5%	98.1%	0.79	99.4%	98.1%	0.80	99.4%	97.5%	1.00	100.0%	97.5%	1.00	100.0%
DQB1	55	L	23.5%	21.7%	0.67	92.6%	22.8%	0.66	92.0%	24.0%	0.96	99.0%	23.8%	0.96	99.1%
DQB1	55	R	41.2%	43.1%	0.65	88.2%	41.7%	0.59	87.7%	41.0%	0.99	99.4%	41.2%	0.98	99.3%
DQB1	55	P	35.3%	35.2%	0.74	91.8%	35.5%	0.69	90.6%	35.0%	0.97	99.2%	35.0%	0.97	99.3%
DQB1	53	Q	38.7%	41.2%	0.63	88.1%	39.8%	0.58	87.8%	38.5%	0.99	99.5%	38.7%	0.98	99.3%
DQB1	53	L	61.3%	58.8%	0.63	88.1%	60.2%	0.58	87.8%	61.5%	0.99	99.5%	61.3%	0.98	99.3%
DQB1	52	L	23.5%	21.7%	0.67	92.6%	22.9%	0.66	92.0%	24.0%	0.96	99.0%	23.8%	0.96	99.1%
DQB1	52	P	76.5%	78.3%	0.67	92.6%	77.1%	0.66	92.0%	76.0%	0.96	99.0%	76.2%	0.96	99.1%
DQB1	47	F	23.5%	21.7%	0.67	92.6%	22.9%	0.66	92.0%	24.0%	0.96	99.0%	23.8%	0.96	99.1%
DQB1	47	Y	76.5%	78.3%	0.67	92.6%	77.1%	0.66	92.0%	76.0%	0.96	99.0%	76.2%	0.96	99.1%
DQB1	46	E	23.5%	21.7%	0.67	92.6%	22.9%	0.66	92.0%	24.0%	0.96	99.0%	23.8%	0.96	99.1%
DQB1	46	V	76.5%	78.3%	0.67	92.6%	77.1%	0.66	92.0%	76.0%	0.96	99.0%	76.2%	0.96	99.1%
DQB1	45	E	20.3%	21.1%	0.67	92.2%	21.8%	0.64	91.6%	20.0%	0.95	99.0%	20.0%	0.96	99.4%
DQB1	45	G	79.7%	78.9%	0.67	92.2%	78.2%	0.64	91.6%	80.0%	0.95	99.0%	80.0%	0.96	99.4%
DQB1	38	V	40.1%	35.4%	0.59	87.5%	37.0%	0.60	88.4%	40.4%	0.95	98.3%	40.6%	0.96	99.0%
DQB1	38	A	59.9%	64.6%	0.59	87.5%	63.0%	0.60	88.4%	59.6%	0.95	98.3%	59.4%	0.96	99.0%
DQB1	37	I	23.5%	21.7%	0.67	92.6%	22.9%	0.66	92.0%	24.0%	0.96	99.0%	23.8%	0.96	99.1%
DQB1	37	Y	76.1%	78.3%	0.69	92.7%	77.1%	0.67	92.2%	75.5%	0.96	99.0%	75.7%	0.96	99.1%
DQB1	37	D	0.5%	0.0%	nan	99.5%	0.0%	nan	99.5%	0.5%	1.00	100.0%	0.5%	1.00	100.0%
DQB1	30	S	23.5%	21.7%	0.67	92.6%	22.9%	0.66	92.0%	24.0%	0.96	99.0%	23.8%	0.96	99.1%
DQB1	30	Y	51.9%	57.6%	0.57	85.8%	55.2%	0.57	86.3%	51.8%	0.93	97.6%	51.3%	0.95	98.3%
DQB1	30	H	24.7%	20.8%	0.62	89.7%	22.0%	0.61	90.4%	24.2%	0.94	98.1%	24.9%	0.96	98.9%
DQB1	28	S	23.5%	21.7%	0.67	92.6%	22.9%	0.66	92.0%	24.0%	0.96	99.0%	23.8%	0.96	99.1%
DQB1	28	T	76.5%	78.3%	0.67	92.6%	77.1%	0.66	92.0%	76.0%	0.96	99.0%	76.2%	0.96	99.1%
DQB1	26	L	60.5%	63.0%	0.70	90.3%	62.0%	0.65	89.7%	61.0%	0.94	98.3%	60.7%	0.98	99.5%
DQB1	26	Y	20.8%	21.0%	0.65	91.9%	21.8%	0.63	91.4%	20.5%	0.95	99.0%	20.5%	0.96	99.4%

DQB1	26	G	18.7%	15.9%	0.64	92.2%	16.3%	0.63	92.9%	18.5%	0.96	99.0%	18.9%	0.98	99.6%
DQB1	14	M	83.8%	86.3%	0.61	92.7%	85.9%	0.62	93.7%	84.0%	0.96	99.1%	83.7%	0.98	99.7%
DQB1	14	L	16.2%	13.7%	0.61	92.7%	14.1%	0.62	93.7%	15.9%	0.96	99.1%	16.3%	0.98	99.7%
DQB1	13	G	79.2%	78.9%	0.65	91.9%	78.2%	0.63	91.4%	79.5%	0.95	99.0%	79.5%	0.96	99.4%
DQB1	13	A	20.8%	21.1%	0.65	91.9%	21.8%	0.63	91.4%	20.5%	0.95	99.0%	20.5%	0.96	99.4%
DQB1	9	Y	84.5%	78.4%	0.56	90.1%	80.2%	0.60	91.7%	84.3%	0.92	98.3%	84.8%	0.96	99.0%
DQB1	9	F	15.1%	21.6%	0.58	90.3%	19.7%	0.63	92.1%	15.3%	0.92	98.3%	14.7%	0.96	99.0%
DQB1	9	L	0.5%	0.0%	nan	99.5%	0.0%	nan	99.5%	0.5%	1.00	100.0%	0.5%	1.00	100.0%
DQB1	3	S	99.5%	100.0%	nan	99.5%	100.0%	nan	99.5%	99.5%	0.87	99.9%	99.5%	0.79	99.9%
DQB1	3	P	0.5%	0.0%	nan	99.5%	0.0%	nan	99.5%	0.5%	1.00	100.0%	0.5%	1.00	100.0%
DQB1	-4	V	61.6%	58.8%	0.64	88.1%	59.6%	0.61	88.3%	62.0%	0.98	99.3%	61.7%	0.97	99.1%
DQB1	-4	L	35.0%	38.4%	0.63	88.0%	37.0%	0.60	88.4%	34.6%	0.99	99.3%	34.9%	0.98	99.2%
DQB1	-4	x	3.4%	2.8%	0.69	98.8%	3.4%	0.70	98.5%	3.4%	0.93	99.7%	3.3%	0.93	99.7%
DQB1	-5	P	61.6%	58.7%	0.63	88.0%	59.8%	0.61	88.2%	62.0%	0.98	99.3%	61.7%	0.97	99.1%
DQB1	-5	L	18.8%	24.8%	0.63	90.8%	22.8%	0.56	91.1%	18.7%	0.96	99.0%	18.6%	0.98	99.5%
DQB1	-5	x	3.4%	2.8%	0.69	98.8%	3.4%	0.70	98.5%	3.4%	0.93	99.7%	3.3%	0.93	99.7%
DQB1	-5	S	16.2%	13.7%	0.61	92.7%	14.1%	0.62	93.7%	15.9%	0.96	99.1%	16.3%	0.98	99.6%
DQB1	-6	T	61.6%	58.7%	0.63	88.0%	59.7%	0.61	88.2%	62.0%	0.98	99.3%	61.7%	0.97	99.1%
DQB1	-6	S	35.0%	38.6%	0.63	87.9%	37.0%	0.60	88.3%	34.6%	0.99	99.3%	34.9%	0.98	99.2%
DQB1	-6	x	3.4%	2.8%	0.69	98.8%	3.4%	0.70	98.5%	3.4%	0.93	99.7%	3.3%	0.93	99.7%
DQB1	-9	M	80.4%	83.5%	0.62	91.8%	82.5%	0.64	92.8%	80.7%	0.95	98.9%	80.4%	0.97	99.5%
DQB1	-9	x	3.4%	2.8%	0.69	98.7%	3.4%	0.69	98.4%	3.4%	0.93	99.7%	3.3%	0.93	99.7%
DQB1	-9	I	16.2%	13.7%	0.61	92.7%	14.1%	0.62	93.7%	15.9%	0.96	99.1%	16.3%	0.98	99.6%
DQB1	-10	S	23.4%	21.5%	0.66	92.5%	22.9%	0.65	91.9%	24.0%	0.96	98.9%	23.8%	0.96	99.0%
DQB1	-10	A	73.2%	75.7%	0.67	91.6%	73.6%	0.64	90.8%	72.6%	0.96	98.7%	72.9%	0.96	98.9%
DQB1	-10	x	3.4%	2.8%	0.69	98.8%	3.4%	0.69	98.5%	3.4%	0.93	99.7%	3.3%	0.93	99.7%
DQB1	-17	A	96.1%	97.2%	0.61	98.3%	96.6%	0.61	97.9%	96.2%	0.94	99.7%	96.2%	0.94	99.7%
DQB1	-17	x	3.4%	2.8%	0.69	98.7%	3.4%	0.69	98.4%	3.4%	0.93	99.7%	3.3%	0.93	99.7%
DQB1	-17	P	0.5%	0.0%	nan	99.5%	0.0%	nan	99.5%	0.5%	1.00	100.0%	0.5%	1.00	100.0%
DQB1	-18	A	44.0%	42.6%	0.61	87.2%	44.2%	0.61	88.0%	44.5%	0.96	98.7%	44.2%	0.97	98.8%
DQB1	-18	V	52.5%	54.6%	0.61	86.7%	52.4%	0.61	87.8%	52.1%	0.97	98.7%	52.4%	0.97	98.9%
DQB1	-18	x	3.4%	2.8%	0.69	98.8%	3.4%	0.69	98.4%	3.4%	0.93	99.7%	3.3%	0.93	99.7%
DQB1	-21	G	61.6%	58.7%	0.63	88.0%	59.6%	0.61	88.3%	62.0%	0.98	99.3%	61.7%	0.97	99.1%

DQB1	-21	D	35.0%	38.5%	0.63	87.9%	36.9%	0.60	88.2%	34.6%	0.99	99.3%	34.9%	0.98	99.2%
DQB1	-21	x	3.4%	2.8%	0.69	98.7%	3.4%	0.69	98.4%	3.4%	0.93	99.7%	3.3%	0.93	99.7%
DQB1	-27	A	80.4%	83.5%	0.62	91.8%	82.5%	0.64	92.8%	80.7%	0.95	98.9%	80.4%	0.97	99.5%
DQB1	-27	x	3.4%	2.8%	0.69	98.8%	3.3%	0.69	98.5%	3.4%	0.93	99.7%	3.3%	0.93	99.7%
DQB1	-27	S	16.2%	13.7%	0.61	92.7%	14.1%	0.62	93.7%	15.9%	0.96	99.1%	16.3%	0.98	99.7%

Table S5: Haplotype risk analysis of WTCCC type 1 diabetes data. Since our imputation method provided phasing, we assessed the risk of haplotypes spanning *HLA-DRB1*, *HLA-DQA1* and *HLA-DQB1* and compared these to the estimated odds ratios reported by Cucca et al. (*Human Molecular Genetics*, 2001). The odds ratios of Cucca et al. were estimated based on allelic transmission/non-transmission from familial data, whereas our odds ratios were estimated from case/control data collected by WTCCC (*Nature*, 2007). We follow the same risk group classification as suggested by Cucca et al. The odds ratio is given relative to the reference haplotype, DRB1*01-DQA1*0101-DQB1*0501. One haplotype, DRB1*0701-DQA1*0201-DQB1*0201, was omitted from this analysis because of zero allele count in our control data.

**Haplotypes**				**Previously reported risk (Cucca et al.)**		**Estimated risk (this study)**	
**T1D risk**	***DRB1***	***DQA1***	***DQB1***	**ORT**	**95% CI**	**OR**	**95% CI**
Very high	0405	0301	0302	10.8	5.6-20.6	4.2	2.7-6.5
	0401	0301	0301	7.2	4.6-11.5	4.7	3.9-5.6
	0301	0501	0201	4.3	2.9-6.3	2.7	2.3-3.1
	0404	0301	0302	4.1	2.3-7.1	1.9	1.6-2.3
	0402	0301	0302	3.1	1.4-6.8	4.7	2.7-8.2
Intermediate	0405	0301	0201	2.0	0.7-5.6	6.5	1.4-31.0
	08	0401	0402	1.6	0.8-3.1	1.2	0.9-1.6
	1302	0102	0604	1.3	0.6-2.7	1.0	0.8-1.3
	0901	0301	0303	1.2	0.5-2.9	1.1	0.7-1.6
	**01**	**0101**	**0501**	**1**		**1**	
	1601	0102	0502	0.8	0.5-1.2	0.8	0.5-1.6

	0401	0301	0301	0.8	0.4-1.5	0.7	0.6-0.9
	0405	0501	0301	0.4	0.1-1.3	3.3	0.3-36.2
	0403	0301	0302	0.5	0.1-1.4	1.2	0.4-3.7
	1301	0103	0603	0.4	0.2-1.1	0.25	0.17-0.35
	1001	0101	0501	0.2	0.05-0.9	0.19	0.07-0.55
	1303-1305	0501	0301	0.3	0.1-1.2	0.15	0.06-0.37
Very low	11-12	0501	0301	0.2	0.1-0.3	0.28	0.21-0.36
	1501	0102	0602	0.04	0.01-0.1	0.013	0.0057-0.029
	0701	0201	0303	0.1	0.01-0.6	0.062	0.032-0.12
	1401	01	0503	0.1	0.01-0.4	0.054	0.020-0.15

Figure S1: Density of SNPs across the MHC for the Type 1 Diabetes Genetics Consortium reference panel (green), HapMap-CEPH reference panel (black), Illumina Immunochip microarray (red), and the Affymetrix GeneChip 500K microarray (blue). The T1DGC reference panel and Illumina Immunochip have the highest SNP density, while the Affymetrix 500K array has the lowest SNP density.

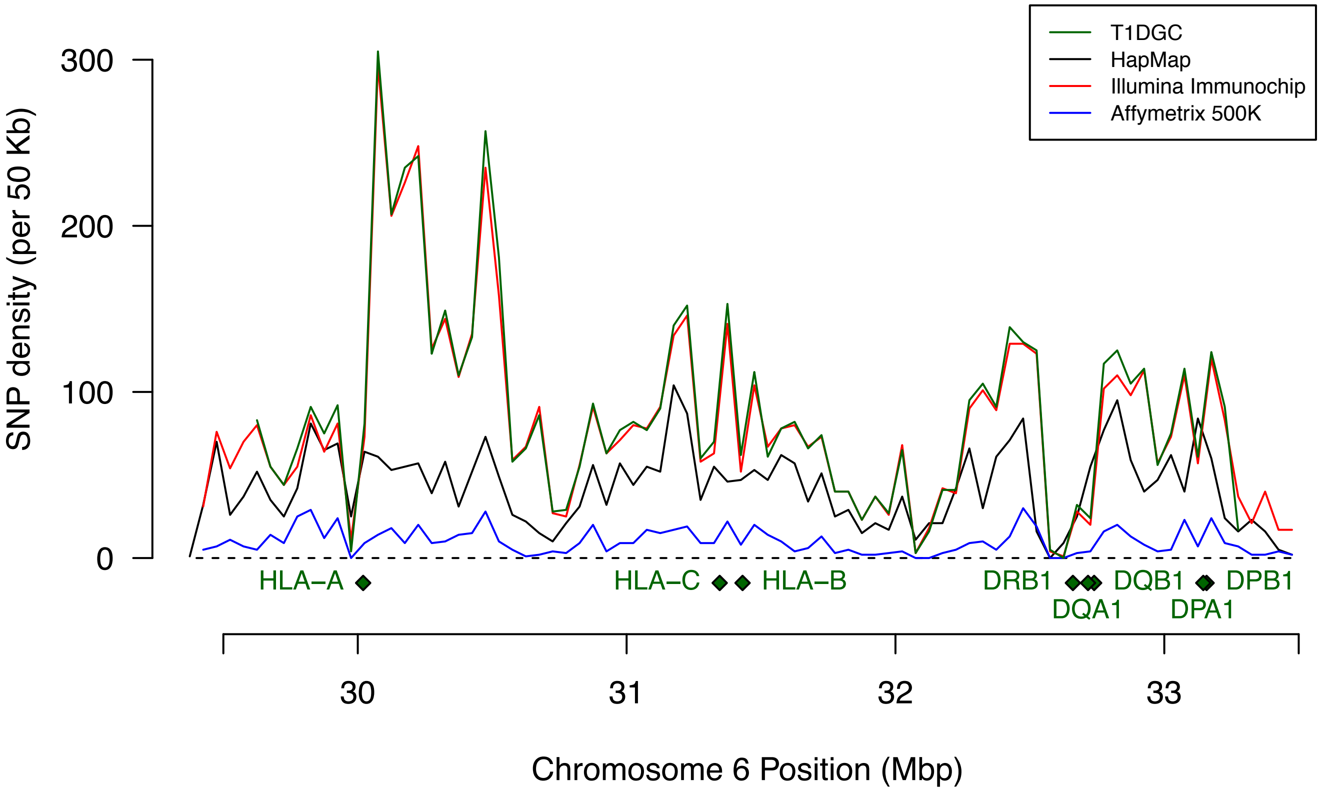

Figure S2: Imputed vs. typed frequencies of classical HLA alleles in the B58BC for imputation from the (a) Affymetrix 500K or (b) Illumina Immunochip platform using the HapMap-CEPH reference panel, and imputation from the (c) Affymetrix 500K or (d) Illumina Immunochip platform using the T1DGC reference panel. Black points indicate 2-digit HLA alleles. Red points indicate 4-digit HLA alleles.

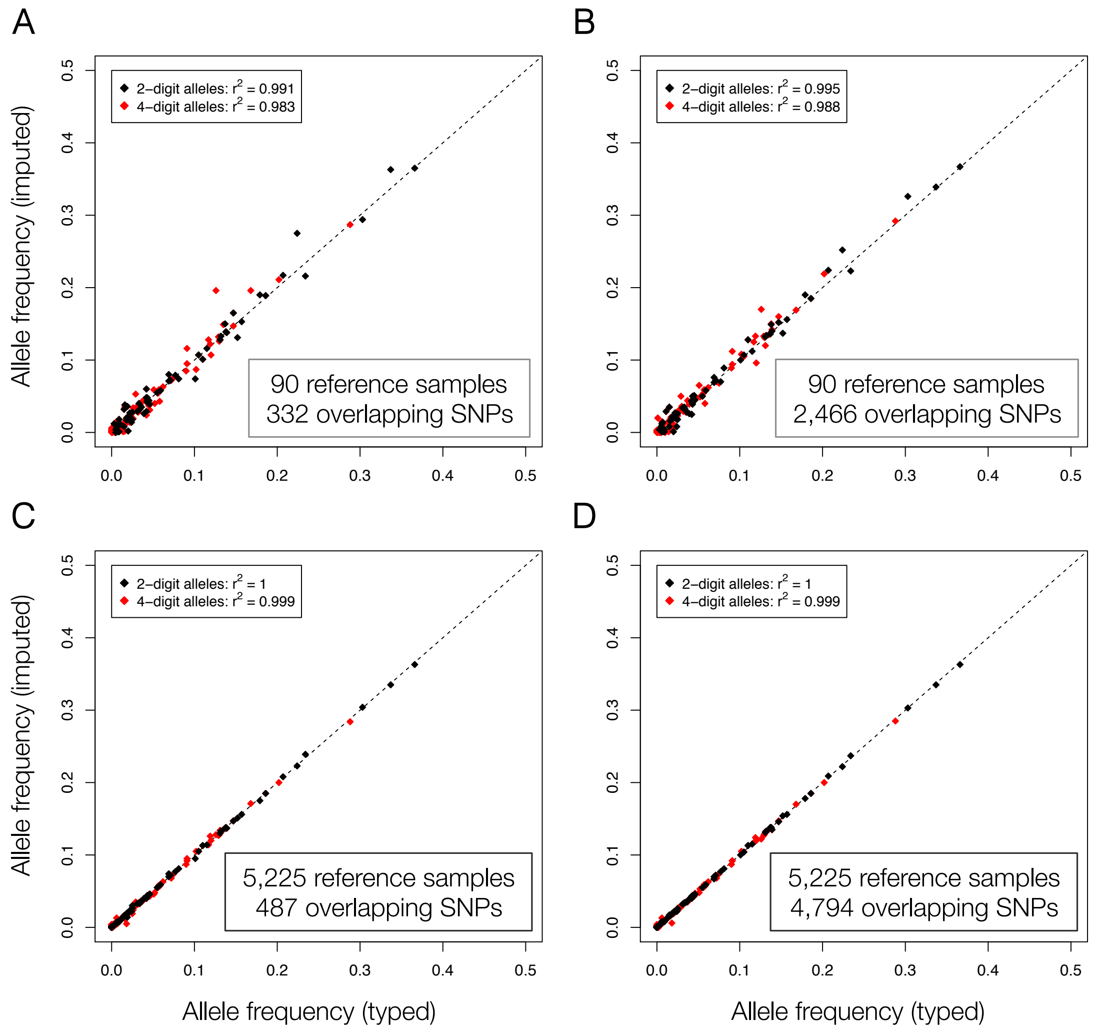

Figure S3: Imputed vs. typed frequencies of polymorphic amino acids in the B58BC for imputation from the (a) Affymetrix 500K or (b) Illumina Immunochip platform using the HapMap-CEPH reference panel, and imputation from the (c) Affymetrix 500K or (d) Illumina Immunochip platform using the T1DGC reference panel. Black points indicate bi-allelic positions. Red points indicate poly-allelic positions.

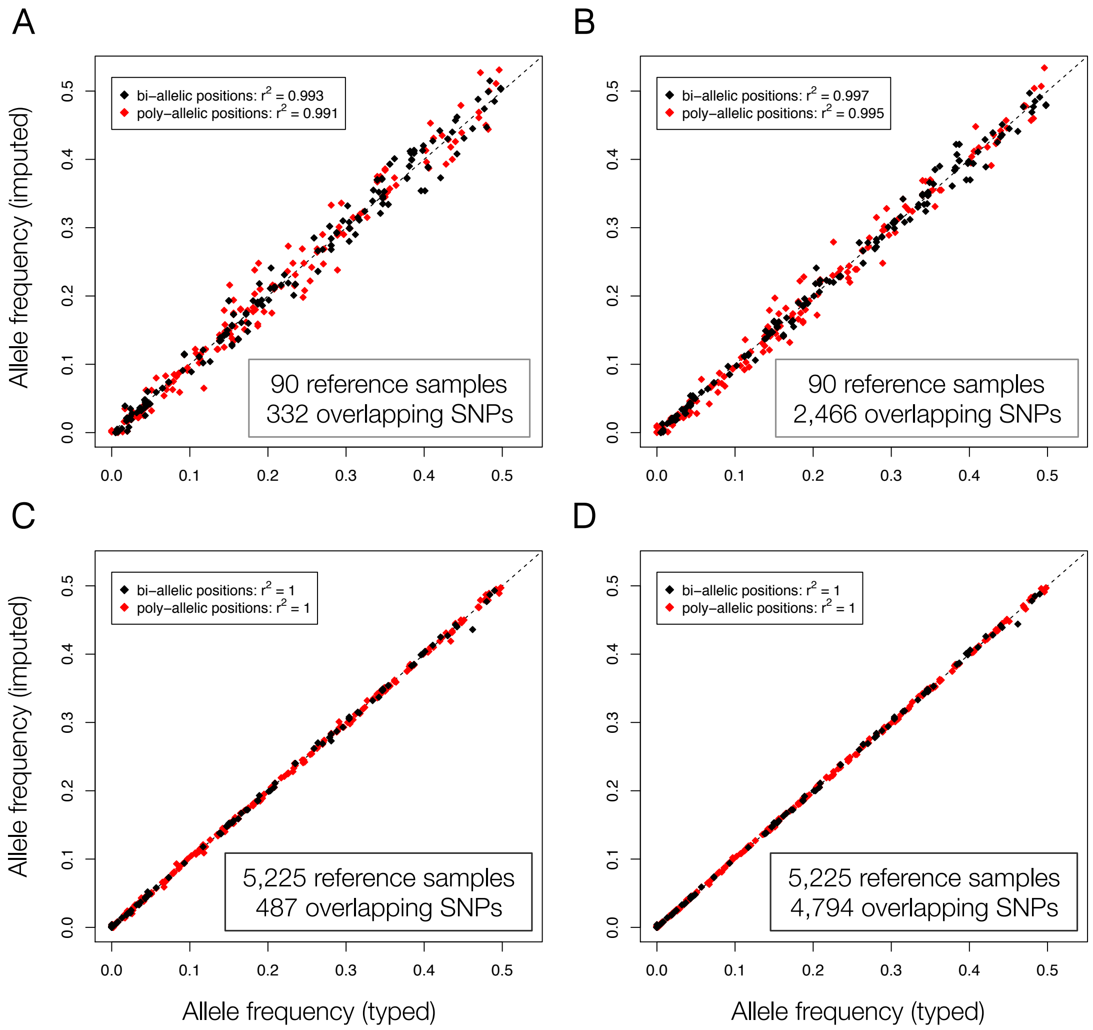

Figure S4: Genotyped (true) dosage closely tracks imputed dosage for classical HLA alleles and polymorphic amino acids. Mean and standard deviation for each 0.2 dosage bin is shown. Grey bars indicate the number of observations for each dosage bin. Data is shown for HLA imputations (a) starting from Immunochip SNPs using the HapMap-CEPH reference panel, and (b) starting from Immunochip SNPs using the T1DGC reference panel. The imputations with the larger T1DGC panel has a noticeably lower number of variants imputed at intermediate dosages between 0 and 1 and between 1 and 2, reflecting a greater confidence for the quality of the imputations.

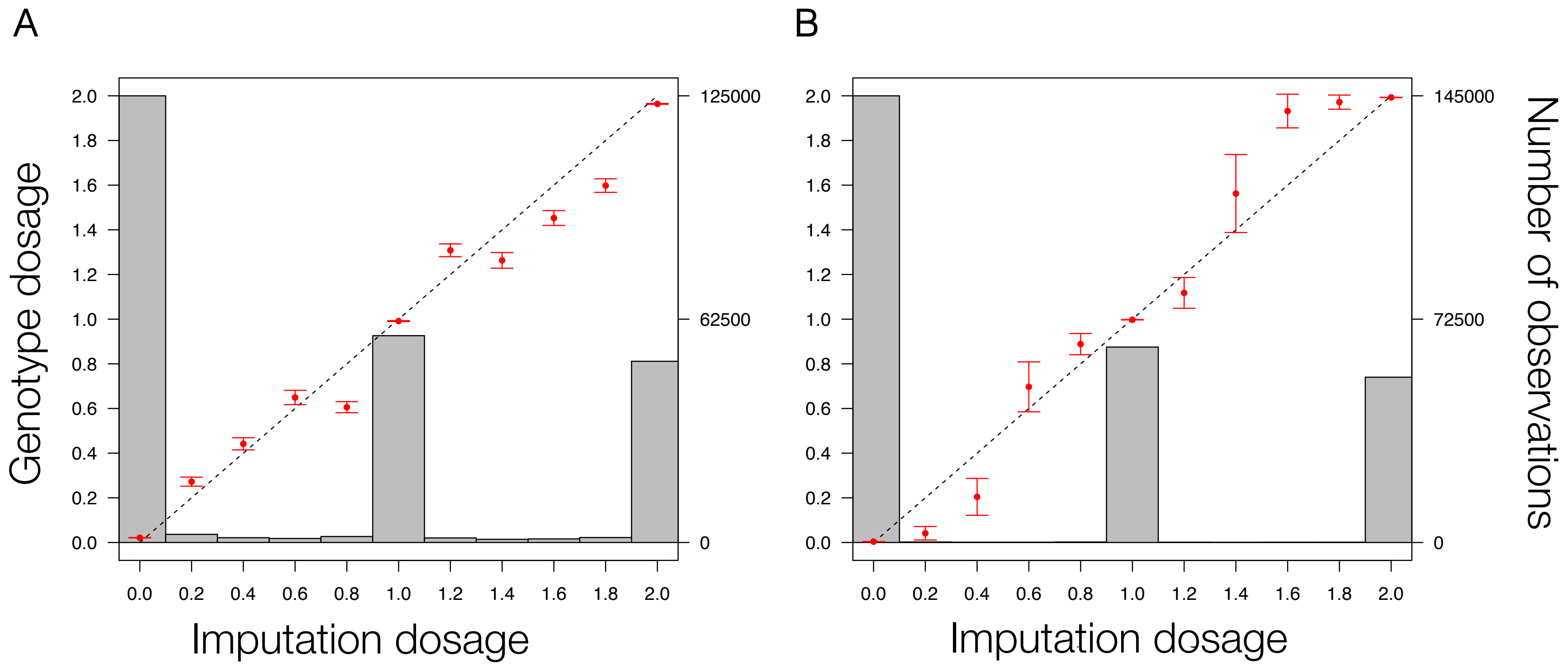

Supplement: File S1 — Tables S1–S5. Figures S1–S4. (PDF) [file pone.0064683.s001.pdf]
